# RNA virus polymerase-helicase coupling enables rapid elongation through duplex RNA

**DOI:** 10.1016/j.celrep.2026.117273

**Published:** 2026-04-16

**Authors:** Pim P.B. America, Subhas C. Bera, Arnab Das, Thomas K. Anderson, John C. Marecki, Flávia S. Papini, Jamie J. Arnold, Robert N. Kirchdoerfer, Craig E. Cameron, Kevin D. Raney, Martin Depken, David Dulin

**Affiliations:** 1 Department of Physics and Astronomy, De Boelelaan 1081, 1081 HV Amsterdam, the Netherlands; 2 Junior Research Group 2, Interdisciplinary Center for Clinical Research, Friedrich-Alexander-University Erlangen-Nürnberg (FAU), Cauerstr. 3, 91058 Erlangen, Germany; 3 Department of Biochemistry and Institute for Molecular Virology, University of Wisconsin-Madison, Madison, WI 53706, USA; 4 Department of Biochemistry and Molecular Biology, University of Arkansas for Medical Sciences, Little Rock, AR 72205, USA; 5 Department of Microbiology and Immunology, University of North Carolina School of Medicine, Chapel Hill, NC 27599, USA; 6 Department of Bionanoscience, Kavli Institute of Nanoscience, Delft University of Technology, Van der Maasweg 9, 2629 HZ Delft, the Netherlands; 7 These authors contributed equally; 8 Lead contact

## Abstract

Positive-sense RNA ((+)RNA) viruses often encode helicases presumed to support replication. Their precise role remains unresolved, though, especially in coronaviruses (CoVs), where the helicase translocates in the opposite direction to the polymerase. Using high-throughput single-molecule magnetic tweezers, we show that the coronavirus helicase enhances RNA synthesis through duplex RNA by 10-fold, forming a directional complex with the viral polymerase. Despite opposing polarity, the helicase coordinates elongation by engaging with the non-template strand. A detailed kinetic model derived from large datasets reveals distinct dynamic states, including fast-bursting and slow, backtracking-prone modes, which are governed by helicase engagement. These results uncover an active coupling mechanism that modulates replication dynamics and provide a mechanistic basis for continuous versus discontinuous RNA synthesis in coronaviruses. Our findings establish the viral helicase as a central regulator of RNA replication.

## In brief

America, Bera, et al. show that the CoV helicase increases polymerase RNA synthesis through duplex RNA by 10-fold and present a model linking synthesis rate to elongation complex composition. This work provides direct evidence that a helicase supports polymerase elongation for a positive-strand RNA virus.

## Graphical Abstract

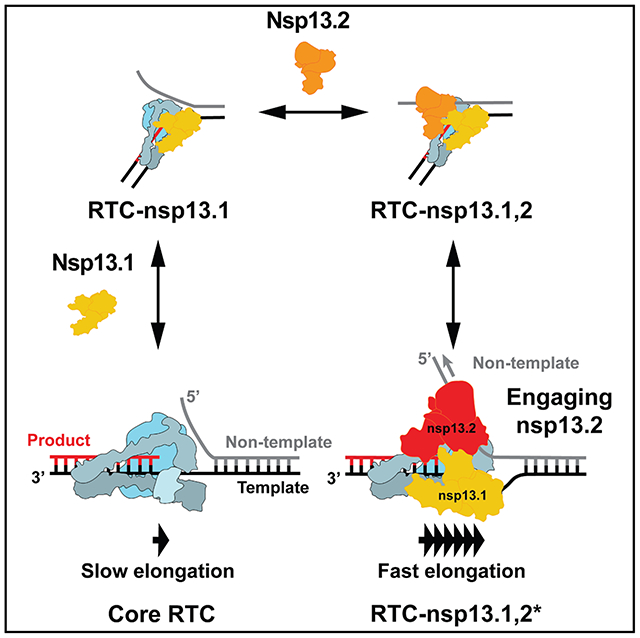

## INTRODUCTION

Positive-sense RNA ((+)RNA) viruses have a single-stranded (ss)RNA genome that directly encodes for the viral proteins, such as a helicase.^1,2^ These helicases are monomeric or hexameric and hydrolyze NTP, and some have been shown to translocate on ssRNA and displace double-stranded (ds)RNA on their own.^3^ They have been proposed to support viral RNA synthesis by associating with the viral RNA-dependent RNA polymerase (RdRp), though this hypothesis has never been directly demonstrated.^3^ In some (+)RNA virus families, such as alphavirus (e.g., chikungunya) and coronavirus (CoV; e.g., SARS-CoV-1, SARS-CoV-2, and MERS-CoV), the helicase even translocates in the opposite direction of the RdRp. This raises questions regarding how these helicases assist their RdRp during replication. While such polymerase-helicase complexes have been structurally resolved,^4–6^ there is a lack of functional studies reporting their precise role and specifically whether they assist replication.

**Figure 1.**
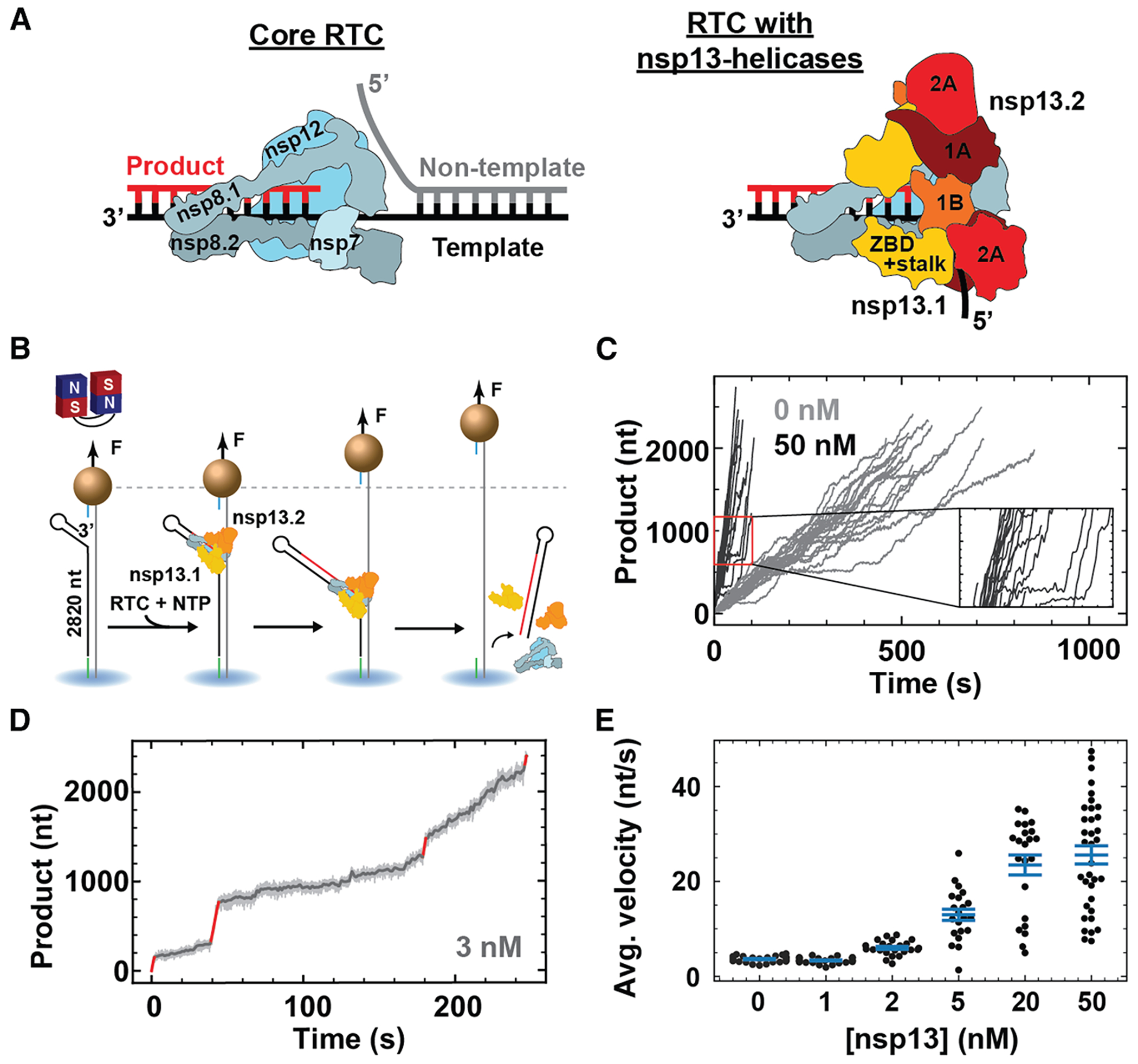
The SARS-CoV-2 nsp13 helicase enables fast RTC elongation on a dsRNA template (A) Core RTC (left) and RTC with two nsp13 helicases (right). Core RTC subunits and nsp13-helicase domains are indicated. (B) Schematic of the magnetic tweezers assay to monitor the dsRNA elongation of the RTC in complex with nsp13 helicase. (C) Elongation traces obtained without (gray) or with (black) 50 nM nsp13 helicase at 20 pN tension on the dsRNA template. Inset: zoom-in of the traces as indicated with the red rectangle. Slow elongation dynamics intervals interrupt long bursts of very fast nucleotide addition. (D) RTC elongation trace obtained at 3 nM nsp13 helicase (raw data: gray; filtered data: dark gray). The bursts of very fast nucleotide addition are highlighted in red in the filtered trace. (E) Average elongation velocity versus nsp13-helicase concentration from traces longer than 1,000 nt (dots). Mean values and error bars (standard error of the mean [SEM]) are indicated in blue. The RNA tension was 20 pN for all data shown.

We investigated the replication-transcription complex (RTC) from SARS-CoV-2, the causative agent of the COVID-19 pandemic. Extensive structural studies have established that the RTC is composed of a core made of the non-structural protein (nsp)12 polymerase, associated with the co-factors nsp7 and nsp8 in a 1:1:2 stoichiometry (Figure 1A).^7–9^ This core RTC may bind either one (nsp13.1) or two (nsp13.1 and nsp13.2) nsp13 helicases (Figure 1A).^4,10,11^ Nsp13 helicase has the typical structure of the superfamily 1B helicases, where the single-stranded nucleic acid is sandwiched between the 1B and stalk domains on one side and the two RecA domains (RecA1 and RecA2) on the other side (Figure 1A).^12^ Single-molecule studies have reported rapid and processive dsRNA unwinding by nsp13 helicase when mechanically assisted.^13,14^ Cryo-electron microscopy (EM) studies have reported that only nsp13.1 interacts with the template strand (Figure 1A).^4,10^ Due to the opposite polarity between nsp13 helicase and nsp12 polymerase, nsp13.1 is expected to push the RdRp backward, inducing RdRp backtracking. The backtracked state has been proposed as an intermediate for sub-genomic RNA synthesis, viral RNA recombination, and proofreading.^4^ However, no specific function has been attributed to nsp13.2, apart from allosterically controlling the productive engagement of nsp13.1 with the template RNA.^15^ Downstream duplex RNA represents a mechanical barrier to the elongating core RTC, resulting in an increased probability of long-lived pauses (LLPs) and very slow overall RNA synthesis.^16^ This suggests that one of the two nsp13 helicases may be actively supporting the CoV RTC during replication of the heavily structured CoV genomic RNA,^17^ although this has never been demonstrated.

We employed high-throughput magnetic tweezers to investigate how nsp13 helicase associates with the CoV RTC and impacts the dynamics of RNA synthesis. We show that nsp13 helicase specifically associates with the elongating core RTC and not with the polymerase in solution. Furthermore, of the two associated helicases, nsp13.2 enables rapid RNA synthesis through long dsRNA by translocating on the non-template strand. We show that three different complexes, populated as a function of nsp13-helicase concentration, are capable of RNA synthesis and co-exist at equilibrium: the core RTC alone, the core RTC associated with nsp13.1, and the core RTC associated with nsp13.1 and nsp13.2. We modeled that, in complex with the RTC, nsp13.2 can be either non-engaged or engaged with the non-template RNA, and only the engaged mode enables very fast RTC elongation. Mechanochemical analysis shows that nsp13.2 also increases the elongation rate via allostery when binding the RTC. Our work establishes a quantitative link between CoV RTC assembly and function. Specifically, we provide a functional demonstration of a novel role for (+)RNA virus helicases in supporting polymerase elongation through structured RNA.

## RESULTS

### The nsp13 helicase promotes rapid RNA synthesis on a dsRNA template by the CoV RTC

We employed a high-throughput magnetic tweezers assay to monitor CoV RTC elongation on dsRNA, as previously described for poliovirus (PV) RdRp.^18,19^ Briefly, a pair of permanent magnets located above a flow chamber is used to apply an attractive force to the magnetic beads. The force stretches the dsRNA tethers that attach each bead to the glass surface of the flow chamber (Figure 1A; STAR Methods).^20^ After flushing the reaction buffer containing the viral proteins and NTPs (STAR Methods), the RTC assembles on a 3′ end ssRNA loading site at a small hairpin. Once assembled, the RTC elongates through the dsRNA until reaching the end of the 2,820-nt-long template strand (Figure 1B; Table S1). The RTC elongation activity converts the dsRNA tether into ssRNA by displacing the template from the non-template strand, resulting in an increase of the tether extension (Figure 1B).^21^ The force was kept constant throughout the experiment by maintaining the magnets at a constant height above the flow chamber.^22,23^ In the absence of nsp13 helicase, we found that the core RTC elongates slowly on dsRNA at 20 pN tension, with an average velocity of (3.6 ± 0.1) nt/s (Figures 1C and 1E). Adding 50 nM of nsp13 helicase to the reaction buffer dramatically increases the average elongation velocity to (25.6 ± 2.0) nt/s (Figures 1C and 1E). This shows that nsp13 helicase supports RNA synthesis through duplex RNA. By decreasing nsp13-helicase concentration to 3 nM, we can distinguish very fast nucleotide addition (VFNA) bursts (∼30 nt/s) of tens to hundreds of nucleotides long that are interrupted by slow nucleotide addition (SNA) cycles (Figures 1D and 1E). The average elongation velocity increases when the concentration of nsp13 helicase is varied from 0 to 50 nM, saturating above 10 nM (Figure 1E).

### Nsp13 helicases specifically associate with the CoV core RTC, and nsp13.2 translocates on the non-template RNA strand

**Figure 2.**
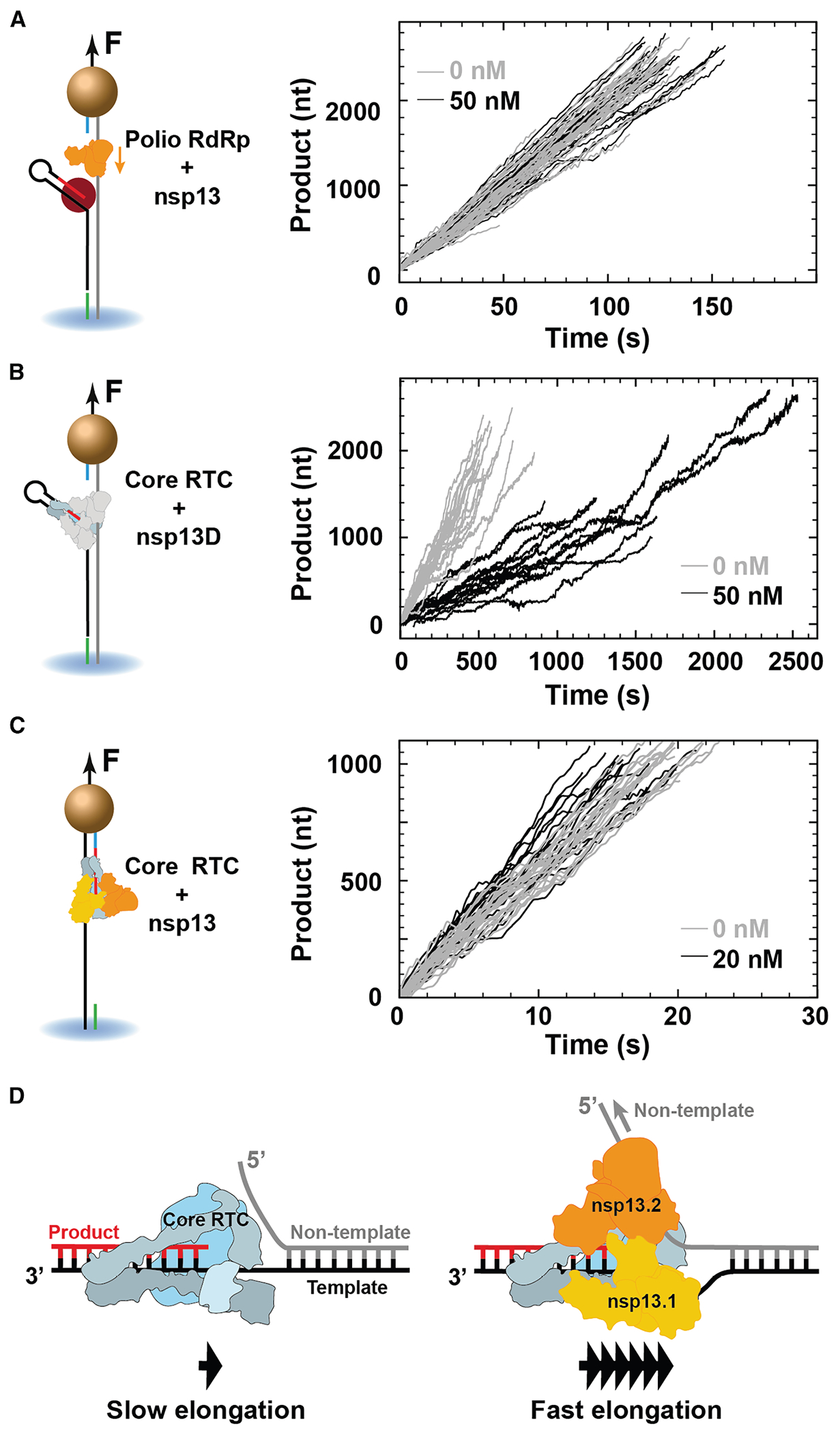
Nsp13 helicase forms a specific complex with the SARS-CoV-2 core RTC, and nsp13.2 supports the elongating polymerase by translocating on the non-template strand (A–C) On the left, schematics describing the measurements performed with nsp13 helicase and either poliovirus RdRp (A) or SARS-CoV-2 core RTC (B and C), elongating on either a dsRNA (A and B) or ssRNA (C) template, with either a wild-type nsp13 helicase (A and C) or an ATPase-dead K288A nsp13-helicase mutant (B). The corresponding elongation activity traces are shown on the right, i.e., without (gray) or with (black) nsp13 helicase at the indicated concentration. 20 pN was applied to the RNA during the measurements on a dsRNA template (A and B), while 25 pN was applied during the measurements on an ssRNA template (C). (D) Schematic representation of resuming the observations. The core RTC elongates slowly through duplex RNA. Once associated with the two helicases, nsp13.2 binds to the non-template RNA and utilizes ATP hydrolysis to translocate 5′ to 3′ and enable fast RTC elongation. See also Figures S3A–S3D.

To investigate whether nsp13 helicase associates specifically with the core RTC, thereby increasing the RNA synthesis rate, we replaced the core RTC with the PV RdRp and tracked RNA synthesis on the same dsRNA template. As PV RdRp has a different structure from the CoV core RTC,^8^ we expected only nonspecific interactions between PV RdRp and nsp13 helicase. Looking at PV RdRp elongation dynamics with and without nsp13 helicase, we observe no significant differences in the average elongation velocities: (18.7 ± 0.5) and (19.2 ± 0.7) nt/s, respectively (Figures 2A, S2A, and S2B). To further interrogate the specificity of nsp13-helicase association with the RTC, we monitored the core RTC elongation dynamics in the presence of the ATPase-dead mutant K288A nsp13 helicase (Figure 2B). A significant reduction of the RTC average elongation velocity relative to the core RTC alone was noticeable, from (3.6 ± 0.1) to (1.1 ± 0.1) nt/s.

We then investigated whether nsp13 helicase still affected the elongation dynamics of the core RTC. Previous single-molecule studies have shown that nsp13 helicase can unwind dsRNA with a 5′-to-3′ polarity.^13,14^ Using a hairpin construct where nsp13 helicase can only load and unwind in the 3′-to-5′ direction, we observed no hairpin unwinding, further supporting the exclusive 5′-to-3′ polarity of nsp13 helicase (Figures S3A and S3B). The difference in polarity between helicase and RTC suggests that head-to-head collisions may destabilize the RTC and/or slow down elongation. To test this hypothesis, we employed a ∼1-kb-long ssRNA template to monitor the RTC primer-extension dynamics at increasing concentrations of nsp13 helicase (Figures 2C, S2C, and S2D).^16,24^ Surprisingly, when comparing the cases without or with 20 nM nsp13 helicase, we observed a significant increase in product length ((778 ± 42) to (982 ± 16) nt) and an increase in the RTC average elongation velocity ((53.9 ± 1.3) to (61.6 ± 1.1) nt/s) when we looked at events faster than 5 s (Figure 2C). This indicates that nsp13 helicase allosterically increases the elongation rate after binding the core RTC. If collisions between translocating nsp13 helicase and RTC occurred, they did not significantly impact the elongation of the latter.

Cryo-EM studies found that either nsp13.1 or nsp13.1 and nsp13.2 are associated with the core RTC but never with nsp13.2 alone.^4,10^ This observation suggests a stepwise assembly process, where nsp13.1 binds the core RTC first, followed by nsp13.2. Furthermore, it was shown that nsp13.1 associates with the template RNA.^4,10^ Our data show rapid RNA synthesis on dsRNA when the RTC is associated with an ATPase-active nsp13 helicase (Figures 1C–1E). Using an ATPase-dead mutant instead resulted in slower elongation dynamics (Figure 2B). Altogether, we conclude that nsp13.2 accelerates RNA synthesis through duplex RNA by specifically associating with the RTC and translocating on the non-template strand (Figure 2D).

**Figure 3.**
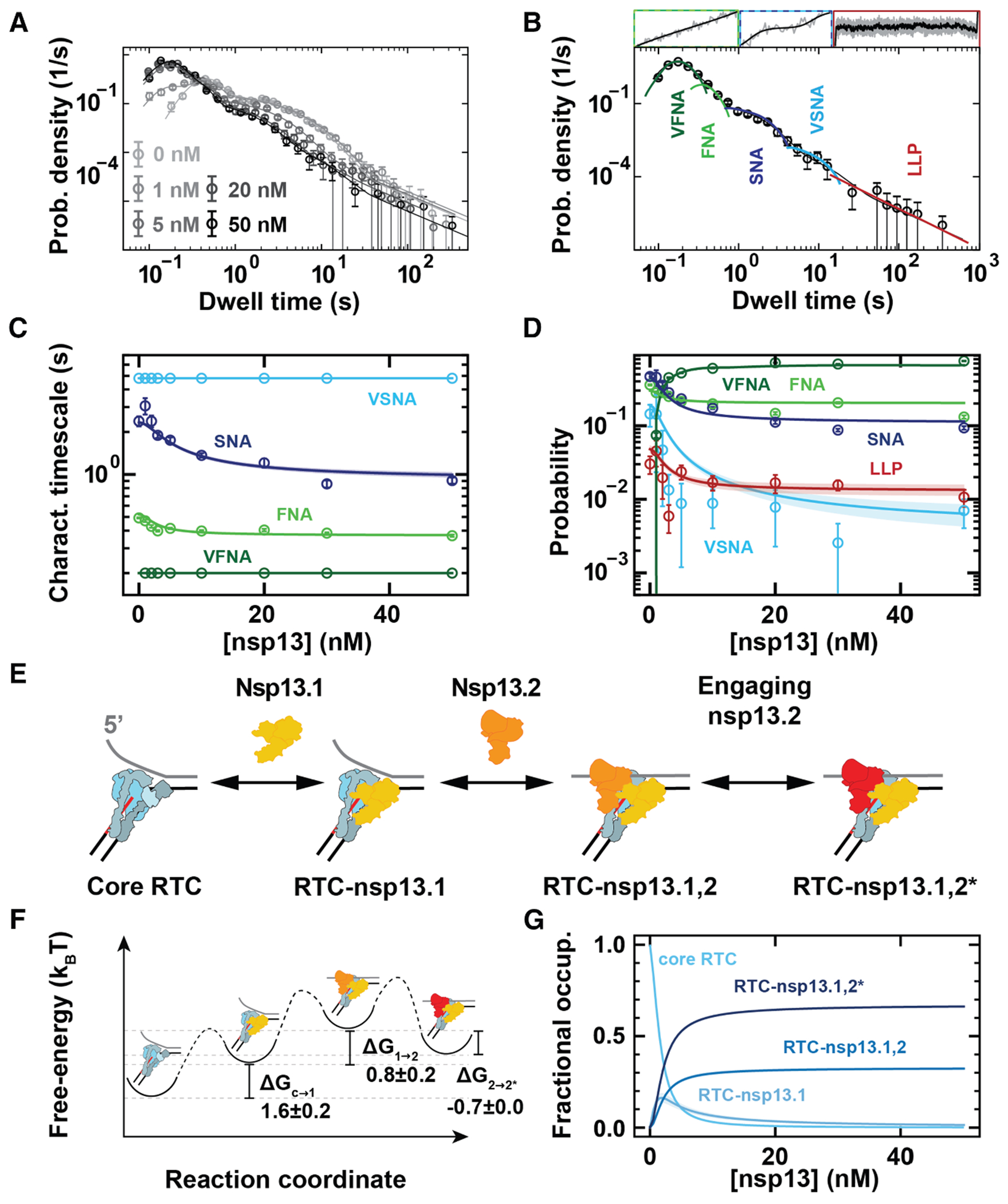
Assembly and stability of the SARS-CoV-2 polymerase-helicase complex (A) Dwell-time distributions extracted from RTC elongation traces on a dsRNA template with 20 pN tension, which were obtained at the indicated nsp13-helicase concentrations (circles) and their respective dwell-time fits (solid lines). (B) The dwell-time fit function consists of five probability density functions (PDFs): very fast, fast, slow, and very slow nucleotide addition (VFNA, FNA, SNA, and VSNA, respectively) pathways and long-lived pauses (LLPs). These PDFs are combined and fitted (black line) on the dwell-time distribution (circles). The insets at the top are snapshots of representative parts of the traces. (C and D) Characteristic timescales and probabilities of the different NA pathways and LLP state versus nsp13-helicase concentration. The circles with error bars in (A)–(D) and the solid lines with shaded areas in (C) and (D) represent the mean ± SEM of the individual fits and the RTC assembly-elongation dynamics model fits, respectively. VFNA and VSNA characteristic timescales were fixed to 0.2 and 4.8 s. (E) Schematic of the RTC assembly model. (F and G) The extracted free energy landscape (F) and corresponding fractional occupancies (G) versus nsp13-helicase concentration fitted on the dwell-time distributions with shaded areas as error bars, as in (C) and (D). See also Figures S2E, S2F, S2K, S2L, S2Q, S2R, S3E–S3G, and S5J–S5M.

**Figure 4.**
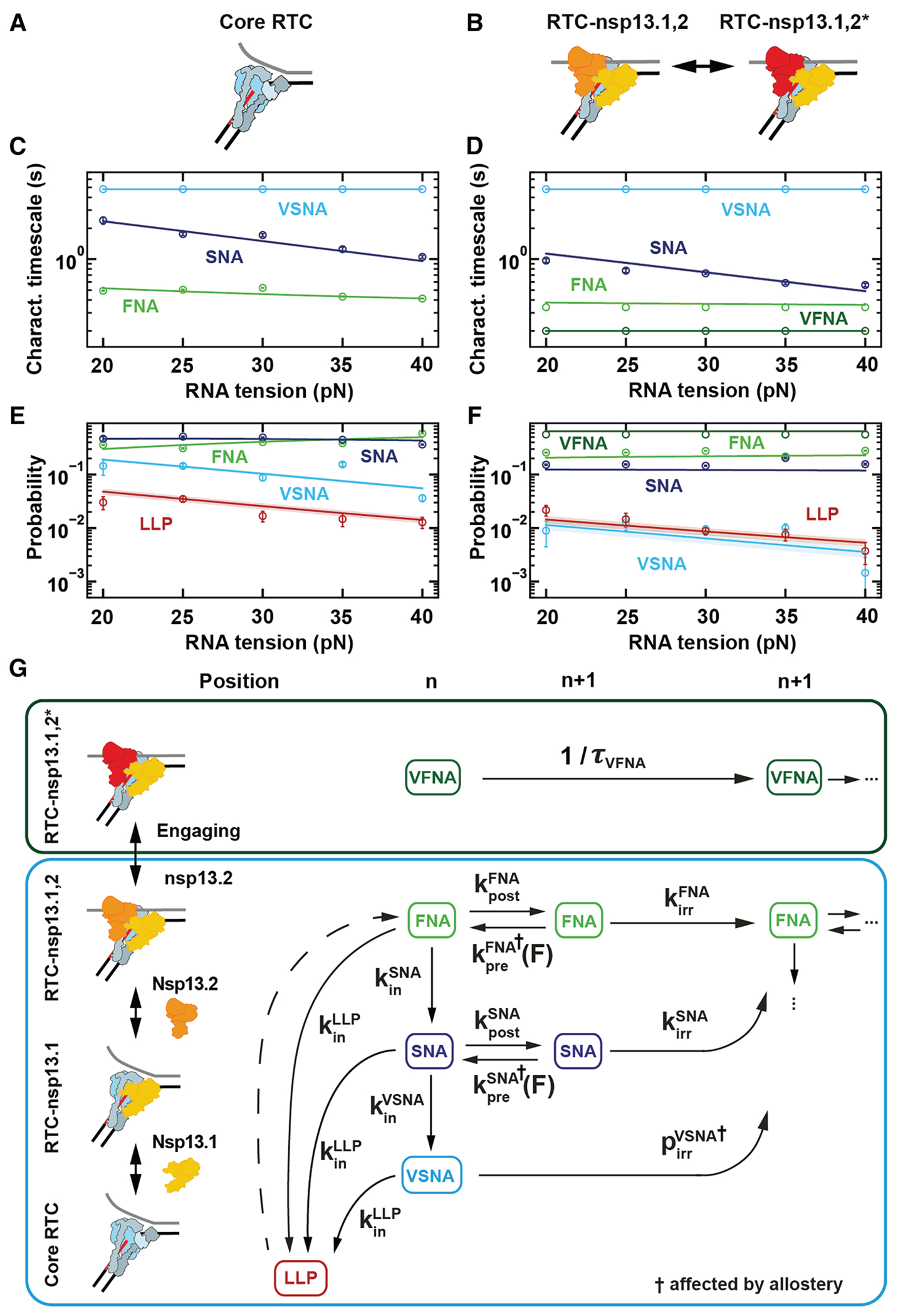
Mechanochemical reaction scheme for the nucleotide addition cycle of the SARS-CoV-2 core RTC alone or in complex with nsp13 helicase (A and B) Schematic representations of the different elongating RTCs without (A) and with (B) nsp13 helicase at saturating concentration. (C–F) Characteristic timescales (C and D) and probabilities (E and F) for the different nucleotide addition pathways (VFNA, FNA, SNA, and VSNA) and long-lived pause (LLP) state versus tension applied to the non-template RNA strand and without (C and E) or with (D and F) saturating nsp13-helicase concentration (20 nM). The circles with error bars represent the mean ± SEM from 100 bootstraps of the individual dwell-time fits. The solid lines and shaded areas represent the mean ± SEM of the global fit, with the mechanochemical reaction scheme shown in (G) (STAR Methods). (G) Mechanochemical reaction scheme underlying the timescales and probabilities (green box) for the VFNA pathway, which is only entered by the RTC-nsp13.1,2* complex, and (blue box) for the nucleotide addition pathways entered by the core RTC, RTC-nsp13.1, and RTC-nsp13.1,2. The solid and dashed arrows represent kinetic steps for which rates could either be extracted or not, respectively, from the global fit. See also Figures S2G–S2J, S2M–S2P, S4, and S5N–S5P.

In the following sections, we employ statistical analysis and kinetic modeling to establish the NA pathways and the energy landscape of the RTC-nsp13 assembly (Figure 3). In addition, our kinetic model captures how nsp13.2 binding increases RNA synthesis velocity (Figure 4).

### Nsp13 helicase modifies the kinetic fingerprint of the RTC elongation

We analyzed the RTC elongation dynamics on a dsRNA template at 20 pN tension as a function of nsp13-helicase concentration (Figures 1C–1E). We scanned the elongation traces with a non-overlapping window of 10 nt and measured the duration of 10 consecutive NA cycles, which we coined dwell times.^21,25^ We previously showed that there are four kinds of events in the core RTC elongation dynamics that can dominate the dwell-time window.^16^ The shortest dwell times (less than ∼1 s) for the core RTC are described by a gamma distribution representing the ten uninterrupted FNA events. Intermediate duration dwell times (from 1 to 20 s) are described by two exponential distributions, corresponding to the SNA and the very slow nucleotide addition (VSNA) pathways.^16^ A power-law distribution with a ∼*t*^−3/2^ decay describes dwell times longer than ∼20 s, which are referred to as LLPs (Figures 3A and 3B; STAR Methods). In the presence of nsp13 helicase, we observe VFNA bursts (∼0.2 s) in the traces (Figure 1D), resulting in a new gamma peak in the dwell-time distribution (Figures 3A and 3B).

The different distributions are combined into a single fit function, and the characteristic timescales (inverse rates) and probabilities (weights of the distributions) of the different pathways are extracted using maximum-likelihood estimation (MLE) (Figures 3A, 3B, S2, and S5; STAR Methods).^25^

### Connection of the RTC-nsp13 complex assembly model with RTC elongation dynamics

We now set out to discern the assembly of the RTC-nsp13 complex from the features captured in the traces and the dwell-time distributions obtained on dsRNA at a constant RNA tension of 20 pN. We noticed that the characteristic timescale of the VFNA cycles is independent of nsp13-helicase concentration (Figures 3D, S2E, and S2K) and that VFNA bursts always lasted tens to hundreds of NA cycles (Figure 1D). These observations suggest that single stable complexes are responsible for the VFNA bursts over multiple dwell-time windows.

The dwell-time distributions representing the RTC elongation dynamics on dsRNA do not change when increasing nsp13-helicase concentrations above 10 nM (Figure 3C). This indicates that the two nsp13-helicase binding sites on the RTC are occupied at these concentrations.^4,10^ However, above 10 nM nsp13 helicase, we still observe slow kinetic events in the traces (Figure 1C), consistent with entering the FNA, SNA, and VSNA pathways and the LLP state (Figure 3B). We conclude that a single RTC-nsp13 complex with two bound nsp13 helicases can exist in two configurations: one only entering the VFNA pathway and the other entering the slow kinetic events. Structural work has shown that the nsp13.2 conformation allosterically controls nsp13.1 engagement with the RNA template strand.^15^ We propose that the nsp13.1 conformation can similarly control nsp13.2 engagement with the non-template RNA and entry into the VFNA pathway. Based on these findings, we consider an RTC assembly model for several complexes and states: nsp13.1 in association with the core RTC (RTC-nsp13.1), nsp13.2 in association with the RTC-nsp13.1 and not engaged with the non-template strand (RTC-nsp13.1,2), and nsp13.2 in association with the RTC-nsp13.1 and engaged with the non-template strand (RTC-nsp13.1,2*). The latter configuration results in VFNA bursts (Figures 3E and S3E–S3G; STAR Methods).

Assuming that RTC-nsp13.1 shows the same elongation dynamics as the core RTC, we use the RTC assembly model to globally fit the dwell-time distributions at all measured nsp13-helicase concentrations (solid lines, Figures 3C, 3D, and S5J). From this fit, we extract the free energy differences between each RTC state (Figure 3F): (1.6 ± 0.2) *k*_B_*T* for core RTC to RTC-nsp13.1, (0.8 ± 0.2) *k*_B_*T* for RTC-nsp13.1 to RTC-nsp13.1,2, and (−0.7 ± 0.0) *k*_B_*T* for RTC-nsp13.1,2 to RTC-nsp13.1,2*. From these free energy differences, we obtained the equilibrium association constants *K*_a_ for the binding of nsp13.1 and nsp13.2 to the RTC, i.e., (0.22 ± 0.05) and (0.47 ± 0.08) nM^−1^, respectively. We also derived the fractional occupancy of each complex at 20 nM nsp13 helicase (saturating condition): (0.64 ± 0.01) for RTC-nsp13.1,2*, (0.31 ± 0.01) for RTC-nsp13.1,2, (0.034 ± 0.005) for RTC-nsp13.1, and (0.0081 ± 0.0007) for the core RTC (Figure 3G; STAR Methods).

Interestingly, the saturating nsp13-helicase concentration for two bound helicases to the core RTC (∼10 nM, Figure 3G) is much lower than the saturating nsp12-polymerase concentration for binding to the RNA (∼200 nM),^16,26^ indicating that nsp13 helicase binds the elongating core RTC with a much higher affinity than it binds freely diffusing nsp12 polymerase or core RTC. This likely originates from nsp13.1 and nsp13.2 forming interactions with the N-terminal poles of nsp8.2 and nsp8.1 (Figure 1A).^4,10^ Indeed, these poles are very flexible in the absence of duplex RNA,^8,27^ which may hinder nsp13-helicase association.

To probe the interactions of nsp13 helicase with nsp8 in the RTC without affecting the ATP hydrolysis cycle, we mutated the methionine 68, proline 78, tyrosine 253, and leucine 256 residues to alanine in nsp13, referred to as nsp13M4, which should weaken the association between nsp13.1 and nsp8b without abolishing it.^4^ Interestingly, these mutations are detrimental to viral replication *in cellulo*,^11^ supporting their relevance in viral RNA synthesis. We first determined whether the hairpin unwinding activity of nsp13M4 is affected by these mutations. We found similar average velocities and processivities for the wild-type nsp13 helicase and nsp13M4, demonstrating that these mutations do not affect the helicase activity of nsp13 (Figures S3C and S3D). We then monitored the RTC elongation dynamics in the presence of nsp13M4. The dwell-time distributions demonstrated the same signature trends as for the complex formed with wild-type nsp13 helicase, i.e., two peaks, two shoulders, and a power-law decay on long timescales (Figure S5Q). Furthermore, the change in VFNA probability with increasing nsp13M4 concentration could also be described by the same assembly model (Figure S3G). However, the mutations significantly reduced the fractional occupancy of RTC-nsp13.1,2* at saturating nsp13M4 concentration while keeping similar binding affinities (Figure S3G; Table S2). This result suggests that nsp13.1 and nsp13.2 interactions with the CoV polymerase are tightly coordinated, confirming a previous structural study,^15^ and that the fractional occupancy of RTC-nsp13.1,2* is an important parameter for a successful infection.

### The CoV RTC NA cycle

We now build a mechanochemical reaction scheme that captures the non-bursting elongation dynamics of the different RTCs and use the dependence on RNA tension to extract how the NA pathways and LLP state are connected. To this end, we consider that the polymerase NA cycle starts from the pre-translocated state and consists of several successive steps: translocation, NTP binding, catalysis, and pyrophosphate release (Figure S4A).^16^

We monitored the RTC elongation dynamics on a dsRNA template as a function of applied tension (20–40 pN), either for the core RTC alone (Figures 4A, 4C, and 4E) or at saturating nsp13-helicase concentration (20 nM) (Figures 4B, 4D, and 4F). The characteristic timescale of the VFNA bursts shows no significant trend with tension (Figures 4D and 4F), so we can only conclude that it is not rate limited by translocation. The characteristic timescales of the non-bursting NA pathways show a dependence on tension (Figures 4C–4F), allowing us to establish how they are connected (Figure 4G).

For a dsRNA template, the downstream dsRNA fork opposes RTC translocation as an energy barrier that is modulated by force.^19,21^ In our previous work, we showed that the back-and-forth translocation process is not equilibrated, while NTPs are at saturating concentrations and therefore the NTP-binding step is equilibrated.^16^ Furthermore, we found that only the backward translocation in the FNA pathway is affected by the force applied on the template (Figure S4A).^16^ Finally, we assume that all non-bursting NA pathways can be similarly modeled.

From the fits to the dwell-time distributions (circles, Figures 4C–4F), we see that the FNA characteristic timescale of the core RTC decreases with increasing RNA tension, confirming that the tension biases the RTC toward the FNA post-translocated state. Furthermore, the FNA probability increases with tension, while both the SNA and VSNA probabilities decrease (circles, Figure 4E). This tension dependence suggests that the slower NA pathways originate from the FNA pre-translocated state (Figure S4B), as we previously established for the core RTC.^16^ With increasing tension, the VSNA probability decreases with respect to the SNA probability, indicating that the VSNA is entered from the SNA pre-translocated state (Figures 4E and 4F). The ratio of the LLP and VSNA probability is constant with tension (Figures 4E and 4F), suggesting that the LLP state is also predominantly entered from the SNA pre-translocated state. However, the ratio of LLP to VSNA probability is different between the core RTC and RTC-nsp13.1,2 (Figures 4E and 4F). To allow the model to capture this shift, we assume the LLP can be entered from the pre-translocated state of each NA pathway (Figure 4G; STAR Methods).

In summary, we model the non-bursting NA pathways as starting from the pre-translocated state of the FNA pathway. From there, the RTC can directly incorporate a nucleotide, enter the SNA pre-translocated state, or enter the LLP. The RTC can then incorporate a nucleotide via the SNA pathway, enter the VSNA pre-translocated state and incorporate a nucleotide, or enter the LLP state. Once the LLP state is entered, we cannot determine from which NA pathway the RTC eventually exits, since the dwell time is dominated by the pause duration.

### Effect of nsp13.2 association on the RTC-nsp13.1,2 NA cycle

Interestingly, when the RTC elongates on a dsRNA template, we find that the FNA and SNA characteristic timescales significantly decrease with nsp13-helicase concentration, saturating above 10 nM (Figure 3C). The similar saturation for the VFNA pathway indicates that RTC-nsp13.1,2 elongation dynamics is also affected. On an ssRNA template, we find that the FNA characteristic timescale decreases significantly when nsp13 helicase is added (Figure S2C), indicating that the decrease in timescales is the result of an allosteric effect of nsp13 association. The decrease in FNA timescale is still apparent at saturating nsp13-helicase concentration, indicating that at least the association of nsp13.2 impacts the elongation velocity.

From these findings, we infer that the elongation dynamics of the core RTC and RTC-nsp13.1 can be assumed indistinguishable, while RTC-nsp13.1,2 employs the same pathways but with different timescales and probabilities due to the allostery of the nsp13.2 association. The VFNA pathway is only entered from the nsp13.2-engaged state (RTC-nsp13.1,2*). We confirm this model by connecting the RTC-nsp13 assembly with the RTC elongation dynamics and global fitting to the dwell-time distributions with increasing nsp13 concentration on the dsRNA template (Figures 3A and S5J–S5L; Table S3; STAR Methods).

Furthermore, from the global fits of the RTC assembly-elongation dynamics model to the dwell-time distributions for increasing nsp13M4 concentration (Figure S5R), we determined that the single-nucleotide timescales and probabilities of the NA pathways for the RTC-nsp13.1,2 state have similar values with nsp13M4 or wild-type nsp13 helicase in the reaction mixture (Table S2). From these fit results, we conclude that the allosteric effect of nsp13 helicase binding to the RTC is unchanged by the M4 mutation.

We now investigate how the allostery of the nsp13.2 association to the RTC can be described on the mechanochemical level. Increasing RNA tension has no significant effect on the FNA characteristic timescale and probability for RTC-nsp13.1,2 (Figures 4D and 4F). There is only an RNA tension dependency on the backward translocation rates, *k*_pre_^NA^(*F*), in our model for the FNA and SNA pathways (Figure 4G). Therefore, the force independence of the FNA characteristic timescale and probability can be captured by the FNA backward translocation rate, *k*_pre_^FNA^(*F*), becoming negligible compared to the effective nucleotide incorporation rate, *k*_irr_^FNA^, upon nsp13.2 association (Table 1). In contrast, the SNA characteristic timescale still decreases with tension for RTC-nsp13.1,2 (Figures 4C and 4D), indicating that SNA backward translocation with rate *k*_pre_^SNA^(*F*) is not completely abolished in the SNA pathway by nsp13.2 association. Furthermore, at the same tension, the ratio of VSNA to SNA probabilities is smaller for the core RTC than for the RTC-nsp13.1,2 (Figures 3D, 4E, and 4F). This effect is captured by a decreased probability for nucleotide incorporation in the VSNA pathway, *p*_irr_^VSNA^, for the RTC-nsp13.1,2 complex (Figure 4G; Table 1; STAR Methods).

From this model, we conclude that the allosteric effect of nsp13.2 association with the RTC makes backward translocation negligible in the FNA pathway. Our model suggests that the binding of nsp13.2 also reduces the backward translocation rate in the SNA pathway and decreases the probability for nucleotide incorporation in the VSNA pathway.

### A unified model captures all the data

Combining RTC assembly, elongation dynamics, and the mechanochemical reaction scheme (STAR Methods; Figure S1), we capture the complete dependence of the dwell-time distributions on the nsp13-helicase concentration and tension through a global fit (Figures 3C, 3D, 4C–4F, and S5N–S5P). Our ability to quantitatively capture all dependencies and distributions with a unified model lends confidence to our reaction scheme (Figure 4G) and allows us to extract its rates and the free energy differences controlling the RTC assembly (Table 1; STAR Methods).

## DISCUSSION

While nsp13 helicase has been proposed to support RNA synthesis,^11^ this function remains to be shown. Here, we employed high-throughput, single-molecule, magnetic tweezers to show that nsp13 helicase specifically associates with the elongating core RTC. Furthermore, nsp13.2 supports RTC elongation on a dsRNA template by translocating on the non-template strand (Figures 1 and 2). These results solve the conundrum of nsp13 helicase supporting nsp12-polymerase translocation while having opposite polarities. Our large statistics in many conditions enabled us to reveal the mechanism of assembly of the CoV RTC with nsp13 helicase through extensive statistical analysis and kinetic modeling. Namely, two nsp13 helicases associate sequentially to the core RTC, with nsp13.1 binding first, followed by nsp13.2 (Figures 2 and 3). Moreover, our results support a model where nsp13.2 alternates between a non-engaged and an engaged state with the non-template RNA. We showed that at saturating concentration of nsp13 helicase, ∼31% and ∼65% of the incorporated nucleotides, respectively, originate from an RTC associated with either a non-engaged or an engaged nsp13.2 (Figure 3). Finally, we derived a unified model describing both the RTC assembly and the NA mechanochemistry of the RTCs (Figure 4).

Our data provide unequivocal evidence that nsp13 helicase directly enhances the RTC by enabling efficient elongation through structured RNA. Cryo-EM tomography has revealed the presence of dsRNA within the replication organelles of infected cells,^28,29^ and duplex RNA has been suggested to serve as intermediates in both viral replication and transcription.^30^ The existence of such intermediates would substantiate a functional role for nsp13 helicase in supporting polymerase progression. However, even if dsRNA intermediates are not required during CoV replication, the highly structured nature of the viral genome^17^ alone would likely necessitate helicase assistance to sustain rapid and processive RTC elongation.

Nsp13.2 has been shown to allosterically control the productive engagement of nsp13.1 with the RNA template strand.^15^ Similarly, our data support a model where nsp13.1 allosterically controls nsp13.2 productive engagement with the non-template RNA. We propose that this mechanism ensures that only one helicase is actively engaged with its associated RNA strand at a time, preventing antagonistic activities of the two helicases. Our results suggest the VFNA pathway is only populated by RTC-nsp13.1,2* and that nsp13.2 must not be engaged with the non-template strand for the RTC to enter a LLP, including polymerase backtracking.^31^

The RTC bound with two nsp13 helicases stochastically alternates between the two elongation states RTC-nsp13.1,2 and RTC-nsp13.1,2*. This state switching provides a striking example of dynamic heterogeneity^32^ and may regulate nsp13.1 productive engagement with the template RNA. Nsp13.1 has been proposed to stimulate RTC backtracking, to promote either polymerase template switching^33^ or proofreading by the 3′-to-5′ exonuclease nsp14,^4^ though these functions remain to be demonstrated.^11^ This ability to stochastically switch between nsp13.1 and nsp13.2 productive engagement may enable a high level of regulation through sensing sequence-dependent context during replication. This could help to detect transcription regulatory sequences to regulate the fraction of complexes performing continuous versus discontinuous replication or to sense nucleotide mismatch incorporation to enable proofreading nsp14.

We anticipate that our results will impact our understanding of the mechanism of action of antiviral nucleotide analogs targeting the RTC, such as the FDA-approved remdesivir.^34^ Indeed, remdesivir incorporation by nsp12 polymerase has been shown to induce long-lived, polymerase-backtrack-related pauses during elongation.^24,35–38^ As nsp13.2 assists the RTC in elongating through dsRNA, it is tempting to think that nsp13 helicase may help the RTC overcome the pause induced by remdesivir incorporation. Whether the mechanism of action of antiviral nucleoside analogs targeting the CoV RTC should be revised when evaluated in the presence of additional viral co-factors associated with the core RTC (nsp13 helicase and nsp14 exonuclease) will require future investigation.

Our approach, combining high-throughput single-molecule biophysics and kinetic modeling, is uniquely capable of reconstituting an RNA-synthesis-competent extended CoV RTC, complementing our structural knowledge with function. This study paves the way toward the investigation of other (+)RNA virus RTCs and reveals whether the function discovered here for CoV nsp13 helicase is conserved for other viral helicases.

## Limitations of the study

In this study, there is no direct observation of nsp13-helicase binding dynamics to the core RTC, which limits our understanding of the respective stability of each helicase association. Due to limited spatiotemporal resolution, we cannot resolve single-nucleotide steps by the RTC in the elongation traces, and we therefore assumed that the NA pathways can be entered with the same probability and have the same characteristic timescale for every NA.

## RESOURCE AVAILABILITY

### Lead contact

Further information and requests for resources and reagents should be directed to and will be fulfilled by the lead contact, Dr. David Dulin (d.dulin@vu.nl).

### Materials availability

This study did not generate new unique reagents.

## STAR★METHODS

### EXPERIMENTAL MODEL AND STUDY PARTICIPANT DETAILS

#### Cell lines

All cell lines are purchased from commercial sources and used at low passage. Sf9 and Sf21 cell lines were routinely tested for mycoplasma contamination.

### METHOD DETAILS

#### Recombinant expression of SARS-CoV-2 nsp7, 8, and 12

This protocol was described by Seifert et al.^24^ The SARS-CoV-2 nsp12 gene was codon optimized and cloned into pFastBac with C-terminal additions of a TEV site and strep-tag (Genscript). The pFastBac plasmid and DH10Bac *E. coli* (Life Technologies) were used to create recombinant bacmids. The bacmid was transfected into Sf9 cells (Expression Systems) with Cellfectin II Reagent (Life Technologies) to generate recombinant baculoviruses. The baculoviruses were amplified through two passages in Sf9 cells, and then used to infect 1 L of Sf21 cells (Expression Systems), in which they were incubated for 48 h at 27°C. The cells were harvested by centrifugation, resuspended in wash buffer (25 mM HEPES pH 7.4, 300 mM NaCl, 1 mM MgCl_2_, 5 mM DTT) with 143 μL of BioLock (IBA LifeSciences) per liter of culture. The cells were lysed via microfluidization (Microfluidics). The lysates were cleared by centrifugation and filtration. The nsp12 protein was purified using Strep-Tactin Superflow agarose (IBA LifeSciences) and further purified by size exclusion chromatography using a Superdex 200 Increase 10/300 column (GE Life Sciences) in 25 mM HEPES, 300 mM NaCl, 100 μM MgCl_2_, 2 mM TCEP, at pH 7.4. The pure protein was concentrated by ultrafiltration prior to flash freezing in liquid nitrogen.

The SARS-CoV-2 nsp7 and nsp8 genes were codon optimized and cloned into pET46 (Novagen) with an N-terminal 6× histidine tag, an enterokinase site, and a TEV protease site. Rosetta2 pLys *E. coli* cells (Novagen) were used for the bacterial expression. The cultures were grown to an OD600 of 0.8 and induced with a final concentration of 0.5 mM isopropyl β-D-1-thiogalactopyranoside (IPTG) and the growth temperature was reduced to 16°C for 16 h. The cells were then harvested by centrifugation and the pellets were resuspended in wash buffer (10 mM Tris pH 8.0, 300 mM NaCl, 30 mM imidazole, 2 mM DTT). The cells were lysed via microfluidization and the lysates were cleared by centrifugation and filtration. The proteins were purified using Ni-NTA agarose beads (Qiagen) and eluted with wash buffer containing 300 mM imidazole. The eluted proteins were digested with 1% w/w TEV protease during overnight dialysis at room temperature (10 mM Tris pH 8.0, 300 mM NaCl, 2 mM DTT). After digestion, the proteins were passed back over Ni-NTA to remove undigested protein before concentrating the proteins by ultrafiltration. The nsp7 and nsp8 proteins were further purified by size exclusion chromatography using a Superdex 200 Increase 10/300 column (GE Life Sciences). The purified proteins were concentrated by ultrafiltration prior to flash freezing with liquid nitrogen.

#### Recombinant expression of nsp13 helicases

The coding sequence for nsp13-helicase from the SARS-CoV-2 Washington isolate (GenBank MN985325) was synthesized as an *E. coli* codon-optimized fragment (GenScript) and cloned into the *Bsa*I site of the pSUMO plasmid (LifeSensors) to produce an *N*-terminal six histidine-tagged SUMO-NSP13-HELICASE fusion cassette (6XHis-SUMO-nsp13). Using the manufacturer’s recommendations (QuikChange II Site-Directed Mutagenesis Kit, Agilent Technologies), oligonucleotide- and site-directed mutagenesis was used to mutate the codon in the wild-type nsp13-helicase for the critical catalytic lysine in the Walker A motif (K288) to an alanine (A) residue. This change produced the K288A mutant lacking ATPase activity (designated nsp13D). Alternatively, we mutated Met68A, Pro78A, Tyr253A and Leu256A, referred to as nsp13M4. The final wild-type and mutant plasmids were sequence-verified using a 3130XL Genetic Analyzer (Applied Biosystems). The SUMO-nsp13, SUMO-nsp13D and SUMO-nsp13M4 constructs were transformed into Rosetta2 cells (Novagen), and colonies were grown overnight at 37°C in NZCYM (Research Products International) supplemented with kanamycin (50 μg/mL) and chloramphenicol (25 μg/mL). The cultures were diluted 1:100 into fresh antibiotic-containing NZCYM media and grown to an OD_600_ nmof 0.8–1. The bacterial media were supplemented with 0.1 mM ZnSO_4_ and 0.2% dextrose and cooled on ice for 10 min. Wildtype and mutant protein expression was induced with 0.2 mM isopropyl β-D-1-thiogalactopyranoside at 18°C for 12–16 h. The cells were harvested by centrifugation at 4,000 × *g* for 15 min at 4°C and the pellets were stored at −80°C. All purifications steps were carried out on ice or at 4°C. The pellets were resuspended in lysis buffer (50 mM sodium phosphate, pH 8.0, 300 mM NaCl, 1 mM β-mercaptoethanol, 10% glycerol and 20 mM imidazole) supplemented with 2 mM phenylmethylsulfonyl fluoride (PMSF) and 1X EDTA-free protease inhibitor (Pierce). The bacteria were lysed by microfluidization and the lysate clarified by centrifugation at 17,000 × *g* for 1 h at 4°C. The His-tagged SUMO-nsp13 was passed through a HisTrap FF column (Cytiva) equilibrated in lysis buffer at 1 mL/min using an Akta FPLC (Cytiva). The affinity resin was washed with 20 column volumes of lysis buffer, and the protein eluted with 10 column volumes of lysis buffer containing 200 mM imidazole. The pooled SUMO-nsp13-containing fractions was dialyzed overnight into two changes of 20 mM imidazole-containing lysis buffer, and the SUMO tag cleaved with Ulp-1 for 4 h at 4°C. Digestion was confirmed by SDS-PAGE analysis. The His6-Ulp-1 and His6-SUMO proteins were separated from the native nsp13 and nsp13D with a second round of Ni^2+^-affinity chromatography as before. The helicase-containing flow-through fractions were pooled, dialyzed overnight against two changes of low salt buffer (50 mM sodium phosphate, pH 6.8, 150 mM NaCl, 4 mM β-mercaptoethanol, 0.5 mM EDTA and 10% glycerol) and passed through a HighTrap SP cation exchange column (Cytiva). Under these conditions, neither nsp13-helicase proteins adhered to the SP column. The proteins were concentrated with an Amicon Ultra-15 centrifugation filter units to a volume of ∼1.5 mL and loaded on to a Sephacyl S200-HR HiPrep 26/60 column (Cytiva) equilibrated with nsp13-helicase Storage Buffer (25 mM HEPES, pH 7.5, 150 mM NaCl, 0.5 mM TCEP and 20% glycerol). The final nsp13-helicase proteins were quantified by UV spectrophotometry at 280 nm using the expected extinction coefficient of 68,785 M^−1^ cm^−1^ and confirmed using the BCA Protein Assay (Pierce). Protein samples were aliquoted, flash frozen, and stored at −80°C.

#### Recombinant expression of poliovirus RdRp

Poliovirus RdRp was expressed and purified as previously reported.^43,44^ Briefly, expression was performed at 25°C by auto-induction. The cells were harvested, lysed by microfluidization, subjected to Polyethyleneimine (PEI) precipitation followed by Ammonium Sulfate precipitation, Ni-NTA chromatography, cleavage by Ulp1, phosphocellulose chromatography, gel filtration. The proteins were concentrated using Vivaspin concentrators.

#### Fabrication of dsRNA template

The RNA construct used here has been previously described in detail by Seifert et al.^18^ In brief, a 4 kb long single-stranded splint to which four ssRNA oligos are annealed (Table S1A): a biotin-labeled strand to attach to the streptavidin-coated magnetic bead (M-270), a spacer, ~2.9 kb template, and a digoxygenin-labeled strand to attach to the glass surface. The template strand ends in 3’-end with a small hairpin with the sequence ACGCUUUCGCGT followed by 15 U residues to initiate poliovirus RdRp catalyzed RNA synthesis via primer extension.

#### Fabrication of RNA hairpin with 3′-end loading site

The fabrication of the RNA hairpins has been described in detail by Papini et al.^40^ In brief, the RNA hairpin with a 3′-end ssRNA loading site (Table S1B) is made of a 499 bp double-stranded RNA stem terminated by a 20 nt loop that is assembled from three ssRNA oligos annealed together, and two handles, one of 856 bp at the 5′-end and one of 822 bp at the 3′-end. The handles include a 343 nt digoxygenin-labeled ssRNA or a 404 nt biotin-labeled ssRNA on either side to attach to the anti-digoxigenin coated glass surface and the streptavidin-coated magnetic bead (M-270), respectively.

#### Fabrication of RNA hairpin with 5′-end loading site

The RNA hairpin with a 5′-end ssRNA loading site (Table S1C) is made of a 499 bp double-stranded RNA stem terminated by a 4 nt loop that is assembled from three ssRNA oligos annealed together, and two handles, one of 836 bp at the 5′-end and one of 842 bp at the 3′-end. The handles include a 343 nt digoxygenin-labeled ssRNA or a 404 nt biotin-labeled ssRNA on either side to attach to the anti-digoxigenin coated glass surface and the streptavidin-coated magnetic bead (M-270), respectively.

#### High-throughput magnetic tweezers apparatus

The high-throughput magnetic tweezers used in this study have already been described in detail in our previous work.^22,23^ Briefly, two vertically aligned permanent magnets (5 mm cubes, SuperMagnete) separated by a 1 mm gap are positioned above the flow-cell (see paragraph below) which is mounted on a custom-built inverted microscope. The vertical position and rotation of the magnets are controlled by two linear motors (Physik Instrumente PI). The field of view is illuminated through the magnets gap by a collimated LED light source and is imaged onto a large chip CMOS camera (Teledyne Dalsa Falcon2) using a 50× oil immersion objective (NA 0.9, Nikon) and an achromatic doublet tube lens of 200 mm focal length and 50 mm diameter (Qioptic). The magnetic beads were imaged in the back-focal plane of the tube lens at an acquisition rate of 58 Hz. The real-time tracking of the magnetic bead x, y, z-positions was done on a GPU (NVIDIA Geforce 690 card) using a look-up table approach implemented in C++ CUDA as described in detail by Cnossen et al.^42^ To control the temperature, we used a system described in detail by Seifert et al.^18^ A flexible resistive foil heater with an integrated 10 MΩ thermistor (Thorlabs) is wrapped around the microscope objective and further insulated by several layers of Kapton tape (Thorlabs). The heating foil is connected to a PID temperature controller (Thorlabs) to adjust the temperature within ~0.1°C.

#### Force calibration of the magnetic tweezers

The forces applied on the RNA-tethered magnetic beads per magnet height were calibrated as described in detail by Ostrofet et al.^22^ and Quack and Dulin.^23^ Shortly, we employed the equipartition theorem that connects the transversal fluctuations and tether length to the applied force to calibrate the latter for varying distance of the magnets to the magnetic bead.

#### Flow-cell assembly

The fabrication procedure for flow cells has been described in detail by Quack et al.^23^ To summarize, we sandwiched a double layer of Parafilm by two #1 coverslips (Menzel), the top one has a hole at each end serving as inlet and outlet, the bottom one being coated with a 0.1% m/V nitrocellulose dissolved in amyl acetate solution. The flow cell was mounted into a custom-built holder and rinsed with ~1 mL of 1× phosphate buffered saline (PBS). 3 μm diameter polystyrene reference beads were attached to the bottom cover-slip surface by incubating 100 μL of a 1:1000 dilution in PBS (LB30, Sigma Aldrich, stock conc.: 1.828*1011 particles per milliliter) for ~3 min. After rinsing the flow cell with 0.5 mL of PBS, 50 μL of anti-digoxigenin (50 μg/mL in PBS) was incubated for 30 min in the flow cell. The flow cell was then flushed with 1 mL of high salt TE buffer (10 mM Tris, 1 mM EDTA pH 8.0, 750 mM NaCl, 2 mM sodium azide) to remove excess of anti-digoxigenin followed by rinsing with another 0.5 mL of low salt TE buffer (10 mM Tris, 1 mM EDTA pH 8.0, 150 mM NaCl, 2 mM sodium azide). The surface was then passivated with a solution of bovine serum albumin (BSA, 10 mg/mL in PBS and 5% glycerol) for 30 min, and rinsed with 0.5 mL of low salt TE buffer.

#### SARS-CoV-2 RTC activity assays on dsRNA template

20 μL of streptavidin-coated magnetic beads (Dynabeads M-270, ThermoFisher) was mixed with ~0.1 ng of RNA (total volume 40 μL) and incubated for ~5 min before rinsing with ~2 mL of TE buffer to remove any unbound RNA and the magnetic beads in excess. RNA tethers were sorted by looking for the characteristic extension of the correct length (~1 μm at 40 pN) due to the stretching of the dsRNA during a force ramp experiment.^40^ The flow cell was subsequently rinsed with 0.5 mL reaction buffer (50 mM HEPES pH 7.9, 10 mM DTT, 2 μM EDTA, 5 mM MgCl_2_). After starting the data acquisition at the indicated force, 100 μL of reaction buffer containing 0.2 μM nsp12, 1.8 μM nsp7, 1.8 μM nsp8 (1:9:9 stoichiometry), the indicated concentration of nsp13-helicase (WT or mutant) and 1 mM NTP were flushed in the flow cell to start the reaction. For the pre-assembled RTC experiments, the nsp’s were incubated for five minutes in the flow cell, while applying 25 pN force on the tether. The excess RTC proteins were subsequently flushed away with 0.3 mL of reaction buffer (flow cell volume ~40 μL), followed by the injection of 100 μL reaction buffer with the desired nsp’s and 1 mM NTP. The experiments were conducted at a constant force for a duration of 30–60 min. The camera frame rate and the temperature were respectively set to 58 Hz and 25°C. A custom written LabVIEW routine controlled the data acquisition and the (x-, y-, z-) positions tracking of both the magnetic and reference beads in real-time.^42^ Mechanical drift correction was performed by subtracting the reference bead position from the magnetic bead positions and further corrected using an autofocus (i.e., along the z axis) protocol previously described by Bera et al.^16^

#### Poliovirus RdRp activity assays on dsRNA template

20 μL of streptavidin-coated magnetic beads (Dynabeads M-270, ThermoFisher) was mixed with ~0.1 ng of RNA (total volume 40 μL) and incubated for ~5 min before rinsing with ~2 mL of TE buffer to remove any unbound RNA and the magnetic beads in excess. RNA tethers were sorted for functional dsRNA by looking for its characteristic contour length, i.e., ~1 μm. The flow cell was subsequently rinsed with 0.5 mL of reaction buffer (50 mM HEPES pH 7.9, 10 mM DTT, 2 μM EDTA, 5 mM MgCl_2_). After starting the data acquisition at a suitable force, 100 μL of reaction buffer containing 0.5 μM poliovirus RdRp, the indicated concentration of nsp13-helicase and 1 mM NTP were flushed in the flow cell to start the reaction. The experiments were conducted at a constant force for a duration of 30–60 min.

#### SARS-CoV-2 RTC activity assays on ssRNA template

20 μL of streptavidin-coated magnetic beads (Dynabeads M-270, ThermoFisher) was mixed with ~0.1 ng of RNA hairpins (with 3′-end ssRNA loading site, a total volume of 40 μL) and incubated for ~5 min before rinsing with ~2 mL of TE buffer to remove any unbound RNA and the magnetic beads in excess. RNA tethers were sorted for functional hairpins by looking for the characteristic jump in extension of the correct length (~0.6 μm at 30 pN) due to the sudden opening of the hairpin during a force ramp experiment.^40^ The flow cell was subsequently rinsed with 0.5 mL reaction buffer (50 mM HEPES pH 7.9, 10 mM DTT, 2 μM EDTA, and 5 mM MgCl_2_). After starting the data acquisition at a force (~25 pN) that would keep the hairpin open, 100 μL of reaction buffer containing 0.6 μM nsp12, 1.8 μM nsp7 and nsp8 and 20 nM nsp13-helicase with 500 μM NTP was flushed into the flow cell to start the reaction. The experiments were conducted at a constant force for a duration of 30–40 min. The camera frame rate was fixed at 58 Hz and the temperature was set to 25°C. A custom written LabVIEW routine controlled the data acquisition and the (x-, y-, z-) positions analysis/tracking of both the magnetic and reference beads in real time. Mechanical drift correction was performed by subtracting the reference bead position to the magnetic bead position and by applying an autofocus as described by Bera et al.^16^

#### Data processing

The activity traces were first corrected from the mechanical drift by subtracting the reference bead position to the tethers position, then converted from micron to synthesized nucleotides using the difference in extension for dsRNA and ssRNA under the same tension as described below. The traces were subsequently low-pass filtered with a Kaiser-Bessel window at 0.5 or 2 Hz for ds- or ssRNA template respectively and the dwell-times were extracted using a dwell-time window of 10 nt, as previously described.^21^ The dwell-times of all the traces for a given experimental condition were collected in a single distribution and further analyzed using a maximum likelihood estimation (MLE) fitting routine to extract the parameters from the dwell-time fit-function (Equation 7).

#### Number of synthesized nucleotides on dsRNA template

To calculate the number of synthesized nucleotides, i.e., product length, on the dsRNA template, we use the change in magnetic bead height from the conversion of double-stranded (ds) to single-stranded (ss) RNA tether under constant tension by an elongating RTC (Figure 1B). We performed an interpolation of the force-extension curves for the full dsRNA and ssRNA before and after complete RNA synthesis to calculate the number of nucleotides synthesized *N*_nt_(*F*) (product length) at tension *F* using the formula^21^

(Equation 1) $$N_{\mathrm{nt}}(F)=N\frac{L_{\mathrm{meas}}(F)-L_{\mathrm{ds}}(F)}{L_{\mathrm{ss}}(F)-L_{\mathrm{ds}}(F)}$$

Where *N* is the total number of nucleotides on the RNA template, *L*_meas_(*F*) is the extension of the molecule measured during RNA synthesis by the RTC at tension *F*, *L*_ds_(*F*) is the extension of the fully double-stranded RNA at tension *F* and *L*_ss_(*F*) the extension of the fully single-stranded RNA at tension *F*.

#### Number of synthesized nucleotides on ssRNA template

Similarly, the number of synthesized nucleotides *N*_nt_ (product length) on the ssRNA template were calculated from the difference in extension between the synthesized product at a specific timepoint *L*_meas_ and the ssRNA template *L*_ss_, the difference in extension between fully ssRNA *L*_ss_ and dsRNA *L*_ds_ at the same tension *F* and the total number of nucleotides on the ssRNA template *N*

(Equation 2) $$N_{\mathrm{nt}}(F)=N\frac{L_{\mathrm{ss}}(F)-L_{\mathrm{meas}}(F)}{L_{\mathrm{ss}}(F)-L_{\mathrm{ds}}(F)}$$

#### Number of unwound base pairs on an RNA hairpin

For the unwinding of RNA hairpins by nsp13-helicase (Figures S3A–S3D), we calculated the number of unwound base pairs *N*_bp_(*F*) from the measured extension of the molecule at the applied tension *L*_meas_(*F*), the extension of closed hairpins in the measurement *L*_hp closed_(*F*), the extension of the open hairpin at the same tension *L*_hp open_(*F*) and the total number of base pairs on the hairpin *N*

(Equation 3) $$N_{\mathrm{bp}}(F)=N\frac{L_{\text{meas}}(F)}{L_{\text{hp open}}(F)-L_{\text{hp closed}}(F)}$$

This is possible in the force regime with hysteresis, where the hairpin is stably closed when the tension is increased to *F* and the hairpin is stably open when the tension is decreased to *F*, i.e., 15–20 pN. For the same reason, the measurements of unwinding activity by nsp13-helicase are also performed in this force regime.

#### Total product length and average velocity

The total product length is defined as the farthest point on the template reached by the RTC complex, obtained from the extension of the RNA template over time using the conversion relation Equations 1 or 2 for a ds- or ssRNA template respectively. The average velocity of elongation by the RTC complexes is calculated as the total product length divided by the replication time, i.e., the time from the start of the activity to the timepoint at which the total product length is reached.

For dsRNA unwinding by nsp13-helicase alone, the processivity is defined as the maximum number of unwound base pairs (Equation 3) and the average velocity is obtained as the processivity divided by the time from the start of the activity to the timepoint at which the maximum number of unwound base pairs is reached.

#### Maximum likelihood estimation fitting routine

The dwell-time distributions were fitted to the experimentally collected dwell-times {*t_i_*} by maximizing the log likelihood function

(Equation 4) $$\mathrm{LL}=\underset{i}{\sum }\text{ln}P_{N\text{nt}}\left(t_{i}\right)$$

Where *P_N_*_nt_ represents the probability of every dwell-time *t_i_* in the empirical distribution based on the dwell-time fit-function, We calculated the statistical error on the parameters by applying the MLE fitting procedure on 100 bootstraps of the original dataset, and reported the standard deviation for each fitting parameter.

To be able to compare fits of models with different number of parameters, we calculated the Bayesian Information Criterion (BIC)

(Equation 5) BIC=kln⁡(N)-2ln⁡(LL)

This criterion compares the log likelihood *LL* to the number of parameters in the model *k* and the number of datapoints fitted *N* to account for the fact that using more parameters can lead to overfitting. The model fit with minimized BIC is considered the best fit with optimal number of parameters for the set of model fits tested.^45^

#### Dwell-time fit-function for RNA synthesis by RdRp

The dwell-time distributions of the SARS-CoV-2 RTC (or poliovirus RdRp) in absence of assistance by active nsp13-helicase were fitted with a dwell-time fit-function consisting of one gamma distribution with characteristic timescale *T*_FNA_ fitting the peak at short timescale, two exponential distributions with characteristic timescales *T*_SNA_ and *T*_VSNA_ fitting two shoulders in the dwell-time distributions and a power law distribution of ~ *t*^−3/2^ fitting the fat-tail in the dwell-time distributions for longer timescales^31^

(Equation 6) $$P_{N\text{nt}}(t)\approx \frac{f_{\text{FNA}}N}{T_{\text{FNA}}(N-1)!}{\left(\frac{\mathrm{tN}}{T_{\text{FNA}}}\right)}^{N-1}e^{-\frac{\mathrm{tN}}{T_{\text{FNA}}}}+Q(t)\left(\frac{f_{\text{SNA}}}{T_{\text{SNA}}}e^{-\frac{t-T_{\text{FNA}}}{T_{\text{SNA}}}}+\frac{f_{\text{VSNA}}}{T_{\text{VSNA}}}e^{-\frac{t-T_{\text{FNA}}}{T_{\text{VSNA}}}}+\frac{f_{\text{LLP}}\sqrt{1+T_{\text{FNA}}}}{2{\left(1+\frac{t}{1s}\right)}^{\frac{3}{2}}}\right).$$

The approximation of the SNA, VSNA and LLP dominated terms break down for the short timescales since *N* sequential steps always need to be taken to get through the dwell-time window. This is accounted for by regularization function $Q(t)=\frac{{\left(t/T_{\text{FNA}}\right)}^{N-1}}{1+{\left(t/T_{\text{FNA}}\right)}^{N-1}}$ and normalization of the terms starting from the peak position of the gamma distribution $T_{\text{FNA.}}\sum_{j}f_{j}=1$ for *j* ∈ {FNA, SNA, VSNA, LLP} ensured the distribution $P_{N\mathrm{nt}}(t)$ is normalized.

Considering that we observed a clearly separated peak and two shoulders in the dwell-time distributions, we distinguished three characteristic timescales in the dwell-time distribution dominated by fast, slow and very slow nucleotide addition and thus assumed clear separation of timescales for single nucleotide additions $\tau_{\text{FNA}}\ll \tau_{\text{SNA}}\ll \tau_{\text{VSNA}}$. With this assumption, we derived relations for the characteristic timescales and probabilities on the dwell-time level in terms of single nucleotide timescales and probabilities as described by Bera et al.^16^

For the power law distribution representing a long-lived pause, we have introduced a regularization at 1 s, but the precise timescale does not matter here, as long as it is set within the region dominated by either of the FNA, SNA or VSNA pathways. Due to this approximation, the long-lived pause probability *f*_LLP_ should be interpreted as the relative probability to enter the long-lived pause. For a more elaborate description, see STAR Methods section “dwell-time fit-functions for RNA synthesis”.

#### Dwell-time fit-function RNA synthesis with nsp13

The dwell-time distributions for elongation dynamics by the SARS-CoV-2 RTC with active nsp13-helicase were fitted with a dwell-time fit-function consisting of two gamma distributions with characteristic timescales *T*_VFNA_ and *T*_FNA_ fitting the two peaks at short timescale, two exponential distributions with timescales *T*_SNA_ and *T*_VSNA_ fitting two shoulders in the dwell-time distributions and a power law distribution ~ *t*^−3/2^ fitting the fat-tail in the dwell-time distributions for longer timescales (Figure 3B).

The dwell-time fit-function for the dwell-time distributions of RTC elongation dynamics on a dsRNA template in presence of active nsp13-helicase reads

(Equation 7) $$P_{N\text{nt}}(t)\approx \frac{f_{\text{VFNA}}N}{T_{\text{VFNA}}(N-1)!}{\left(\mathrm{tN}/T_{\text{VFNA}}\right)}^{N-1}e^{-\mathrm{tN}/T_{\text{VNA}}}+\frac{f_{\text{FNA}}N}{T_{\text{FNA}}(N-1)!}{\left(\mathrm{tN}/T_{\text{FNA}}\right)}^{N-1}e^{-\mathrm{tN}/T_{\text{FNA}}}\;+Q(t)\left(\frac{f_{\text{SNA}}}{T_{\text{SNA}}}e^{-\frac{t-T_{\text{FNA}}}{T_{\text{SNA}}}}+\frac{f_{\text{VSNA}}}{T_{\text{VSNA}}}e^{-\frac{t-T_{\text{FNA}}}{T_{\text{VSNA}}}}+\frac{f_{\text{LLP}}\sqrt{1+T_{\text{FNA}}}}{2{\left(1+t/1s\right)}^{\frac{3}{2}}}\right),$$

Where the regularization function $Q(t)=\frac{{\left(t/T_{\mathrm{FNA}}\right)}^{N-1}}{1+{\left(t/T_{\mathrm{FNA}}\right)}^{N-1}}$ was used and the SNA, VSNA and LLP dominated terms were normalized starting from the peak position of the gamma distribution $T_{\text{FNA}}$, like in the dwell-time fit-function for the core RTC. $\sum_{j}f_{j}=1$ for *j* ∈ {VFNA, FNA, SNA, VSNA, LLP} ensured the distribution $P_{N\text{nt}}(t)$ is normalized. Considering two peaks and two shoulders could be clearly distinguished in the empirical dwell-time distributions, we assumed clear separation of single nucleotide timescales $\tau_{\text{VFNA}}\ll \tau_{\text{FNA}}\ll \tau_{\text{SNA}}\ll \tau_{\text{VSNA}}$. The power law distribution has a regularization at 1 s, since the underlying kinetics are not expected to change in presence of active nsp13-helicase. Due to this approximation, the long-lived pause probability *f*_LLP_ should be interpreted as the relative probability to enter a long-lived pause. For a more elaborate description see STAR Methods section “dwell-time fit-functions for RNA synthesis”.

No significant trends were observed in the VFNA and VSNA characteristic timescales for the conditions measured, so they were fixed to *T*_VFNA_ = 0.2 s and *T*_VSNA_ = 4.8 s respectively (Figure S2).

#### Assembly of RTC-nsp13 complexes on a dsRNA template

We constructed an RTC-nsp13 assembly model including four RTC complexes, i.e., the RTC without nsp13-helicase (core RTC), the RTC with nsp13.1 bound (RTC-nsp13.1), the RTC with the two helicases bound and nsp13.2 not engaged (RTC-nsp13.1,2), the RTC with the two nsp13-helicases bound and nsp13.2 engaged with the non-template RNA (RTC-nsp13.1,2*) (Figure 3B).

We observed very fast nucleotide addition bursts spanning tens to hundreds of nucleotides already for low nsp13-helicase concentration on a dsRNA template (Figure 1C), meaning that a single RTC-nsp13.1,2* complex is stable for much longer than the timescale for synthesizing 10 nt RNA (dwell-time window). To discriminate between assembly models, we assumed the binding of two nsp13-helicases to the RTC is in dynamic equilibrium, but they do not exchange within one dwell-time window. Under this assumption, we can write the fractional occupancies *p*_cpx_ of the complexes cpx ∈ {c, 1, 2, 2*} = {core RTC; RTC-nsp13.1; RTC-nsp13.1,2; RTC-nsp13.1,2*} in terms of the free-energy barriers between the complexes $\Delta G_{\mathrm{cpx}\to {\mathrm{cpx}}^{\prime }}$

(Equation 8) $$p_{2^{\text{*}}}=\frac{{\left[\text{nsp}13\right]}^{2}e^{-\left(\text{Δ}G_{c\to 1}+\text{Δ}G_{1\to 2}+\text{Δ}G_{2\to 2^{\text{*}}}\right)}}{Z},\;p_{2}=\frac{{\left[\text{nsp}13\right]}^{2}e^{-\left(\text{Δ}G_{c\to 1}+\text{Δ}G_{1\to 2}\right)}}{Z},\;p_{1}=[\text{nsp}13]e^{-\text{Δ}G_{c\to 1}}/Z,\;p_{c}=1/Z,$$

With $Z=[\mathrm{nsp}13{]}^{2}e^{-\left(\Delta G_{c\to 1}+\Delta G_{1\to 2}+\Delta G_{2\to 2^{*}}\right)}+[\mathrm{nsp}13{]}^{2}e^{-\left(\Delta G_{c\to 1}+\Delta G_{1\to 2}\right)}+[\mathrm{nsp}13]e^{-\Delta G_{c\to 1}}+1$. The free-energy barriers between the complexes $\Delta G_{\mathrm{cpx}\to {\mathrm{cpx}}^{\prime }}$ are expressed in units of k_B_T with 1 nM as reference concentration. Here we used that the fraction of product from every single equilibrium reaction is given by a Boltzmann factor dependent on the nsp13-helicase concentration and the free-energy barrier.^46^

Furthermore, the association constants for nsp13.1 and nsp13.2 to the RTC were obtained from the free-energy barriers in the assembly model as

(Equation 9) $$K_{\mathrm{a,nsp}13.1}=e^{-\Delta G_{c\to 1}},\;K_{\mathrm{a,nsp}13.2}=e^{-\Delta G_{1\to 2}}.$$

Since we used 1 nM as reference concentration, the association constants are giving in the unit nM^−1^.

#### Connecting RTC-nsp13 assembly and elongation

We then built the RTC Assembly-Elongation dynamics model connecting the RTC assembly model with the elongation dynamics on a dsRNA template (dwell-time fit-function) for a mix of core RTC’s and RTC-nsp13 complexes with a constant tension on the non-template RNA strand.

We assumed that the RTC resides exclusively in one of the RTC-nsp13 states within one dwell-time window. Under this assumption, the first passage time distribution for elongation dynamics is obtained by summing the first passage time distributions $P_{10\mathrm{nt}}(t|\mathrm{cpx})$ for the different RTC complexes $\mathrm{cpx}\in \left\{\text{c},1,2,2^{*}\right\}$ with the fractional occupancies for each RTC complex $p_{\mathrm{cpx}}$ as pre-factor

(Equation 10) $$P_{N\text{nt,RTC}-\text{nsp}13}(t)=\underset{\text{cpx}}{\sum }p_{\text{cpx}}P_{10\text{nt}}(t|\text{cpx}),$$

are the first passage time distributions conditioned that the RTC resides in complex cpx for the 10 nt of interest. We approximated the first passage time distribution for each RTC complex by the dwell-time fit-function (Equation 5). Considering that *P*_10nt_(*t*|cpx) is also a summation of terms with separated characteristic timescales *T*_NA_ for each pathway NA ∈ {VFNA, FNA, SNA, VSNA}, and that each of these timescales stay within one order of magnitude from the core RTC to saturating nsp13-helicase condition (Figure 3C), we obtained the average characteristic timescale of each NA pathway *T*_NA_ and the combined probability *f*_NA_ in the RTC-nsp13 mixture, referred to as the effective parameters (see STAR Methods section “effective parameters for elongation dynamics”).

(Equation 11) $$f_{\text{VFNA}}=p_{2^{\text{*}}},\;T_{\text{VFNA}}=N\tau_{\text{VFNA}}\;f_{\text{FNA}}=\underset{\text{cpx}}{\sum }p_{\text{cpx}}{\left(p_{\text{FNA}}^{\text{cpx}}\right)}^{N},\;T_{\text{FNA}}=\frac{\sum_{\text{cpx}}p_{\text{cpx}}{\left(p_{\text{FNA}}^{\text{cpx}}\right)}^{N}N\tau_{\text{FNA}}^{\text{cpx}}}{\sum_{\text{cpx}}p_{\text{cpx}}{\left(p_{\text{FNA}}^{\text{cpx}}\right)}^{N}},\;f_{\text{SNA}}=\underset{\text{cpx}}{\sum }p_{\text{cpx}}\left[{\left(p_{\text{FNA}}^{\text{cpx}}+p_{\text{SNA}}^{\text{cpx}}\right)}^{N}-{\left(p_{\text{FNA}}^{\text{cpx}}\right)}^{N}\right],\;T_{\text{SNA}}=\frac{\sum_{\text{cpx}}p_{\text{cpx}}p_{\text{SNA}}^{\text{cpx}}{\left(p_{\text{FNA}}^{\text{cpx}}+p_{\text{SNA}}^{\text{cpx}}\right)}^{N-1}N\tau_{\text{SNA}}^{\text{cpx}}}{\sum_{\text{cpx}}p_{\text{cpx}}\left[{\left(p_{\text{FNA}}^{\text{cpx}}+p_{\text{SNA}}^{\text{cpx}}\right)}^{N}-{\left(p_{\text{FNA}}^{\text{cpx}}\right)}^{N}\right]},\;f_{\text{VSNA}}=\underset{\text{cpx}}{\sum }p_{\text{cpx}}\left[{\left(p_{\text{FNA}}^{\text{cpx}}+p_{\text{SNA}}^{\text{cpx}}+p_{\text{VSNA}}^{\text{cpx}}\right)}^{N}-{\left(p_{\text{FNA}}^{\text{cpx}}+p_{\text{SNA}}^{\text{cpx}}\right)}^{N}\right],\;T_{\text{VSNA}}=\frac{\sum_{\text{cpx}}p_{\text{cpx}}p_{\text{VSNA}}^{\text{cpx}}{\left(p_{\text{FNA}}^{\text{cpx}}+p_{\text{SNA}}^{\text{cpx}}+p_{\text{VSNA}}^{\text{cpx}}\right)}^{N-1}N\tau_{\text{VSNA}}^{\text{cpx}}}{\sum_{\text{cpx}}p_{\text{cpx}}\left[{\left(p_{\text{FNA}}^{\text{cpx}}+p_{\text{SNA}}^{\text{cpx}}+p_{\text{VSNA}}^{\text{cpx}}\right)}^{N}-{\left(p_{\text{FNA}}^{\text{cpx}}+p_{\text{SNA}}^{\text{cpx}}\right)}^{N}\right]},$$

With cpx∈{c,1,2}. For a fit to the dwell-time distributions, the expressions for the average characteristic timescales and combined probabilities Equation 11 were substituted into the dwell-time fit function Equation 7. Furthermore, we added a relative probability to enter the long-lived pause for each RTC complex $p_{\mathrm{LLP}}^{\mathrm{cpx}}$ such that $p_{\text{FNA}}^{\mathrm{cpx}}+p_{\text{SNA}}^{\mathrm{cpx}}+p_{\text{VSNA}}^{\mathrm{cpx}}+p_{\mathrm{LLP}}^{\mathrm{cpx}}=1$ and thus $\sum_{j}f_{j}=1$ for j ∈ {FNA, SNA, VSNA, LLP}. The VFNA and VSNA characteristic timescales were fixed to $T_{\text{VFNA}}=0.2\;s$ and $T_{\text{VSNA}}=4.8\;s$ respectively, so the corresponding single nucleotide timescales $\tau_{\text{VFNA}},\tau_{\text{VSNA}}^{c},\tau_{\text{VSNA}}^{2}$ were not fitted, but could be directly retrieved from Equation 11.

As explained in the results section, we kept all single nucleotide parameters in the RTC-nsp13.1 complex the same as for the core RTC, while the parameters were free variables for the RTC-nsp13.1,2 complex. The free parameters in the RTC assembly - Elongation dynamics model were the single nucleotide probabilities $p_{\text{FNA}}^{c},p_{\text{SNA}}^{c},p_{\text{VSNA}}^{c},p_{\text{FNA}}^{2},p_{\text{SNA}}^{2},p_{\text{VSNA}}^{2}$ and the single nucleotide timescales $\tau_{\text{FNA}}^{c},\tau_{\text{SNA}}^{c},\tau_{\text{FNA}}^{2},\tau_{\text{SNA}}^{2}$ for the core RTC (c) and RTC-nsp13.1,2 (2) complex and the free-energy barriers in the RTC assembly model $\Delta G_{\text{c}\to 1},\Delta G_{1\to 2},\Delta G_{2\to 2^{*}}$ .

To perform a global fit on the empirical dwell-time distributions versus nsp13-helicase concentration, we obtained the average characteristic timescales and the combined probability for each pathway at every nsp13-helicase concentration measured from the RTC Assembly - Elongation dynamics model (Equation 11 with Equation 8 substituted) and substituted these parameters into the dwell-time fit-function Equation 7 with VFNA and VSNA characteristic timescales fixed to *T*_VFNA_ = 0.2 s and *T*_VSNA_ = 4.8 s respectively. In this way, we obtained a probability density function for each condition measured, which was successfully fitted to the dwell-time distributions (Figure 3A) using the Maximum likelihood fitting routine (see STAR Methods section “maximum likelihood estimation fitting routine”).

#### Mechanochemical model for RTC nucleotide addition

The force dependence of a kinetic rate can be captured by the Arrhenius law

(Equation 12) $$k_{X}(F)=k_{X,0}e^{\delta_{X}F/k_{B}T},$$

Where $\delta_{X}$ is the distance to the transition state and $k_{X,0}$ is the rate at zero force. In the case of a dsRNA template, the force (tension) is applied on the non-template RNA strand, which acts as an assisting force on elongation by the RTC.

Our mechanochemical model (Figure 4G) is derived based on the trends observed in the characteristic timescales and probabilities versus RNA tension with or without saturating nsp13-helicase concentration (results, Figure 4C–4F, STAR Methods section “assembly of the RTC-nsp13 complex”). We only have a force (RNA tension) dependence on the backward translocation rates in the FNA and SNA pathway, because Bera et al.^16^ found that the distance to the transition state is negligible for forward translocations by the RTC. As translocation acts over the distance from the conversion of one base pair dsRNA to ssRNA *a*≈0.2 nm,^21^ the opposing RNA tension (*F*) dependency on the backward translocation rate could be described as the Arrhenius law with distance -*a*

(Equation 13) $$k_{\mathrm{pre}}^{\mathrm{NA,cpx}}\left(F\right)=k_{\mathrm{pre}}^{\mathrm{NA,cpx}}\left(0\right)e^{-\mathrm{aF}/k_{B}T},\mathrm{NA}\in \left\{\mathrm{FNA},\mathrm{SNA}\right\},\mathrm{cpx}\in \left\{c,1,2\right\}.$$

To obtain the expressions for the timescale and probability for a single nucleotide addition in the pathways that showed an RNA tension dependency in the characteristic timescale (FNA and SNA) for each RTC complex cpx∈{c,1,2}, we constructed the first passage time distribution for completing the irreversible step in the NA pathway. Considering that translocation is not equilibrated, the first passage time (FPT) distributions was obtained by summing over all possible paths with forward and backward translocation (with rates $k_{\text{post}}^{\mathrm{NA,cpx}}$ and $k_{\mathrm{pre}}^{\mathrm{NA,cpx}}(F)$ respectively) followed by NTP binding and incorporation with rate $k_{\mathrm{irr}}^{\mathrm{NA,cpx}}$ (Figure 4G, STAR Methods section “building a mechanochemical model”). From this FPT distribution, we obtained the probability and timescale of completing the irreversible step in pathway NA∈{FNA,SNA}

(Equation 14) $$p_{\mathrm{irr}}^{\mathrm{NA,cpx}}=\frac{k_{\mathrm{irr}}^{\mathrm{NA}}k_{\mathrm{post}}^{\mathrm{NA}}}{\left(k_{\mathrm{post}}^{\mathrm{NA}}+k_{\mathrm{out}}^{\mathrm{NA}}\right)\left(k_{\mathrm{pre}}^{\mathrm{NA,cpx}}(F)+k_{\mathrm{irr}}^{\mathrm{NA}}\right)-k_{\mathrm{post}}^{\mathrm{NA}}k_{\mathrm{pre}}^{\mathrm{NA,cpx}}(F)},\;\tau_{\mathrm{irr}}^{\mathrm{NA,cpx}}=\frac{k_{\mathrm{post}}^{\mathrm{NA}}+k_{\mathrm{out}}^{\mathrm{NA}}+k_{\mathrm{pre}}^{\mathrm{NA,cpx}}(F)+k_{\mathrm{irr}}^{\mathrm{NA}}}{\left(k_{\mathrm{post}}^{\mathrm{NA}}+k_{\mathrm{out}}^{\mathrm{NA}}\right)\left(k_{\mathrm{pre}}^{\mathrm{NA,cpx}}(F)+k_{\mathrm{irr}}^{\mathrm{NA}}\right)-k_{\mathrm{post}}^{\mathrm{NA}}k_{\mathrm{pre}}^{\mathrm{NA,cpx}}(F)},\;\text{With}\;k_{\mathrm{out}}^{\mathrm{NA}}=k_{\mathrm{in}}^{\mathrm{SNA}}+k_{\mathrm{in}}^{\mathrm{LLP}};k_{\mathrm{out}}^{\mathrm{SNA}}=k_{\mathrm{in}}^{\mathrm{VSNA}}+k_{\mathrm{in}}^{\mathrm{LLP}}$$

Considering that the slowest pathway entered is dominating the timescale of the single nucleotide addition, we substituted $\tau_{\mathrm{NA}}^{\mathrm{cpx}}=\tau_{\mathrm{irr}}^{\mathrm{NA,cpx}}$. To obtain the single nucleotide probability for each pathway NA∈{FNA,SNA,VSNA} and the RTC complexes $\mathrm{cpx}\in \left\{1,2,2^{*}\right\}$, we had to consider how the pathways are connected. Each NA cycle starts from the FNA pre-translocated state, from which the SNA pre-translocated state can be entered and the VSNA pre-translocated state from there (Figure 4G). Furthermore, the long-lived pause can be entered from each NA pre-translocated state. With these connections, the single nucleotide probabilities for exiting through the pathways were obtained as

(Equation 15) $$p_{\mathrm{FNA}}^{\mathrm{cpx}}(F)=p_{\mathrm{irr}}^{\mathrm{FNA,cpx}}(F),\;p_{\mathrm{SNA}}^{\mathrm{cpx}}(F)=\left(1-p_{\mathrm{irr}}^{\mathrm{FNA,cpx}}(F)\right)\frac{k_{\mathrm{in}}^{\mathrm{SNA}}}{k_{\mathrm{out}}^{\mathrm{FNA}}}p_{\mathrm{irr}}^{\mathrm{SNA},\mathrm{cpx}}(F),\;p_{\mathrm{VSNA}}^{\mathrm{cpx}}(F)=\left(1-p_{\mathrm{irr}}^{\mathrm{FNA,cpx}}(F)\right)\frac{k_{\mathrm{in}}^{\mathrm{SNA}}}{k_{\mathrm{out}}^{\mathrm{FNA}}}\left(1-p_{\mathrm{irr}}^{\mathrm{SNA},\mathrm{cpx}}(F)\right)\frac{k_{\mathrm{in}}^{\mathrm{VSNA}}}{k_{\mathrm{out}}^{\mathrm{SNA}}}p_{\mathrm{irr}}^{\mathrm{VSNA,cpx}},\;p_{\mathrm{LLP}}^{\mathrm{cpx}}(F)=1-p_{\mathrm{FNA}}^{\mathrm{cpx}}(F)-p_{\mathrm{SNA}}^{\mathrm{cpx}}(F)-p_{\mathrm{VSNA}}^{\mathrm{cpx}}(F).$$

The allosteric effect of nsp13.2 binding to the RTC, resulting in decreased FNA and SNA single nucleotide timescale accompanied by a decreased SNA and VSNA probability for the RTC-nsp13.1,2 complex (Table 1), can be modeled as an increase in the free-energy barrier $\Delta G_{\text{NA}}$ for backward translocation rate in each NA pathwa*y*

(Equation 16) $$k_{\mathrm{pre}}^{\mathrm{NA},2}(F)=k_{\mathrm{pre}}^{\mathrm{NA},C}(F)e^{\Delta G_{\mathrm{NA}}},\mathrm{NA}\in \{\mathrm{FNA},\mathrm{SNA}\}.$$

Since the VSNA characteristic timescale was fixed in the fits, the underlying mechanochemistry cannot be extracted from it, so we directly fit $p_{\text{irr}}^{\text{VSNA,cpx}}$ for cpx∈{1,2}.

As a result, the free parameters in the mechanochemical model were $k_{\text{in}}^{\text{SNA}},k_{\text{in}}^{\text{VSNA}},k_{\text{in}}^{\text{LLP}},k_{\text{post}}^{\text{FNA}},k_{\text{post}}^{\text{SNA}},k_{\text{pre}}^{\text{FNA.c}}(0),k_{\text{pre}}^{\text{FNA.2}}(0),k_{\text{pre}}^{\text{SNA.c}}(0)$, $k_{\text{pre}}^{\text{SNA.}2}(0),k_{\text{irr}}^{\text{FNA}},k_{\text{irr}}^{\text{SNA}},p_{\text{irr}}^{\text{VSNA.c}},p_{\text{irr}}^{\text{VSNA.}2}$ giving the single nucleotide probabilities and timescales. The substitution into the RTC Assembly - Elongation dynamics model added the free-energy barriers between the RTC states $\Delta G_{c\to 1},\Delta G_{1\to 2},\Delta G_{2\to 2^{*}}$

A global fit of the mechanochemical model was successfully performed on the dwell-time distributions of the nsp13-helicase concentration dependency dataset at 20 pN RNA tension and RNA tension dependence datasets without nsp13-helicase and at saturating nsp13-helicase concentration (20 nM) (Figures 3A, 3C, 3D, 4C–4F, and S5N–S5P). To fit the dwell-time distributions for each condition measured we had to obtain a fit-function for each condition measured. For this, we substituted the expressions for the single nucleotide probabilities and timescales versus RNA tension (Equations 13, 14, 15, and 16) in the RTC Assembly - Elongation dynamics model (Equation 11) to obtain the effective probabilities and timescales of each pathway on the dwell-time level and these parameters were substituted into the dwell-time fit-function Equation 7. A more thorough explanation of the argumentation for and derivation of the mechanochemical model can be found in STAR Methods section “building a mechanochemical model”.

#### Bootstrapping, fitting, and fit selection

For each model fit on the empirical dwell-time distributions, we performed multiple fits and selected the best fit. Then we performed 100 fits for each model on resampled dwell-time distributions, referred to as bootstrapping, from which we obtained the mean value and standard error of the mean (SEM) on each parameter in the fitted model reported in Tables 1, S2, and S3. With the goal to minimize the number of free parameters in the model without reducing the goodness-of-fit, we set certain parameters constant during the fitting and compared them by visual inspection and using the Bayesian Information Criterion (BIC) as a guide.

#### Dwell-time fit-functions for RNA synthesis

Before building the complete first-passage-time distributions for the nucleotide addition cycle of the SARS-CoV-2 RTC in complex with nsp13-helicase, we considered the distribution for the core RTC alone. In that case, a typical dwell-time window was traversed by a mix of FNA, SNA and VSNA cycles, which we approximated as having well-separated characteristic timescales (*τ*_FNA_≪*τ*_SNA_≪*τ*_VSNA_) (Figure 1E). This approximation was justified by the timescales visible in Figure 3A.

#### Working in Laplace space

Dwell-times correspond to the first-passage times from entry to exit of the dwell-time window. For dwell-time windows including many NA steps it becomes convenient to work with first-passage time distributions in Laplace space. In Laplace space the convolutions that arise when calculating the probability density of the total time to perform sequential steps become multiplications. An exponential process with characteristic time *τ* and probability *p* and has the probability-density function (pdf) $P(t)=pe^{-t/\tau }/\tau$ and its Laplace transform is given by

(Equation 17) $$\psi (s)=\overset{\infty }{\underset{0}{\int }}\text{d}tP(t)e^{-st}=\frac{p}{1+\tau s},$$

With two sequential steps with respective pdfs $P_{1}\left(t_{1}\right)$ and $P_{2}\left(t_{2}\right)$, the Laplace transform of the pdf $P_{1+2}(t)$ of the total time $t=t_{1}+t_{2}$ is simply given by

(Equation 18) $$\psi_{1+2}(s)=\frac{p_{1}}{1+\tau_{1}s}\frac{p_{2}}{1+\tau_{2}s}.$$

The above can be done repeatedly for any number *N* of processes, resulting in

(Equation 19) $$\psi_{1+2+\cdots +N}(s)=\frac{p_{1}}{1+\tau_{1}s}\frac{p_{2}}{1+\tau_{2}s}\cdots \frac{p_{N}}{1+\tau_{N}s}.$$

The Laplace transform can in principle be inverted to give the exact form of $P_{1+2+\cdots +N}(t)$, but this can be computationally expensive and we instead applied approximations in Laplace space before reverting back to real time analytically.^47^

#### Exact dwell-time distribution for FNA, SNA, and VSNA

We started by constructing the first passage time distribution for short and intermediate (s&i) timescales by considering elongating across a *N* nucleotides (nt) window that randomly alternates through FNA, SNA, VSNA in each step.^16^ The first-passage time distribution for the core RTC in Laplace space is

(Equation 20) $$\psi_{N\text{nt}}^{\text{S\&i}}(s)=(\frac{p_{\text{FNA}}}{1+\tau_{\text{FNA}}S}+{\frac{p_{\text{SNA}}}{1+\tau_{\text{SNA}}S}+\frac{p_{\text{VSNA}}}{1+\tau_{\text{VSNA}}S})}^{N}\;=\underset{\psi_{N\text{nt}}^{\text{FNA}}\;\text{:only FNA Steps}}{\underset{︸}{{\left(\frac{p_{\text{FNA}}}{1+\tau_{\text{FNA}}S}\right)}^{N}}}+\underset{\psi_{N\text{nt}}^{\text{SNA}}\text{:at least one SNA but no VSNA steps}}{\underset{︸}{\overset{N-1}{\underset{n_{0}=0}{\sum }}\left(\begin{array}{c} N\\ n_{0},N-n_{0},0 \end{array}\right){\left(\frac{p_{\text{FNA}}}{1+\tau_{\text{FNA}}S}\right)}^{n_{0}}{\left(\frac{p_{\text{SNA}}}{1+\tau_{\text{SNA}}S}\right)}^{N-n_{0}}}}\;\underset{\psi_{N\text{nt}}^{\text{VSNA}}\text{:at least one VSNA step}}{\underset{︸}{+\overset{N-1}{\underset{n_{0}=0}{\sum }}\overset{N-n_{0}-1}{\underset{n_{1}=0}{\sum }}\left(\begin{array}{c} N\\ n_{0},n_{1},N-n_{0}-n_{1} \end{array}\right){\left(\frac{p_{\text{FNA}}}{1+\tau_{\text{FNA}}S}\right)}^{n_{0}}{\left(\frac{p_{\text{SNA}}}{1+\tau_{\text{SNA}}S}\right)}^{n_{1}}{\left(\frac{p_{\text{VSNA}}}{1+\tau_{\text{VSNA}}S}\right)}^{N-n_{0}-n_{1}}.}}$$

In order to work out the implication of the separation of timescales, we have separated out the terms that contain only FNA ($\psi_{N\text{nt}}^{\text{FNA}}$), at least one SNA but no VSNA $\left(\psi_{N\mathrm{nt}}^{\text{SNA}}\right)$, and those that contain at least one VSNA $\left(\psi_{N\mathrm{nt}}^{\text{VSNA}}\right)$.

The first term in Equation 20 $\left(\psi_{N\mathrm{nt}}^{FNA}\right)$ can readily be inverted and becomes proportional to the Gamma distribution of order N and with characteristic time $\tau_{\text{FNA}}$

(Equation 21) $$P_{N\mathrm{nt}}^{\mathrm{FNA}}(t)=p_{\mathrm{FNA}}^{N}\Gamma \left(t,\tau_{\mathrm{FNA}},N\right),\Gamma \left(t,\tau_{\mathrm{FNA}},N\right)=\frac{1}{\tau_{\mathrm{FNA}}}\frac{{\left(t/\tau_{\mathrm{FNA}}\right)}^{N-1}e^{-t/\tau_{\mathrm{FNA}}}}{(N-1)!}.$$

This distribution has the peak positioned at $T_{\text{FNA}}=N\tau_{\text{FNA}}$, which covers the single dominant FNA peak observed for short dwell-times in absence of nsp13-helicase (Figure 1E).

#### Approximation capturing the dominant behavior

With a separation of timescales we did not need to include the effects of short timescales when considering the behavior around a longer timescale. This corresponds to the statement that a slowly varying distribution is not much effected by being convoluted by a (relatively) narrow distribution. For the purpose of a fit-function that captures the dominant behavior at each timescale by setting sub-dominant timescales to zero: $\tau_{\text{FNA}}=0$ in $\psi_{N\text{nt}}^{\text{FNA}}$ and $\tau_{\text{FNA}}=\tau_{\text{SNA}}=0$ in $\psi_{N\text{nt}}^{\text{SNA}}$, resulting in

(Equation 22) $$\psi_{N\text{nt}}^{\text{SNA}}\approx \overset{N-1}{\underset{n_{0}=0}{\sum }}\left(\begin{array}{c} N\\ n_{0},N-n_{0} \end{array}\right)p_{\text{FNA}}^{n_{0}}p_{\text{SNA}}^{N-n_{0}}{\left(\frac{1}{1+\tau_{\text{SNA}}S}\right)}^{N-n_{0}}\;\psi_{N\text{nt}}^{\text{VSNA}}\approx \overset{N-1}{\underset{n_{0}=0}{\sum }}\overset{N-n_{0}-1}{\underset{n_{1}=0}{\sum }}\left(\begin{array}{c} N\\ n_{0},n_{1},N-n_{0}-n_{1} \end{array}\right)p_{\text{FNA}}^{n_{0}}p_{\text{SNA}}^{n_{1}}p_{\text{VSNA}}^{N-n_{0}-n_{1}}{\left(\frac{1}{1+\tau_{\text{VSNA}}S}\right)}^{N-n_{0}-n_{1}}.$$

Considering that the two shoulders observed for intermediate timescales in the empirical dwell-time distributions are well-fitted by exponential distributions (Figure 1E), we further approximated $P_{N\mathrm{nt}}^{\mathrm{SNA}}$ and third $P_{N\mathrm{nt}}^{\mathrm{VSNA}}$ with single exponential distributions

(Equation 23) $$P_{NNt}^{\mathrm{NA}}\left(t\right)\approx \frac{q_{\mathrm{NA}}}{T_{\mathrm{NA}}}e^{-\frac{t}{T_{\mathrm{NA}}}},\mathrm{NA}\in \left\{\mathrm{SNA},\mathrm{VSNA}\right\},$$

that capture the total probabilities $q_{\mathrm{NA}}$ and the average time $T_{\mathrm{NA}}$ of each term. From the definition of the Laplace transform it follows that the total probabilities and average timescales then are

(Equation 24) $$q_{\text{NA}}=\overset{\infty }{\underset{0}{\int }}\text{d}tP_{N\text{nt}}^{\text{NA}}(t)=\psi_{N\text{nt}}^{\text{NA}}(0);\;T_{\text{NA}}=\frac{\int_{0}^{\infty }\text{d}ttP_{N\text{Nt}}^{\text{NA}}(t)}{\int_{0}^{\infty }\text{d}tP_{N\text{Nt}}^{\text{NA}}(t)}=-\partial_{s}\text{ln}\psi_{N\text{Nt}}^{\text{NA}}(0),\text{NA}\in \{\text{SNA},\text{VSNA}\}.$$

The total probabilities and timescales over a dwell-time window were then related to the single-nucleotide step probabilities and timescales through

(Equation 25) $$q_{\mathrm{FNA}}=p_{\mathrm{FNA}}^{N}\;q_{\mathrm{SNA}}={\left(p_{\mathrm{FNA}}+p_{\mathrm{SNA}}\right)}^{N}-p_{\mathrm{FNA}}^{N},\;T_{\mathrm{SNA}}=\frac{Np_{\mathrm{SNA}}{\left(p_{\mathrm{FNA}}+p_{\mathrm{SNA}}\right)}^{N-1}}{{\left(p_{\mathrm{FNA}}+p_{\mathrm{SNA}}\right)}^{N}-p_{\mathrm{FNA}}^{N}}\tau_{\mathrm{SNA}},\;q_{\mathrm{VSNA}}={\left(p_{\mathrm{FNA}}+p_{\mathrm{SNA}}+p_{\mathrm{VSNA}}\right)}^{N}-{\left(p_{\mathrm{FNA}}+p_{\mathrm{SNA}}\right)}^{N},\;T_{\mathrm{VSNA}}=\frac{Np_{\mathrm{VSNA}}{\left(p_{\mathrm{FNA}}+p_{\mathrm{SNA}}+p_{\mathrm{VSNA}}\right)}^{N-1}}{{\left(p_{\mathrm{FNA}}+p_{\mathrm{SNA}}+p_{\mathrm{VSNA}}\right)}^{N}-{\left(p_{\mathrm{FNA}}+p_{\mathrm{SNA}}\right)}^{N}}\tau_{\mathrm{VSNA}}.$$

#### Short time cut-off due to dwell-time window size

The approximation of the dwell-time distribution, derived in Equations 20, 21, 22, 23, 24, and 25 is constructed to capture the dominant behavior around $T_{\text{SNA}}$ and $T_{\text{VSNA}}$, and breaks down for $t<T_{\text{FNA}}$ and $t<T_{\text{SNA}}$ respectively. The short time behavior still grows no faster than $∼t^{N-1}$ since N sequential steps always need to be taken to get through the dwell-time window. This behavior will always be dominated by the FNA peak, and we ensured this also in our approximation by multiplying the exponential functions with

(Equation 26) $$Q\left(t/T_{\mathrm{FNA}}\right)=\frac{{\left(t/T_{\mathrm{FNA}}\right)}^{N-1}}{1+{\left(t/T_{\mathrm{FNA}}\right)}^{N-1}},$$

to ensure the appropriate short time behavior. To keep the proper normalization from $T_{\text{FNA}}$ we took

(Equation 27) $$P_{N\mathrm{nt}}^{\mathrm{SNA}}\left(t\right)\approx Q\left(t/T_{\mathrm{FNA}}\right)\frac{q_{\mathrm{SNA}}}{T_{\mathrm{SNA}}}e^{-\frac{t-T_{\mathrm{FNA}}}{T_{\mathrm{SNA}}}},P_{N\mathrm{nt}}^{\mathrm{VSNA}}\left(t\right)\approx Q\left(t/T_{\mathrm{FNA}}\right)\frac{q_{\mathrm{VSNA}}}{T_{\mathrm{VSNA}}}e^{-\frac{t-T_{\mathrm{FNA}}}{T_{\mathrm{VSNA}}}}.$$

With this correction we arrived at the fit-function that appropriately captures the dominant behavior for short and intermediate timescales (Equation 6).

#### Accounting for long-time pauses

In addition to a peak and two shoulders, a tail of long dwell-times is observed in the empirical dwell-time distributions for elongation dynamics by the core RTC with a decay of order $∼t^{-3/2}$ over orders of magnitude (10–1000 s) (Figure 3B). This trend suggests we observed long-lived pauses resulting from random walks that are recovered, such as backtracking.^31^ The first-passage time of backtrack recovery represents the time it takes to re-align with the 3′ end of the template after a random walk on the RNA and re-initiate RNA synthesis. Defining the stepping rate toward realignment as *k*_f_ (forward) and away from realignment as *k*_b_ (backward), the first passage time distribution for such a random walk can be split into three regimes as shown by Depken et al.^31^ For the low-time regime $t\ll t_{1}=1/\sqrt{k_{f}k_{b}}$, the distribution is flat; in the intermediate-time regime $t_{1}\ll t\ll t_{2}=1/{\left(\sqrt{k_{f}}-\sqrt{k_{b}}\right)}^{2}$ the distribution decays as a power law $∼t^{-3/2}$ and for $t\gg t_{2}$ the distribution is exponentially cut-off with characteristic timescale $t_{2}$.^31^

We never observed the transition into the short time regime, suggesting that *t*_1_ is in a regime where the behavior is dominated by either of the FNA, SNA or VSNA pathways ($t_{1}\ll T_{\text{VSNA}}\approx 5s$ ). We also never observed the exponential cut-off for large timescales, suggesting that $t_{2}$ is larger than the total measurement time ( $t_{2}\gg 1000\;s$ ). The fact that the transitions to the short and to the long time regime were not observed means that $t_{2}/t_{1}\gg 200$. If $k_{f}<k_{b}$ the average long-lived pause duration and extension would diverge. As we did not see evidence of this, we concluded that $k_{f}>k_{b}$. The simplest model for the forward-biased random walk stepping rates dependence on RNA tension ( F ) is that they follow an Arrhenius law

(Equation 28) $$k_{\text{f}}=k_{0}e^{F\delta /k_{B}T},k_{\text{b}}=k_{0}e^{-F(d-\delta )/k_{B}T}\Rightarrow k_{\text{f}}/k_{\text{b}}=e^{Fd/k_{B}T},\sqrt{k_{\text{f}}k_{\text{b}}}=k_{0}e^{F(\delta -d/2)/k_{B}T}.$$

Using that $t_{1}=1/\sqrt{k_{f}k_{b}}$ and $t_{2}=1/{\left(\sqrt{k_{f}}-\sqrt{k_{b}}\right)}^{2}$, we see that $t_{2}/t_{1}\gg 200$ means $1<k_{f}/k_{b}<1.2$. The fact that the ratio between stepping rates never exceeded 1.2 even for the highest tension (40 pN) implies that *d* < 0.02 nm. If we assumed the distance to the transition state *δ* lies withing the step (0<*δ*<*d*), this would in turn imply that *t*_1_ only appreciably shifts for forces above $\frac{k_{B}T}{0.02\;nm/2}\approx 400pN$ . For our empirical conditions we should therefore be safe assuming that *t*_1_ does not depend appreciably on force, and that ratios of amplitudes faithfully report on ratios of probabilities.

The upper limit for *d* is significantly smaller than the length change from double-stranded to single-stranded RNA per nucleotide (*a*≈0.2 nm), indicating that the energy landscape for the long-lived pause is rugged and a full nucleotide step (0.2 nm) could consist of many intermediate shorter steps before the translocation is complete.^48^ As a result, we could not say how far the backtrack of the RTC goes during the long-lived pause and it could even stay on the same position while going through many conformations with average distance *d* < 0.02 nm.

For the purpose of capturing the power-law decay in the long time regime and the sub-dominance at shorter timescales, the first-passage time distribution of the long-lived pause (LLP) recovery is approximated by a power law decay with amplitude *q*_LLP_, cut-off at short timescales^31^

(Equation 29) $$P_{N\mathrm{nt}}^{\mathrm{LLP}}(t)=\frac{q_{\mathrm{LLP}}}{{\left(1+t/t_{1}\right)}^{3/2}}.$$

The amplitude *q*_LLP_ of the power law decay is proportional to the total probability for entering the long-lived pause in a dwell-time window, but the constant of proportionality is sensitive to the undetermined cut-off time *t*_1_. As long as *t*_1_ does not change, ratios of amplitudes correspond to ratios of probabilities, so we treated *q*_LLP_ as a relative probability.

As for the other intermediate time processes we accounted for the fact that a dwell-time window consists of 10 nt and the contribution of long-lived pauses at short times must be cut-off with a regularization function (Equation 26). Properly normalizing the contribution to exclude times shorter than *T*_FNA_ we arrived at the dwell-time fit-function for the core RTC dynamics alternating (alt) between NA pathways and long-lived pauses (LLPs).

(Equation 30) $$P_{N\text{nt}}^{\text{alt}}(t,p)\approx P_{N\text{nt}}^{\text{s\&i}}(t)+Q\left(\frac{t}{T_{\text{FNA}}}\right)\frac{1}{2}\sqrt{1+\frac{T_{\text{FNA}}}{t_{1}}}P_{\text{LLP}}(t).$$

Here we have introduced the parameter vector $p=\left\{q_{\text{FNA}},q_{\text{SNA}},q_{\text{VSNA}},q_{\text{LLP}},T_{\text{FNA}},T_{\text{SNA}},T_{\text{VSNA}}\right\}$ for later convenience. The weights satisfy $q_{\text{FNA}}+q_{\text{SNA}}+q_{\text{VSNA}}+q_{\text{LLP}}=1$..

As we could not obtain the actual value of *t*_1_ from our data, but the value also does not affect the shape of the long-lived pause recovery distribution where it is dominant, we simply set *t*_1_ = 1 s, but recognized that *q*_LLP_ cannot be interpreted as a probability other than that ratios of amplitudes correspond to ratios of probabilities as described above.

The expression for $P_{N\mathrm{nt}}^{\text{core}}(t,p)$ is strictly only valid for observations that are not limited by the measurement time or resolution. Since the resolution $t_{\text{cut}}=0.08s$ and measurement time *t_max_* = 1000 s were finite, the dwell-time fit-function was normalized to this interval for the purpose of the fits^16^

(Equation 31) $$P_{N\mathrm{nt}}^{\text{core fit}}\left(t,p\right)\approx \frac{P_{N\mathrm{nt}}^{\text{alt}}\left(t,p\right)}{\int_{t_{\text{cut}}}^{t_{max}}d\tau P_{N\mathrm{nt}}^{\text{alt}}\left(\tau ,p\right)}.$$

#### Fit-function for saturating nsp13 helicase

The dwell-time fit-function Equation 6 normalized as in Equation 31 including the FNA, SNA, VSNA pathway and long-lived pauses fitted well to the empirical dwell-time distribution for core RTC elongation dynamics (Figure 1E), so we set out to extend it in presence of nsp13-helicase.

For saturating concentrations of nsp13-helicase, we observed bursts of very fast nucleotide addition (VFNA), in addition to the nucleotide addition signatures observed for the core RTC (Figures 1D and 3A). These bursts of VFNA resulted in a peak for very short dwell-times (Figures 1D and 1E). Since the bursts last longer than the dwell-time window (Figure 1C), the VFNA pathway was added to the dwell-time fit-function as a separate gamma pdf with *N* steps and timescale *τ*_VFNA_. After addition of the VFNA peak, the unnormalized fit-function reads

(Equation 32) $$P_{N\mathrm{nt}}^{\text{sat}}\left(t,p^{\text{sat}},f_{\text{VFNA}}^{\text{sat}},T_{\text{VFNA}}^{\text{sat}}\right)\approx f_{\text{VFNA}}^{\text{sat}}\Gamma \left(t,T_{\text{VFNA}}/N,N\right)+\left(1-f_{\text{VFNA}}^{\text{sat}}\right)P_{N\mathrm{nt}}^{\text{alt}}\left(t,p^{\text{sat}}\right).$$

The first part of this distribution is a gamma distribution with peak at $T_{\text{VFNA}}=N\tau_{\text{VFNA}}$, where $\tau_{\text{VFNA}}$ is the single nucleotide addition rate. Normalization to the observational interval gave us the final dwell-time fit-function at saturating concentrations of nsp13-helicase

(Equation 33) $$P_{N\text{nt}}^{\text{fit}}\left(t,p^{\text{sat}},f_{\text{VFNA}}^{\text{sat}},T_{\text{VFNA}}^{\text{sat}}\right)\approx \frac{P_{N\text{nt}}^{\text{sat}}\left(t,p^{\text{sat}},f_{\text{VFNA}}^{\text{sat}},T_{\text{VFNA}}^{\text{sat}}\right)}{\int_{t_{\text{cut}}}^{t_{\text{max}}}d\tau P_{N\text{nt}}^{\text{sat}}\left(\tau ,p^{\text{sat}},f_{\text{VFNA}}^{\text{sat}},T_{\text{VFNA}}^{\text{sat}}\right)}.$$

#### Fit-function for non-saturating nsp13 helicase

While our dwell-time fit-function was constructed for RTC-nsp13 complexes at saturating nsp13-helicase concentrations, titration of the nsp13-helicase concentration leaded to a mixing of timescales pertaining to the different RTC-nsp13 complexes. Still, the dwell-time distributions at non-saturating nsp13-helicase concentrations were well-captured by the fit-function constructed for saturating nsp13-helicase conditions (Figure 3A), indicating that the variation in timescales for a particular process remained small compared to differences in timescales between processes. We therefore used the dwell-time fit-function for saturating nsp13-helicase concentrations (Equations 32 and 33) also for non-saturating nsp13-helicase concentrations, but with parameters depending on the concentration

(Equation 34) $$P_{N\text{nt}}^{\text{fit}}\left(t,p^{\left[\text{nsp13}\right]},f_{\text{VNAA}}^{\left[\text{nsp13}\right]},T_{\text{VFNA}}^{\left[\text{nsp13}\right]}\right).$$

The timescales and probabilities of the slow to intermediate pathways $p^{\left[\text{nsp13}\right]}$ were extracted as averages over the complexes involved. How these averages should be performed is explained in STAR Methods section “effective parameters for elongation dynamics”.

#### Setting parameters constant

We started by performing fits on various nsp13-helicase concentrations (and RNA tension) with all parameters free. This resulted in good fits (Figures S5D–S5F), but no significant trend was observed in the VFNA and VSNA characteristic timescale (Figure S2D). A constant *T*_VFNA_ is consistent with each burst arising from a single RTC complex. The lack of any significant trend in the VSNA characteristic timescale could simply be due to that the region where VSNA dominates was generally very small, and the characteristic timescale was hard to determine (Figure S5). To reduce overfitting we fixed the VFNA characteristic timescale (0.2 s) and the VSNA characteristic timescale (4.8 s) without apparent reduction in goodness-of-fit (Figures S5G–S5I) or shifts in the other parameters (Figures S2K–S2R). For consistency, we also put the VSNA characteristic timescale fixed in the fits for RTC elongation dynamics on ssRNA (Figure S5C).

#### Assembly of the RTC-nsp13 complex

We observed no bursts of dsRNA opening for Poliovirus RdRP with nsp13-helicase in the reaction mixture at high concentration ([nsp13] = 50 nM) (Figures 2A, S2A, and S2B). This suggested that the bursts observed for CoV RTC in the presence of nsp13-helicase are the result of complex formation and not due to nsp13-helicase interacting directly with the RNA to unwind the RNA duplex. There was also a slowdown of RTC elongation dynamics upon introduction of an ATPase-dead mutant of nsp13-helicase (Figures 2B, S2A, and S2B), confirming that nsp13-helicase forms a complex with the core RTC. For RTC elongation on a ssRNA template in presence of active nsp13-helicase, we observed significantly faster overall dynamics, but no bursts of nucleotide addition (Figure 2C). This absence of bursts supports the conclusion that the VFNA pathway is the result of an interaction of nsp13-helicase with the non-template RNA strand (Figure 2D). Taken together, these results showed that nsp13-helicase associates with the RTC and engages with the non-template strand to produce elongation bursts (Figure 3B).

The fact that, in addition to the VFNA peak, the signatures of all other processes remain apparent also in the dwell-time distributions at saturating nsp13-helicase concentrations (Figure 3A) suggested that the nsp13-RTC complex can exist in two forms: an engaged nsp13-RTC complex, which results in the VFNA pathway, and a non-engaged two nsp13-bound RTC which has the same nucleotide addition pathways as the core RTC. Cryo-EM structural studies^4,10,15^ have shown that nsp13.1 first binds to the core RTC and a conformational change has to occur before nsp13.2 can bind. Moreover, the structural studies found that nsp13.1 is interacting with the template RNA strand, which implies that nsp13.2 is interacting with the non-template RNA strand (Figure 3B).

To establish if the RTC needs one or two nsp13-helicases to engage with the non-template strand in the VFNA pathway, we tested two RTC-nsp13 assembly models (Figure S3E) on our data.

**Assembly model 1:** nsp13.1 binds to the core RTC (RTC-nsp13.1) and engages with the non-template RNA strand (RTC-nsp13.1*) enabling elongation bursts.

**Assembly model 2:** nsp13.1 binds to the core RTC (RTC-nsp13.1), nsp13.2 binds to RTC-nsp13.1 (RTC-nsp13.1,2) and nsp13.2 engages with the non-template RNA strand (RTC-nsp13.1,2*) enabling elongation bursts.

To discriminate between assembly models, we assumed the binding of two nsp13-helicases is in dynamic equilibrium, but they do not exchange within one dwell-time window. We use Boltzmann statistics to calculate the fractional occupancies of the RTC complexes for our two models:

For **Assembly model 1** the fractional occupancies of each RTC complex were calculated as

(Equation 35) $$p_{c}([\mathrm{nsp}13])=\frac{1}{Z([\mathrm{nsp}13])},p_{1}([\mathrm{nsp}13])=\frac{[\mathrm{nsp}13]e^{-\Delta G_{c\to 1}}}{Z([\mathrm{nsp}13])},p_{1^{*}}([\mathrm{nsp}13])=\frac{[\mathrm{nsp}13]e^{-\left(\Delta G_{c\to 1}+\Delta G_{1\to 1^{*}}\right)}}{Z([\mathrm{nsp}13])},$$

With c representing the core RTC, 1 the complex with one nsp13, but no engagement, and 1* the complex with engagement of the first nsp13. We have also introduced the normalization constant $Z([\mathrm{nsp}13])=[\mathrm{nsp}13]e^{-\left(\Delta G_{c\to 1}+\Delta G_{1\to 1^{*}}\right)}+[\mathrm{nsp}13]e^{-\Delta G_{c\to 1}}+1$, with $\Delta G_{\mathrm{cpx}\to {\mathrm{cpx}}^{\prime }}$ representing the free-energies differences between the sequential RTC complexes cpx∈{c,1} and ${\mathrm{cpx}}^{\prime }\in \left\{1,1^{⋆}\right\}$ with 1 nM as reference concentration, expressed in units of $k_{B}T$.

For **Assembly model 2** the fractional occupancies of each RTC complex were calculated as

(Equation 36) $$p_{c}([\text{nsp}13])=\frac{1}{Z([\text{nsp}13])},p_{1}([\text{nsp}13])=\frac{[\text{nsp}13]e^{-\text{Δ}G_{c\to 1}}}{Z([\text{nsp}13])},\;p_{2}([\text{nsp}13])=\frac{{\left[\text{nsp}13\right]}^{2}e^{-\left(\text{Δ}G_{c\to 1}+\text{Δ}G_{1\to 2}\right)}}{Z([\text{nsp}13])},\;p_{2^{\text{*}}}([\text{nsp}13])=\frac{{\left[\text{nsp}13\right]}^{2}e^{-\left(\text{Δ}G_{c\to 1}+\text{Δ}G_{1\to 2}+\text{Δ}G_{2\to 2^{\text{*}}}\right)}}{Z([\text{nsp}13])},$$

With c representing the core RTC, 1 the complex with one nsp13, 2 the complex with two nsp13-helicases but no engagement, and 2* the complex with two nsp13-helicases and engagement of the second nsp13. We have also introduced the normalization constant $Z([\mathrm{nsp}13])=[\mathrm{nsp}13{]}^{2}e^{-\left(\Delta G_{c\to 1}+\Delta G_{1\to 2}+\Delta G_{2\to 2^{*}}\right)}+[\mathrm{nsp}13{]}^{2}e^{-\left(\Delta G_{c\to 1}+\Delta G_{1\to 2}\right)}+[\mathrm{nsp}13]e^{-\Delta G_{c\to 1}}+1$, with $\Delta G_{\mathrm{cpx}\to {\mathrm{cpx}}^{\prime }}$ representing the free-energies differences between the sequential RTC complexes cpx∈{c,1,2} and ${\mathrm{cpx}}^{\prime }\in \left\{1,2,2^{*}\right\}$ with 1 nM as reference concentration, expressed in units of $k_{B}T$.

We performed fits of the fractional occupancy of the nsp13-engaged RTC state for model 1 and 2 (*p*_1_* and *p*_2_* respectively) to the probability of the VFNA pathway versus nsp13-helicase concentration $f_{\text{VFNA}}^{\left[\text{nsp13}\right]}$ by minimizing the error-weighted square deviation (Figure S3F). We concluded that Assembly model 2 fits better (Table S2), which is largely due to its quadratic dependence of *p*_2_* on nsp13-helicase concentration at low concentrations (Equation 36).

Moreover, the fractional occupancy of nsp13.1,2* *p*_2_* fitted well to the VFNA probability $f_{\text{VFNA}}^{\left[\text{nsp13}\right]}$ with nsp13M4 concentration (Figure S3G), confirming the model for assembly of the RTC-nsp13 complex and showing that the engagement of nsp13.2 with the non-template strand is affected by the M4 mutation.

#### Effective parameters for elongation dynamics

At intermediate nsp13-helicase concentrations, dwell-time distributions shift based on the stoichiometry of the various RTC complexes, and not per se due to a shift in the elongation dynamics by individual complexes. Since the complexes have fractional occupancies as in Equation 36, the shift in the dwell-time distribution is captured by

(Equation 37) $$P_{N\text{nt}}^{\text{mix}}(t)=p_{2^{\text{*}}}([\text{nsp}13])\Gamma \left(t,T_{\text{VFNA}}/N,N\right)+\underset{\text{cpx}\in \text{C}}{\sum }p_{\text{cpx}}([\text{nsp}13])P_{N\text{nt}}^{\text{alt}}\left(t,p^{\text{cpx}}\right),\text{cpx}\in \{\text{c},1,2\}.$$

For a mix of complexes we observe that Equation 34 still captures the data (Figure 3A). From this we took that the variations in characteristic timescales between models are moderate enough that they can be captured by effective timescales and probabilities. Therefore we replaced the non-bursting part of the above equation with an effective distribution

(Equation 38) $$\underset{\text{cpx}\in C}{\sum }p_{\text{cpx}}([\text{nsp}13])P_{N\text{nt}}^{\text{alt}}\left(t,p^{\text{cpx}}\right)\approx P_{N\text{nt}}^{\text{alt}}\left(t,p^{\text{eff}}([\text{nsp}13])\right),\text{cpx}\in \{\text{c},1,2\},$$

Where the effective probabilities and characteristic times $p^{\text{eff}}([\mathrm{nsp}13])$ are chosen to match the total probability and average characteristic timescale among complexes for each pathway and nsp13-helicase concentration. This resulted in

(Equation 39) $$P_{Nnt,\mathrm{cpx}}^{\text{mix,eff}}(t)=p_{2^{*}}([\mathrm{nsp}13])\Gamma \left(t;T_{V\mathrm{FNA}}/N,N\right)+P_{Nnt}^{\text{alt}}\left(t,p^{\text{eff}}([\mathrm{nsp}13])\right),\mathrm{cpx}\in \{c,1,2\},$$

With

(Equation 40) $$q_{\text{NA}}^{\text{eff}}([\text{nsp}13])=\underset{\text{cpx}\in C}{\sum }p_{\text{cpx}}([\text{nsp}13])q_{\text{NA}}^{\text{cpx}},\text{NA}\in \{\text{FNA},\text{SNA},\text{VSNA}\},\;T_{\text{NA}}^{\text{eff}}=\frac{\sum_{\text{cpx}\in C}p_{\text{cpx}}([\text{nsp}13])q_{\text{NA}}^{\text{cpx}}T_{\text{NA}}^{\text{cpx}}}{\sum_{\text{cpx}\in C}p_{\text{cpx}}([\text{nsp}13])q_{\text{NA}}^{\text{cpx}}},\text{NA}\in \{\text{FNA},\text{SNA},\text{VSNA}\}.$$

The probabilities and characteristic timescales for each pathway NA∈{FNA, SNA, VSNA} entered by complex $\mathrm{cpx}\in \{c,1,2\}\left(q_{\text{NA}}^{\mathrm{cpx}}\right.$ and $T_{\text{NA}}^{\mathrm{cpx}}$ are expressed in terms of single nucleotide probabilities and timescales as shown for the core RTC in STAR Methods section “approximation capturing the dominant behaviour” (Equation 25).

(Equation 41) $$q_{\text{FNA}}^{\text{cpx}}={\left(p_{\text{FNA}}^{\text{cpx}}\right)}^{N},\;q_{\text{SNA}}^{\text{cpx}}={\left(p_{\text{FNA}}^{\text{cpx}}+p_{\text{SNA}}^{\text{cpx}}\right)}^{N}-{\left(p_{\text{FNA}}^{\text{cpx}}\right)}^{N},\;T_{\text{SNA}}^{\text{cpx}}=\frac{Np_{\text{SNA}}^{\text{cpx}}{\left(p_{\text{FNA}}^{\text{cpx}}+p_{\text{SNA}}^{\text{cpx}}\right)}^{N-1}}{{\left(p_{\text{FNA}}^{\text{cpx}}+p_{\text{SNA}}^{\text{cpx}}\right)}^{N}-{\left(p_{\text{FNA}}^{\text{cpx}}\right)}^{N}}\tau_{\text{SNA}}^{\text{cpx}},\;q_{\text{VSNA}}^{\text{cpx}}={\left(p_{\text{FNA}}^{\text{cpx}}+p_{\text{SNA}}^{\text{cpx}}+p_{\text{VSNA}}^{\text{cpx}}\right)}^{N}-{\left(p_{\text{FNA}}^{\text{cpx}}+p_{\text{SNA}}^{\text{cpx}}\right)}^{N},\;T_{\text{VSNA}}^{\text{cpx}}=\frac{Np_{\text{VSNA}}^{\text{cpx}}{\left(p_{\text{FNA}}^{\text{cpx}}+p_{\text{SNA}}^{\text{cpx}}+p_{\text{VSNA}}^{\text{cpx}}\right)}^{N-1}}{{\left(p_{\text{FNA}}^{\text{cpx}}+p_{\text{SNA}}^{\text{cpx}}+p_{\text{VSNA}}^{\text{cpx}}\right)}^{N}-{\left(p_{\text{FNA}}^{\text{cpx}}+p_{\text{SNA}}^{\text{cpx}}\right)}^{N}}c_{\text{VSNA}}^{\text{cpx}}.$$

Since we have $q_{\mathrm{FNA}}^{\mathrm{cpx}}+q_{\mathrm{SNA}}^{\mathrm{cpx}}+q_{\mathrm{VSNA}}^{\mathrm{cpx}}+q_{\mathrm{LLP}}^{\mathrm{cpx}}=1$, we obtain from Equation 40 that $q_{\mathrm{FNA}}^{eff}+q_{\mathrm{SNA}}^{eff}+q_{\mathrm{VSNA}}^{eff}+q_{\mathrm{LLP}}^{eff}=1-p_{2^{*}}([\mathrm{nsp}13])$. To perform the fits we normalized the mixed fit function over the empirical time interval

(Equation 42) $$P_{N\mathrm{nt}}^{\text{mix},\text{fit}}(t)\approx \frac{P_{N\text{nt}}^{\text{mix},\text{eff}}(t)}{\overset{t_{\text{max}}}{\underset{t_{\text{cut}}}{\int }}\text{d}\tau P_{N\text{nt}}^{\text{mix},\text{eff}}(\tau )}.$$

With this fit-function, referred to as the RTC Assembly – Elongation dynamics model, we captured the fact that any dependence on nsp13-helicase concentration arises from a shift in stoichiometry between RTC-nsp13 complexes, and not a shift in dynamics for individual complexes.

#### Non-bursting elongation dynamics

On top of the elongation bursts (VFNA), we observed a significant decrease in the FNA and SNA characteristic timescale for RTC elongation on the dsRNA template, which saturated above 10 nM nsp13-helicase (Figure 3C). This is supported by a decrease observed in the FNA characteristic timescale for RTC elongation on ssRNA saturating only for high nsp13-helicase concentration (20 nM) (Figure S2C), indicating that the effect resulted from nsp13.2 association and impacts RTC-nsp13.1,2 elongation dynamics.

Based on these observations, we performed a global fit to the dwell-time distributions for all nsp13-helicase concentrations measured with different single nucleotide probabilities and timescales for the RTC-nsp13.1,2 complex than the core RTC and RTC-nsp13.1 complex ($p_{\text{NA}}^{1}=p_{\text{NA}}^{c},\tau_{\text{NA}}^{1}=\tau_{\text{NA}}^{c}$ with NA∈{FNA,SNA,VSNA}), referred to as the RTC Assembly – Elongation dynamics model. This resulted in a good fit (Figure S5J) that captured the trends in the dwell-time fit parameters for all pathways (Figures 3C and 3D).

To make sure that the changes in single nucleotide probabilities and timescales for the RTC-nsp13.1,2 complex are significant, we tested if the dwell-time distributions could be captured if we keep the single nucleotide probabilities and characteristic timescales fixed to the values obtained from the dwell-time fits to the core RTC data ($p_{\text{NA}}^{\mathrm{cpx}}=p_{\text{NA}}^{c},\tau_{\text{NA}}^{\mathrm{cpx}}=\tau_{\text{NA}}^{c}$ with cpx∈{1,2} and NA∈{FNA, SNA,VSNA}) or if we let only the single nucleotide probabilities for the RTC-nsp13.1,2 complex free in the fit ($p_{\text{NA}}^{1}=p_{\text{NA}}^{c},\tau_{\text{NA}}^{\mathrm{cpx}}=\tau_{\text{NA}}^{c}$ with cpx∈{1,2} and NA∈{FNA,SNA,VSNA}). Both simplifications did not result in a good fit (Figure S5KL), showing that both probabilities and timescales of the NA pathways change significantly for the RTC-nsp13.1,2 complex w.r.t. the core RTC, which was confirmed by 100 bootstrap fits (Table S3).

Additionally, from the fits of the RTC assembly - Elongation dynamics model to the dwell-time distributions for increasing nsp13M4 concentration (Figure S5R), we further obtained that the single nucleotide timescales and probabilities of the nucleotide addition pathways for the RTC-nsp13.1,2 state have similar values for nsp13M4 and wild-type nsp13-helicase (Table S3). From these fit results we conclude that the allosteric effect of nsp13-helicase binding to the RTC is unchanged by the M4 mutation.

Furthermore, we needed to check whether taking the effective characteristic timescale and probabilities for each pathway does not significantly change the probability density function (pdf) and thus the fitted parameters. In order to do this, we substituted the fitted single nucleotide parameters in the total first passage time distributions for the mix of RTC complexes (Equation 37) and compared the resulting pdf curves to the empirical dwell-time distributions. The resulting pdf’s are within one standard deviation of the empirical dwell-time distributions for most conditions (Figure S5M), so we concluded that taking the effective parameters for elongation dynamics does not affect the pdf curves significantly for the conditions measured.

#### Building a mechanochemical model

Based on the empirical dwell-time distributions (Figure 3A), we distinguished three NA pathways for the SARS-CoV-2 core RTC. Each pathway is characterized by a characteristic timescale and a probability of being entered at the start of each NA cycle (Equation 7).^16^ To build a mechanochemical model that captures the interrelated trends of single nucleotide probabilities and timescales, we started with a model where the NA cycle includes translocation, nucleotide binding, catalysis, and pyrophosphate (PPi) release (Figure S4A). Each NA cycle starts with the RTC in the pre-translocated state. From here, the RTC moves reversibly to the post-translocated state where the NTP can bind and unbind. There can be many translocation and NTP binding cycles before the RNA product is extended through irreversible NTP catalysis and PPi release (Figure S4A).

#### Mechanochemistry of the non-bursting NA pathways

As unwinding the upstream dsRNA costs energy, the presence of the replication fork introduces a bias toward the pre-translocated state. Tension on the RNA destabilizes the replication fork and thus decreases this bias. Bera et al.^16^ found that the transition state for translocation is located close to the pre-translocated state and that the change in bias originates from the tension dependence of the backward translocation rate in the FNA pathway (Figure S4A). We took this to be true for all NA pathways.

We observed that the average elongation velocity by the core RTC on dsRNA ((3.6 ± 0.1) nt/s) is much lower than on ssRNA ((51.5 ± 1.1) nt/s) (Results). This large velocity difference indicated that moving the replication fork is rate-limiting on dsRNA.^19,21^ We therefore assumed NTP binding is in rapid equilibrium.^16^ We used an effective nucleotide incorporation rate ($k_{\text{irr}}$) and backward translocation rate ($k_{pr}$) and ignored the time for PPi release (Figure S4A). With these assumptions we arrived at a simplified mechanochemical model for the NA cycles in each non-bursting NA pathway.

#### Connections between non-bursting NA pathways

With the mechanochemistry of a general NA cycle characterized, we turned to how the non-bursting NA pathways are connected. We observed that the FNA probability increased with RNA tension (Figure 4E), while the SNA and VSNA probabilities decreased. Given the increasing bias toward the post-translocated state with tension, these trends suggested that each NA cycle starts in the FNA pre-translocated state and the SNA and VSNA pathways are entered from there (Figure S4B).^16^

If both the SNA and VSNA pathway would be directly entered from the FNA pre-translocated state, we would expect their relative probability to be independent of RNA tension. Instead, we observed that the SNA probability became relatively higher than the VSNA probability with tension (Figure 4E). This suggested that the VSNA pathway is entered from the SNA pre-translocated state (Figure S4B).

Since there were no clear trends in the VSNA characteristic timescale, we could not extract the backward translocation rate. We fixed it for all conditions as explained in STAR Methods section “setting parameters constant” and simplified the pathway to a single step with a tension independent probability $p_{\text{irr}}^{\text{VSNA}}$ (Figure S4B).

#### Mechanochemistry of the bursting NA pathway

The probability of very fast nucleotide addition bursts (VFNA) was independent of RNA tension, indicating that the stability of the RTC-nsp13.1,2* complex is not impacted by increasing RNA tension. Furthermore, the characteristic timescale of the VFNA pathway showed no significant trends versus RNA tension for saturating nsp13-helicase concentration (Figures 4D and 4F) and we concluded that it is limited by an RNA tension independent step.

#### Mechanochemistry behind the allosteric effect

After deciphering the mechanochemistry and the connections between the NA pathways for the core RTC, we turned to the allosteric effect from the nsp13.2 association with the RTC. Here we assumed that the general reaction scheme is unaffected by binding of nsp13-helicase (Figure 4G), but that the rates can change. Increasing the RNA tension had no significant effect on the FNA characteristic timescale and probability for RTC-nsp13.1,2 (Figures 4D and 4F), indicating that the backward translocation rate was negligible.

The SNA characteristic timescale for RTC-nsp13.1,2 is significantly lower than for the core RTC, indicating that RTC-nsp13.1,2 spends less time in the translocation cycles. Still, the SNA characteristic timescale decreases with increasing RNA tension (Figure 4D), indicating that translocation cycles are not completely abolished, and the backward translocation rate is reduced but not negligible.

Furthermore, the VSNA probability decreased much more than the SNA and LLP probabilities, thus we let the probability for performing the irreversible step change to model the allosteric effect in our simplified description of the VSNA pathway.

#### Connections for the long-lived pause to NA pathways

The LLP probability in general showed similar tension dependent trends as the VSNA probability (Figures 4E and 4F), suggesting that the LLP state is also entered from the pre-translocated state. For simplicity, we considered the LLP is entered from the pre-translocated state of each NA pathway with a constant relative rate (Figure 4G).

#### Extracting parameters from mechanochemical model

In the upper sections we arrived at a mechanochemical model for elongation dynamics by the SARS-CoV-2 RTC with or without nsp13-helicase shown in Figure 4G. In this section we describe how the parameters in this model can be obtained from fits to the empirical dwell-time distributions.

In our mechanochemical model for elongation dynamics by the SARS-CoV-2 RTC (Figure 4G), the single nucleotide timescale of each non-bursting NA pathway corresponds to the time for multiple cycles of forward and backward translocation before completing the irreversible step, starting from the pre-translocated state. We considered that translocation is not equilibrated in the FNA and SNA pathway (STAR Methods section “mechanochemistry of the non-bursting NA pathways”). Thus to obtain the first passage time distribution for completing the irreversible step in the NA∈{FNA, SNA} pathway by RTC complex cpx∈{c,1, 2}, we summed over all possible paths with an arbitrary number of forward and backward translocations before the irreversible step of NTP binding and catalysis with rate $k_{\mathrm{irr}}^{\mathrm{NA}}$

(Equation 43) $${\text{Ψ}}_{\text{irr}}^{\text{NA,cpx}}(s)=\overset{\infty }{\underset{n=0}{\sum }}{\left(\frac{k_{\text{post}}^{\text{NA}}}{s+k_{\text{post}}^{\text{NA}}+k_{\text{out}}^{\text{NA}}}\frac{k_{\text{pre}}^{\text{NA,cpx}}(F)}{s+k_{\text{pre}}^{\text{NA,cpx}}(F)+k_{\text{irr}}^{\text{NA}}}\right)}^{n}\frac{k_{\text{post}}^{\text{NA}}}{s+k_{\text{post}}^{\text{NA}}+k_{\text{out}}^{\text{NA}}}\frac{k_{\text{irr}}^{\text{NA}}}{s+k_{\text{pre}}^{\text{NA,cpx}}(F)+k_{\text{irr}}^{\text{NA}}}=\;\frac{k_{\text{irr}}^{\text{NA}}k_{\text{post}}^{\text{NA}}}{\left(s+k_{\text{post}}^{\text{NA}}+k_{\text{out}}^{\text{NA}}\right)\left(s+k_{\text{pre}}^{\text{NA,cpx}}(F)+k_{\text{irr}}^{\text{NA}}\right)-k_{\text{post}}^{\text{NA}}k_{\text{pre}}^{\text{NA,cpx}}(F)}.$$

With the rate to enter the slower pathways $k_{\text{out}}^{\mathrm{FNA}}=k_{\mathrm{in}}^{\mathrm{SNA}}+k_{\mathrm{in}}^{\mathrm{LLP}}$ or $k_{\mathrm{out}}^{\mathrm{SNA}}=k_{\mathrm{in}}^{\mathrm{VSNA}}+k_{\mathrm{in}}^{\mathrm{LLP}}$ respectively.

From the first passage time distribution in Laplace space $\Psi_{\text{irr}}^{NA,\mathrm{cpx}}(s)$, we obtained the probability and timescales of completing the irreversible step in pathway NA∈{FNA,SNA} as the zeroth and first moment of the distribution at *s* = 0 respectively,

(Equation 44) $$p_{\mathrm{irr}}^{\mathrm{NA,cpx}}=\Psi_{\mathrm{irr}}^{\mathrm{NA,cpx}}(0)=\frac{k_{\mathrm{irr}}^{\mathrm{NA}}k_{\mathrm{post}}^{\mathrm{NA}}}{\left(k_{\mathrm{post}}^{\mathrm{NA}}+k_{\mathrm{out}}^{\mathrm{NA}}\right)\left(k_{\mathrm{pre}}^{\mathrm{NA,cpx}}(F)+k_{\mathrm{irr}}^{\mathrm{NA}}\right)-k_{\mathrm{post}}^{\mathrm{NA}}k_{\mathrm{pre}}^{\mathrm{NA,cpx}}(F)},\;\tau_{\mathrm{irr}}^{\mathrm{NA,cpx}}=-\partial_{s}\ln\Psi_{\mathrm{irr}}^{\mathrm{NA,cpx}}(0)=\frac{k_{\mathrm{post}}^{\mathrm{NA}}+k_{\mathrm{out}}^{\mathrm{NA}}+k_{pr}^{\mathrm{NA,cpx}}(F)+k_{\mathrm{irr}}^{\mathrm{NA}}}{\left(k_{\mathrm{post}}^{\mathrm{NA}}+k_{\mathrm{out}}^{\mathrm{NA}}\right)\left(k_{\mathrm{pre}}^{\mathrm{NA,cpx}}(F)+k_{\mathrm{irr}}^{\mathrm{NA}}\right)-k_{\mathrm{post}}^{\mathrm{NA}}k_{\mathrm{pre}}^{\mathrm{NA,cpx}}(F)},\;\text{With}\;k_{\mathrm{out}}^{\mathrm{FNA}}=k_{\mathrm{in}}^{\mathrm{SNA}}+k_{\mathrm{in}}^{\mathrm{LLP}};\;k_{\mathrm{out}}^{\mathrm{SNA}}=k_{\mathrm{in}}^{\mathrm{VSNA}}+k_{\mathrm{in}}^{\mathrm{LLP}}.$$

The kinetic rates for the RTC-nsp13.1 complex were assumed the same as for the core RTC, since the single nucleotide probabilities and timescales were also the same for these states (STAR Methods section “non-bursting elongation dynamics”).

The single nucleotide probabilities for exiting through the FNA pathway by each RTC complex without engaged nsp13-helicase cpx∈{c,1,2} were obtained as the probability to perform the irreversible step in the FNA pathway $p_{\mathrm{FNA}}^{\mathrm{cpx}}=p_{\mathrm{irr}}^{\mathrm{FNA,cpx}}$. The single nucleotide probabilities for performing the irreversible step of the slower NA∈{SNA,VSNA} pathways in each RTC complex cpx∈{c,1,2} were obtained as the recursive probability for not exiting through the irreversible step in any of the faster pathways $p_{\text{irr}}^{{\mathrm{NA}}^{\prime },\mathrm{cpx}}$ with ${\mathrm{NA}}^{\prime }\in \{\mathrm{FNA},\mathrm{SNA}\}$ times the splitting probability for entering the slower pathways $\frac{k_{\text{in}}^{\mathrm{NA}}}{k_{\text{out}}^{{\mathrm{NA}}^{\prime }}}$ times the probability of completing the irreversible step in the pathway $p_{\text{irr}}^{\mathrm{NA,cpx}}$ (Figure 4G).

(Equation 45) $$p_{\mathrm{FNA}}^{\mathrm{cpx}}(F)=p_{\mathrm{irr}}^{\mathrm{FNA,cpx}}(F),\;p_{\mathrm{SNA}}^{\mathrm{cpx}}(F)=\left(1-p_{\mathrm{irr}}^{\mathrm{FNA,cpx}}(F)\right)\frac{k_{\mathrm{in}}^{\mathrm{SNA}}}{k_{\mathrm{out}}^{\mathrm{FNA}}}p_{\mathrm{irr}}^{\mathrm{SNA},\mathrm{cpx}}(F),p_{\mathrm{VSNA}}^{\mathrm{cpx}}(F)=\left(1-p_{\mathrm{irr}}^{\mathrm{FNA,cpx}}(F)\right)\frac{k_{\mathrm{in}}^{\mathrm{SNA}}}{k_{\mathrm{out}}^{\mathrm{FNA}}}\left(1-p_{\mathrm{irr}}^{\mathrm{SNA},\mathrm{cpx}}(F)\right)\frac{k_{\mathrm{in}}^{\mathrm{VSNA}}}{k_{\mathrm{out}}^{\mathrm{SNA}}}p_{\mathrm{irr}}^{\mathrm{VSNA,cpx}},\;p_{\mathrm{LLP}}^{\mathrm{cpx}}(F)=1-p_{\mathrm{FNA}}^{\mathrm{cpx}}(F)-p_{\mathrm{SNA}}^{\mathrm{cpx}}(F)-p_{\mathrm{VSNA}}^{\mathrm{cpx}}(F),$$

With $k_{\text{out}}^{\mathrm{FNA}}=k_{\mathrm{in}}^{\mathrm{SNA}}+k_{\mathrm{in}}^{\mathrm{LLP}};k_{\text{out}}^{\mathrm{SNA}}=k_{\mathrm{in}}^{\mathrm{VSNA}}+k_{\mathrm{in}}^{\mathrm{LLP}}$. The relation for the single nucleotide probability for entering the long-lived pause $p_{\mathrm{LLP}}^{\mathrm{cpx}}$ ensures that the single nucleotide probabilities of all pathways for each RTC complex add up to 1. Note that with keeping $k_{\text{in}}^{\mathrm{LLP}}$ constant for the different non-engaged RTC-nsp13 complexes, we did not enforce that the long-lived pause probability is constant for all non-engaged RTC-nsp13 complexes, like we did in the fit of the RTC assembly – Elongation dynamics model (STAR Methods section “non-bursting elongation dynamics”), but with the global fit of the mechanochemical model we found similar values ($p_{\mathrm{LLP}}^{c}\approx p_{\mathrm{LLP}}^{2}$) (Table 1).

Translocation acts over the distance from the conversion of one base pair dsRNA to ssRNA $a=\delta x_{{\mathrm{ss}}^{-}}\delta x_{\mathrm{ds}}\approx 0.2nm$^21^ and we determined there is no tension dependence of the forward translocation rate (STAR Methods section “mechanochemistry of the non-bursting NA pathways”). Therefore, the (negative) RNA tension (*F*) dependence of the backward translocation rate could be described as the Arrhenius law with distance *a*

(Equation 46) $$k_{\text{pre}}^{\mathrm{NA,cpx}}(F)=k_{\text{pre}}^{\mathrm{NA,cpx}}(0)e^{-aF/k_{B}T},\mathrm{NA}\in \{\mathrm{FNA},\mathrm{SNA}\},\mathrm{cpx}\in \{c,1, 2\}.$$

The allosteric effect of nsp13.2 binding to the RTC was modeled as an increase in the free-energy barrier $\Delta G_{\mathrm{NA}}$ in units of $k_{B}T$ for backward translocation rate $k_{\text{pre,NA.}2}$ in each NA pathway (STAR Methods section “mechanochemistry behind the allosteric effect”).

(Equation 47) $$k_{\mathrm{pre}}^{\mathrm{NA},2}(F)=k_{\mathrm{pre}}^{\mathrm{NA},C}(F)e^{\Delta G_{\mathrm{NA}}},\mathrm{NA}\in \{\mathrm{FNA},\mathrm{SNA}\}.$$

The other rates in the mechanochemical model were unchanged when nsp13.2 is in complex with the RTC (Figure 4G).

Since the VSNA characteristic timescale was fixed for all conditions (STAR Methods section “setting parameters constant”), we modeled the pathway as a single irreversible step with probability $p_{\text{irr}}^{\text{VSNA,cpx}}$ for each RTC complex cpx∈{c,1,2} (Figure 4G). For the core RTC (cpx = c) and the RTC-nsp13.1 complex (cpx = 1) the probabilities for the irreversible step are the same $p_{\text{irr}}^{\text{VSNA,}c}=p_{\text{irr}}^{\text{VSNA,}1}$, but for the RTC-nsp13.1,2 complex the probability $p_{\text{irr}}^{\text{VSNA,2}}$ is decreased, which reflects the substantial decrease in the VSNA single nucleotide probability (Table 1) and the total VSNA probability at saturating nsp13-helicase concentration (Figure 4F).

As the long-lived pause probability was not much decreases at saturating nsp13-helicase concentration, but showed a similar trend with RNA tension as the VSNA probability (Figures 4E and 4F), We simply modeled a direct entry of the long-lived pause from the pre-translocated state of the FNA and SNA pathway with relative rate $k_{\text{in}}^{\mathrm{LLP}}$ and from the VSNA pre-translocated state with probability $\left(1-p_{\mathrm{irr}}^{\mathrm{VSNA,cpx}}\right)$. The decreased value of $p_{\mathrm{irr}}^{\mathrm{VSNA},2}$ compensated for the decrease in entry of the long-lived pause from the FNA and SNA pre-translocated state, resulting in a stable long-lived pause probability ($p_{\mathrm{LLP}}^{c}\approx p_{\mathrm{LLP}}^{2}$) (Table 1).

#### Global fit of the mechanochemical model

To perform a global fit of the mechanochemical model to the empirical dwell-time distributions, we substituted the RNA tension dependencies (Equation 46) and allosteric effects (Equation 47) into the expressions for the single nucleotide probabilities and timescales versus RNA tension (Equation 44 with Equation 45) in the RTC assembly-Elongation dynamics model (Equations 38, 39, 40, and 41), to obtain a pdf curve for each dwell-time distributions for each condition measured.

A global fit of the mechanochemical model was performed on the dwell-time distributions of the nsp13-helicase concentration dependency dataset at RNA tension *F* = 20 pN and RNA tension dependency datasets without nsp13-helicase and at saturating nsp13-helicase concentration (Figures S5N–S5P). The free parameters in the mechanochemical model were $k_{\text{in}}^{\text{SNA}},k_{\text{in}}^{\text{VSNA}},k_{\text{in}}^{\text{LLP}},k_{\text{post}}^{\text{FNA}},k_{\text{post}}^{\text{SNA}}$, $k_{\text{pre}}^{\text{FNA.c}}(0),k_{\text{pre}}^{\text{FNA.2}}(0)$, $k_{\mathrm{pre}}^{\mathrm{SNA}.c}(0),k_{\mathrm{pre}}^{\mathrm{SNA}.2}(0),k_{\mathrm{irr}}^{\mathrm{FNA}},k_{\mathrm{irr}}^{\mathrm{SNA}},p_{\mathrm{irr}}^{\mathrm{VSNA}.c},p_{\mathrm{irr}}^{\mathrm{VSNA}.2}$ with which the single nucleotide probabilities and timescales could be obtained (Equations 44, 45, 46, and 47). The substitution into the RTC assembly-Elongation dynamics model added the free-energy differences between the states in the RTC assembly model $\Delta G_{c\to 1},\Delta G_{1\to 2},\Delta G_{2\to 2^{*}}$ (Equations 39, 40, 41, and 42).

The global fit of the mechanochemical model on all datasets successfully captured the trends in the probabilities and characteristic timescales of all the pathways (Figure 3C, 3D, and 4C–4F). As expected, we obtained that for RTC-nsp13.1,2 the FNA backward translocation rate was negligible compared to the FNA forward translocation rate (Table 1). The values of the probability to perform the irreversible step in the VSNA pathway for the RTC-nsp13.1,2 complex $p_{\text{irr}}^{\text{VSNA.}2}$ were, however, much lower than for the core RTC $\left(p_{\text{irr}}^{\text{VSNA.c}}\right)$ (Table 1), while we expected it would have a similar or higher value than $p_{\text{irr}}^{\text{VSNA.c}}$ based on the allosteric effect obtained for the FNA and SNA pathway. We did not seek to find an explanation for this, because the VSNA probability was much lower than the FNA and SNA probability and the position of the corresponding shoulder was hard to determine for saturating concentration of nsp13-helicase (STAR Methods section “setting parameters constant”).

### QUANTIFICATION AND STATISTICAL ANALYSIS

The bars with error bars in the average velocity and processivity plots (Figures 1E and S3D) and the circles with error bars in the dwell-time distributions (Figures 3A, 3B, and S5) represent the mean ± standard error of the mean (SEM) from 1000 bootstraps (i.e., resamples) of the dwell-times at the specific condition. The circles (or bars) with error bars in the fit parameter plots (Figures 3C, 3D, S2, S3F, and S3G) represent the mean ± SEM from 100 bootstrap fits (i.e., fits on the resampled data) with the dwell-time fit-function (Equations 6 or 7) at the specific condition. The lines with shaded areas in the parameter plots (Figure) represent the mean ± SEM from 100 global bootstrap fits of the RTC assembly, Elongation dynamics and Mechanochemical model to the dwell-times. A change was determined to be significant if it was more than the sum of the SEMs at the compared conditions. Fits were determined to be good if they are within one SEM for most of the data bins/parameters fitted per condition. Detailed information on how the fits were performed is provided in the method details.

### ADDITIONAL RESOURCES

No additional resources were used in this study.

## Supplementary Material

1

Supplemental information can be found online at https://doi.org/10.1016/j.celrep.2026.117273.

## Figures and Tables

**Table 1.** Fit parameters of RTC assembly, elongation dynamics, and mechanochemistry model

Conditions				
[nsp13] (nM)	0-50, 0, 20	–	–	–
F (pN)	20, 20–40, 20–40	–	–	–
t_cut_ (s)	0.08	–	–	–
Value type	Best values	Mean values	SD values	Bounds
LL	−47,861	−47,4793	547	N/A
BIC	95,907	95,770	1,094	N/A
$\Delta G_{1}\left(k_{B}T\right)$	1.38	1.56	0.23	[−3, 3]
$\Delta G_{1,2}\left(k_{B}T\right)$	0.90	0.77	0.17	[−3, 3]
$\Delta G_{1,2^{*}}\left(k_{B}T\right)$	−0.72	−0.72	0.03	[−3, 3]
$k_{\text{post}}^{\text{FNA}}(1/s)$	97	104	5	[1, Inf]
$k_{\text{post}}^{\text{SNA}}(1/s)$	145	121	27	[1, Inf]
$k_{\text{pre,}0}^{\text{FNA.c}}(1/s)$	228	252	22	[1, Inf]
$k_{\text{pre,}0}^{\text{FNA.}2}(1/s)$	20	32	9	[1, Inf]
$k_{\text{pre,}0}^{\text{SNA.C}}(1/s)$	15,310	12,858	2,740	[1, Inf]
$k_{\text{pre,}0}^{\text{SNA.}2}(1/s)$	5,912	4,991	1,101	[1, Inf]
$k_{\text{in}}^{\text{SNA}}(1/s)$	3.9	3.9	0.1	[0, 10]
$k_{\text{in}}^{\text{VSNA}}(1/s)$	0.13	0.14	0.01	[0, 10]
$k_{\text{in}}^{\text{LLP}}(1/s)$	0.021	0.015	0.006	[0, 10]
$k_{\text{irr}}^{\text{FNA}}(1/s)$	40.1	39.9	0.4	[0, 100]
$k_{\text{irr}}^{\text{SNA}}(1/s)$	20.2	20.0	0.5	[0, 100]
$p_{\text{irr}}^{\text{VSNA.c}}$	1.00	0.92	0.05	[0, 1]
$p_{\text{irr}}^{\text{VSNA.}2}$	0.30	0.25	0.15	[0, 1]
$p_{\text{FNA}}^{\text{c}}\left(F_{0}\right)$	0.89	0.88	0.00	N/A
$p_{\text{FNA}}^{2}\left(F_{0}\right)$	0.95	0.95	0.00	N/A
$p_{\text{SNA}}^{c}\left(F_{0}\right)$	0.086	0.087	0.002	N/A
$p_{\text{SNA}}^{2}\left(F_{0}\right)$	0.041	0.042	0.001	N/A
$p_{\text{VSNA}}^{c}\left(F_{0}\right)$	0.022	0.022	0.001	N/A
$p_{\text{VSNA}}^{2}\left(F_{0}\right)$	0.0012	0.0011	0.0007	N/A
$p_{\text{LLP}}^{\text{c}}\left(F_{0}\right)$	0.0043	0.0049	0.0006	N/A
$p_{\text{LLP}}^{2}\left(F_{0}\right)$	0.0038	0.0040	0.0007	N/A
$\tau_{\text{VFNA}}\left(F_{0}\right)(s)$	0.02	0.02	0.00	N/A
$\tau_{\text{FNA}}^{c}\left(F_{0}\right)(s)$	0.052	0.052	0.000	N/A
$\tau_{\text{FNA}}^{2}\left(F_{0}\right)(s)$	0.036	0.037	0.000	N/A
$\tau_{\text{SNA}}^{c}\left(F_{0}\right)(s)$	1.57	1.59	0.04	N/A
$\tau_{\text{SNA}}^{2}\left(F_{0}\right)(s)$	0.74	0.75	0.04	N/A
$\tau_{\text{VSNA}}^{c}\left(F_{0}\right)(s)$	4.3	4.4	0.0	N/A
$\tau_{\text{VSNA}}^{2}\left(F_{0}\right)(s)$	4.8	4.8	0.0	N/A

t_cut_, lower dwell-time cutoff; LL, log likelihood. See also Tables S2 and S3.

**Table:** KEY RESOURCES TABLE

REAGENT or RESOURCE	SOURCE	IDENTIFIER
Antibodies		
Anti-Digoxigenin	Roche	Cat # 11333089001, RRID:AB_514496
Chemicals, peptides, and recombinant proteins		
HEPES	Carl Roth	CAS # 7365-45-9
Tris	Carl Roth	CAS # 77-86-1
Sodium Chloride	Carl Roth	CAS # 7647-14-5
Potassium Glutamate	Sigma-Aldrich	CAS # 6382-01-0
Magnesium Chloride	Carl Roth	CAS # 7791-18-6
Zink Sulfate	Sigma-Aldrich	Cat # 221376
Dextrose	Fisher	Cat # D16-1
Sodium Phosphate-monobasic	Fisher	Cat # P285-500
Sodium Phosphate-dibasic	Fisher	Cat # S375-500
Ammonium Sulfate	Fisher	Cat # A702-500
Glycerol	Fisher	Cat # G153-4
Tris (2-Carboxyethyl) phosphine Hydrochloride (TCEP)	GoldBio	Cat # TCEP1
EDTA	Carl Roth	CAS # 6381-92-6
Bovine Serum Albumin	Carl Roth	CAS # 90604-29-8
NTP	Promega	Cat # P1132
Biotin-16-UTP	Jena Bioscience	NU-821-BIO16
Digoxigenin-11-UTP	Jena Bioscience	NU-821-DIGX
RNA 5′ Polyphosphatase	Biosearch technologies	RP8092H
T4 Polynucleotide Kinase	New England Biolabs	Cat # M0201
T4 RNA ligase 2	New England Biolabs	Cat # M0239
Ulp1 peptidase	Byrd et al.^39^	N/A
Phosphocellulose (P-11)	Whatman, Sigma-Aldrich	Cat # Z753807
Agarose	Sigma-Aldrich	Cat # A9539
TAE buffer 50×	ThermoFisher	Cat # B49
TBE buffer 10×	Roth	Cat # 3061
RNA Gel Loading Dye (2X)	ThermoFisher	Cat # R0641
SyBrSafe Nucleic Acid Gel Stain	Invitrogen, ThermoFisher	Cat # S33102
The RNA storage solution	Thermo Scientific	Cat # AM7001
Strep-Tactin Superflow resin	IBA LifeSciences	Cat # 21208
Ni-NTA agarose beads	Qiagen	Cat # 36113
BioLock	IBA Lifesciences	Cat # 2-0205-050
Dithiothreitol (DTT)	ThermoFisher	Cat # R0861
isopropyl β-D-1-thiogalactopyranoside (IPTG)	Biopioneer	Cat # C0012-100
phenylmethylsulfonyl fluoride (PMSF)	Sigma-Aldrich	Cat # P7626-25G
Kanamycin sulfate	Fisher	Cat # BP906-5
chloramphenicol	Fisher	Cat # BP904-100
β-mercaptoethanol	ThermoScientific	Cat # 125472500
Desthiobiotin	IBA Lifesciences	Cat # 2-1000-002
imidazole	ThermoFisher	Cat # A10221.36
Polyethyleneimine (PEI)	Sigma-Aldrich	CAS # 26658-46-8
1% w/w TEV protease	Kirchdoerfer et al.^8^	N/A
Protease Inhibitor Tablets, EDTA-free	Pierce, ThermoFisher	Cat # A32965
NZCYM Broth	Research Products International	SKU # N72000-250.0
Critical commercial assays		
Phusion High-Fidelity PCR kit	New England Biolabs	Cat # E0553
Monarch PCR & DNA Cleanup Kit	New England Biolabs	Cat # T1030
HiScribe T7 High Yield RNA Synthesis Kit	New England Biolabs	Cat # E2040
RNA Clean & Concentrator	Zymo Research	Cat # R1015, R1017
pET-46 Ek/LIC Vector Kit	Novagen	Cat # 71335
pFastBac Dual Expression Vector	Life Technologies	Cat # 10712024
MAX Efficiency DH10Bac Competent Cells	Life Technologies	Cat # 10361012
Cellfectin II Reagent	Life Technologies	Cat # 10362100
pSUMO plasmid	LifeSensors	Cat # pE1001K
QuikChange II Site-Directed Mutagenesis Kit	Agilent Technologies	Cat # 200523
Superdex 200 Increase 10/300 GL	GE Life Sciences	Cat # GE28-9909-44
HisTrap FF column	Cytiva	Cat # 17525501
HiTrap SP Sepharos FF column	Cytiva	Cat # 17515701
Sephacyl S200-HR HiPrep 16/60 column	Cytiva	Cat # 17116601
Pierce BCA Protein Assay Kit	ThermoFisher	Cat # 23225
Deposited data		
All data obtained in this study	This study	https://doi.org/10.48338/VU01-DY3HI3
Experimental models: Cell lines		
Sf9 cells	Expression Systems	Cat # 94-001F
Sf21 cells	Expression Systems	Cat # 94-003F
Oligonucleotides		
K288A SDM- Forward Primer- GCCGGGTACCGGCGCGAGCCACTTTGCG	IDT	N/A
K288A SDM Reverse Primer - CGCAAAGTGGCTCGCGCCGGTACCCGGC	IDT	N/A
Oligo’s for dsRNA template and RNA hairpins (Table S1)	Biomers	N/A
Recombinant DNA		
pFastBac DNAs with nsp12 gene, double TEV site and strep-tag	Genscript	GenBank MN985325
nsp7 genes cloned into pET46 with an N-terminal 6× histidine tag, enterokinase site, TEV site	Novagen	GenBank MN985325
nsp8 genes cloned into pET46 with an N-terminal 6× histidine tag, enterokinase site, TEV site	Novagen	GenBank MN985325
nsp13 gene from SARS-CoV-2 Washington isolate as *E. coli* codon-optimized fragment	GenScript	GenBank MN985325
pBAD/Myc-HisB plasmid (Invitrogen) with *mukB-His10*	Papini et al.^40^	N/A
pMK-T (ColE1 ori, KanR) plasmid (ThermoFisher) with a palindromic sequence	Papini et al.^40^	N/A
Software and algorithms		
Python 3	Python Software Foundation	https://www.python.org/download/releases/3.0/
labVIEW	National Instruments^41^	https://www.ni.com/en-us.html
Bead-tracking software in labVIEW	Cnossen et al.^42^	http://www.github.com/jcnossen/BeadTracker
Data analysis code	This study	https://doi.org/10.48338/VU01-DY3HI3
Other		
3130XL Genetic Analyzer	Applied Biosystems	Cat # 106MI60-01
Akta Pure 25 M FPLC	Cytiva	29018226
Amicon Ultra-15 centrifugation filter	MilliporeSigma	Cat # UFC9010D
Centrifugal concentrator	Vivaspin	Cat # VS0404
Microfluidizer	Microfluidics	Cat # LM20
Dynabeads M-270 Streptavidin	Invitrogen	Cat # 65305
Polystyrene beads	Sigma-Aldrich	SKU # LB3-1ML
50× oil immersion objective lens	Nikon	Cat # MRL01502
CMOS camera	Teledyne DALSA Falcon2	Cat # FA-80-12M1H
Opto-mechanics	Thorlabs	https://www.thorlabs.de/
Tube lens	Qioptic	Cat # G322304000
Piezo	Physic Instrument (PI)	Cat # P-726.1CD
Motors (z-position, rotation)	Physic Instrument (PI)	Cat # M-126-PD1, C-150
Pump	Cole-Parmer GmbH	Cat # 77122-32
LED	OSRAM Opto	GTIN # 4062986000272
Permanent magnets	Supermagnete	Cat # W-05-G
#1 Coverglass	OMNILAB, Menzel	Cat # 1184900
Foil heater	Thorlabs	Cat # HT10K
Kapton tape	Thorlabs	Cat # KAP22-075
PID temperature controller	Thorlabs	Cat # TC200
GPU	NVIDIA	Cat # GK104-355-A2

## Data Availability

• All data have been deposited at the Data Publication Platform of Vrije Universiteit Amsterdam and are publicly available as of the date of publication at https://doi.org/10.48338/VU01-DY3HI3. • All original code has been deposited at the Data Publication Platform of Vrije Universiteit Amsterdam and is publicly available at https://doi.org/10.48338/VU01-DY3HI3 as of the date of publication. • Any additional information required to reanalyze the data reported in this paper is available from the lead contact upon request.

## Highlights

- Single-molecule tweezers show CoV helicase boosts duplex RNA synthesis 10-fold
- The helicase and polymerase form a complex, enabling very fast nucleotide addition
- The helicase coordinates elongation by engaging with the non-template strand
- Kinetic modeling reveals helicase-controlled fast and slow modes of elongation

## References

[1] Lehmann KC, Snijder EJ, Posthuma CC, and Gorbalenya AE (2015). What we know but do not understand about nidovirus helicases. Virus Res. 202, 12–32. 10.1016/j.virusres.2014.12.001. 25497126 PMC7114383

[2] Gorbalenya AE, and Koonin EV (1989). Viral proteins containing the purine NTP-binding sequence pattern. Nucleic Acids Res. 17, 8413–8440. 10.1093/nar/17.21.8413. 2555771 PMC335016

[3] Marecki JC, Belachew B, Gao J, and Raney KD (2021). RNA helicases required for viral propagation in humans. Enzymes 50, 335–367. 10.1016/bs.enz.2021.09.005. 34861942 PMC8562938

[4] Chen J, Malone B, Llewellyn E, Grasso M, Shelton PMM, Olinares PDB, Maruthi K, Eng ET, Vatandaslar H, Chait BT, (2020). Structural Basis for Helicase-Polymerase Coupling in the SARS-CoV-2 Replication-Transcription Complex. Cell 182, 1560–1573.e13. 10.1016/j.cell.2020.07.033. 32783916 PMC7386476

[5] Osawa T, Aoki M, Ehara H, and Sekine SI (2023). Structures of dengue virus RNA replicase complexes. Mol. Cell 83, 2781–2791.e4. 10.1016/j.molcel.2023.06.023. 37478848

[6] Tan YB, Chmielewski D, Law MCY, Zhang K, He Y, Chen M, Jin J, and Luo D (2022). Molecular architecture of the Chikungunya virus replication complex. Sci. Adv 8, eadd2536. 10.1126/sciadv.add2536. 36449616 PMC9710867

[7] Hillen HS, Kokic G, Farnung L, Dienemann C, Tegunov D, and Cramer P (2020). Structure of replicating SARS-CoV-2 polymerase. Nature 584, 154–156. 10.1038/s41586-020-2368-8. 32438371

[8] Kirchdoerfer RN, and Ward AB (2019). Structure of the SARS-CoV nsp12 polymerase bound to nsp7 and nsp8 co-factors. Nat. Commun. 10, 2342. 10.1038/s41467-019-10280-3. 31138817 PMC6538669

[9] Hillen HS (2021). Structure and function of SARS-CoV-2 polymerase. Curr. Opin. Virol 48, 82–90. 10.1016/j.coviro.2021.03.010. 33945951 PMC8023233

[10] Yan L, Zhang Y, Ge J, Zheng L, Gao Y, Wang T, Jia Z, Wang H, Huang Y, Li M, (2020). Architecture of a SARS-CoV-2 mini replication and transcription complex. Nat. Commun 11, 5874. 10.1038/s41467-020-19770-1. 33208736 PMC7675986

[11] Grimes SL, and Denison MR (2024). The Coronavirus helicase in replication. Virus Res. 346, 199401. 10.1016/j.virusres.2024.199401. 38796132 PMC11177069

[12] Newman JA, Douangamath A, Yadzani S, Yosaatmadja Y, Aimon A, Brandão-Neto J, Dunnett L, Gorrie-Stone T, Skyner R, Fearon D, (2021). Structure, mechanism and crystallographic fragment screening of the SARS-CoV-2 NSP13 helicase. Nat. Commun 12, 4848. 10.1038/s41467-021-25166-6. 34381037 PMC8358061

[13] Mickolajczyk KJ, Shelton PMM, Grasso M, Cao X, Warrington SE, Aher A, Liu S, and Kapoor TM (2021). Force-dependent stimulation of RNA unwinding by SARS-CoV-2 nsp13 helicase. Biophys. J 120, 1020–1030. 10.1016/j.bpj.2020.11.2276. 33340543 PMC7837305

[14] Marx SK, Mickolajczyk KJ, Craig JM, Thomas CA, Pfeffer AM, Abell SJ, Carrasco JD, Franzi MC, Huang JR, Kim HC, (2023). Observing inhibition of the SARS-CoV-2 helicase at single-nucleotide resolution. Nucleic Acids Res. 51, 9266–9278. 10.1093/nar/gkad660. 37560916 PMC10516658

[15] Chen J, Wang Q, Malone B, Llewellyn E, Pechersky Y, Maruthi K, Eng ET, Perry JK, Campbell EA, Shaw DE, and Darst SA (2022). Ensemble cryo-EM reveals conformational states of the nsp13 helicase in the SARS-CoV-2 helicase replication-transcription complex. Nat. Struct. Mol. Biol 29, 250–260. 10.1038/s41594-022-00734-6. 35260847 PMC8935131

[16] Bera SC, Seifert M, Kirchdoerfer RN, Van Nies P, Wubulikasimu Y, Quack S, Papini FS, Arnold JJ, Canard B, Cameron CE, (2021). The nucleotide addition cycle of the SARS-CoV-2 polymerase. Cell Rep. 36, 109650. 10.1016/j.celrep.2021.109650. 34433083 PMC8367775

[17] Manfredonia I, Nithin C, Ponce-Salvatierra A, Ghosh P, Wirecki TK, Marinus T, Ogando NS, Snijder EJ, van Hemert MJ, Bujnicki JM, and Incarnato D (2020). Genome-wide mapping of SARS-CoV-2 RNA structures identifies therapeutically-relevant elements. Nucleic Acids Res. 48, 12436–12452. 10.1093/nar/gkaa1053. 33166999 PMC7736786

[18] Seifert M, van Nies P, Papini FS, Arnold JJ, Poranen MM, Cameron CE, Depken M, and Dulin D (2020). Temperature controlled high-throughput magnetic tweezers show striking difference in activation energies of replicating viral RNA-dependent RNA polymerases. Nucleic Acids Res. 48, 5591–5602. 10.1093/nar/gkaa233. 32286652 PMC7261197

[19] Dulin D, Arnold JJ, Van Laar T, Oh H-S, Lee C, Perkins AL, Harki DA, Depken M, Cameron CE, and Dekker NH (2017). Signatures of Nucleotide Analog Incorporation by an RNA-Dependent RNA Polymerase Revealed Using High-Throughput Magnetic Tweezers. Cell Rep. 21, 1063–1076. 10.1016/j.celrep.2017.10.005. 29069588 PMC5670035

[20] Dulin D (2024). An Introduction to Magnetic Tweezers. In Single Molecule Analysis : Methods and Protocols, Heller I, Dulin D, and Peterman EJG, eds. (Springer US), pp. 375–401. 10.1007/978-1-0716-3377-9_18 37824014

[21] Dulin D, Vilfan ID, Berghuis BA, Hage S, Bamford DH, Poranen MM, Depken M, and Dekker NH (2015). Elongation-Competent Pauses Govern the Fidelity of a Viral RNA-Dependent RNA Polymerase. Cell Rep. 10, 983–992. 10.1016/j.celrep.2015.01.031. 25683720

[22] Ostrofet E, Papini FS, and Dulin D (2018). Correction-free force calibration for magnetic tweezers experiments. Sci. Rep 8, 15920. 10.1038/s41598-018-34360-4. 30374099 PMC6206022

[23] Quack S, and Dulin D (2024). Surface Functionalization, Nucleic Acid Tether Characterization, and Force Calibration for a Magnetic Tweezers Assay. In Single Molecule Analysis : Methods and Protocols, Heller I, Dulin D, and Peterman EJG, eds. (Springer US), pp. 403–420. 10.1007/978-1-0716-3377-9_19. 37824015

[24] Seifert M, Bera SC, Van Nies P, Kirchdoerfer RN, Shannon A, Le T-T-N, Meng X, Xia H, Wood JM, Harris LD, (2021). Inhibition of SARS-CoV-2 polymerase by nucleotide analogs from a single-molecule perspective. eLife 10, e70968. 10.7554/eLife.70968. 34617885 PMC8497053

[25] Dulin D, Berghuis BA, Depken M, and Dekker NH (2015). Untangling reaction pathways through modern approaches to high-throughput single-molecule force-spectroscopy experiments. Curr. Opin. Struct. Biol 34, 116–122. 10.1016/j.sbi.2015.08.007. 26434413

[26] Klein M, Das A, Bera SC, Anderson TK, Kocincova D, Lee HW, Wang B, Papini FS, Marecki JC, Arnold JJ, (2025). A post-assembly conformational change makes the SARS-CoV-2 polymerase elongation-competent. Nucleic Acids Res. 53, gkaf450. 10.1093/nar/gkaf450. 40464687 PMC12135201

[27] Gao Y, Yan L, Huang Y, Liu F, Zhao Y, Cao L, Wang T, Sun Q, Ming Z, Zhang L, (2020). Structure of the RNA-dependent RNA polymerase from COVID-19 virus. Science 368, 779–782. 10.1126/science.abb7498. 32277040 PMC7164392

[28] Wolff G, Limpens RWAL, Zevenhoven-Dobbe JC, Laugks U, Zheng S, de Jong AWM, Koning RI, Agard DA, Grünewald K, Koster AJ, (2020). A molecular pore spans the double membrane of the coronavirus replication organelle. Science 369, 1395–1398. 10.1126/science.abd3629. 32763915 PMC7665310

[29] Klein S, Cortese M, Winter SL, Wachsmuth-Melm M, Neufeldt CJ, Cerikan B, Stanifer ML, Boulant S, Bartenschlager R, and Chlanda P (2020). SARS-CoV-2 structure and replication characterized by in situ cryo-electron tomography. Nat. Commun 11, 5885. 10.1038/s41467-020-19619-7. 33208793 PMC7676268

[30] Sawicki SG, Sawicki DL, and Siddell SG (2007). A contemporary view of coronavirus transcription. J. Virol 81, 20–29. 10.1128/JVI.01358-06. 16928755 PMC1797243

[31] Depken M, Galburt EA, and Grill SW (2009). The Origin of Short Transcriptional Pauses. Biophys. J 96, 2189–2193. 10.1016/j.bpj.2008.12.3918. 19289045 PMC2717293

[32] English BP, Min W, van Oijen AM, Lee KT, Luo G, Sun H, Cherayil BJ, Kou SC, and Xie XS (2006). Ever-fluctuating single enzyme molecules: Michaelis-Menten equation revisited. Nat. Chem. Biol 2, 87–94. 10.1038/nchembio759. 16415859

[33] Malone B, Urakova N, Snijder EJ, and Campbell EA (2022). Structures and functions of coronavirus replication-transcription complexes and their relevance for SARS-CoV-2 drug design. Nat. Rev. Mol. Cell Biol 23, 21–39. 10.1038/s41580-021-00432-z. 34824452 PMC8613731

[34] Agostini ML, Andres EL, Sims AC, Graham RL, Sheahan TP, Lu X, Smith EC, Case JB, Feng JY, Jordan R, (2018). Coronavirus Susceptibility to the Antiviral Remdesivir (GS-5734) Is Mediated by the Viral Polymerase and the Proofreading Exoribonuclease. mBio 9, e00221–18. 10.1128/mBio.00221-18. 29511076 PMC5844999

[35] Gordon CJ, Tchesnokov EP, Woolner E, Perry JK, Feng JY, Porter DP, and Götte M (2020). Remdesivir is a direct-acting antiviral that inhibits RNA-dependent RNA polymerase from severe acute respiratory syndrome coronavirus 2 with high potency. J. Biol. Chem 295, 6785–6797. 10.1074/jbc.RA120.013679. 32284326 PMC7242698

[36] Bravo JPK, Dangerfield TL, Taylor DW, and Johnson KA (2021). Remdesivir is a delayed translocation inhibitor of SARS-CoV-2 replication. Mol. Cell 81, 1548–1552.e4. 10.1016/j.molcel.2021.01.035. 33631104 PMC7843106

[37] Kokic G, Hillen HS, Tegunov D, Dienemann C, Seitz F, Schmitzova J, Farnung L, Siewert A, Höbartner C, and Cramer P (2021). Mechanism of SARS-CoV-2 polymerase stalling by remdesivir. Nat. Commun 12, 279. 10.1038/s41467-020-20542-0. 33436624 PMC7804290

[38] Sama B, Selisko B, Falcou C, Fattorini V, Piorkowski G, Touret F, Donckers K, Neyts J, Jochmans D, Shannon A, (2024). The effects of Remdesivir’s functional groups on its antiviral potency and resistance against the SARS-CoV-2 polymerase. Antivir. Res 232, 106034. 10.1016/j.antiviral.2024.106034. 39510431

[39] Byrd AK, Malone EG, Hazeslip L, Zafar MK, Harrison DK, Thompson MD, Gao J, Perumal SK, Marecki JC, and Raney KD (2022). A structural feature of Dda helicase which enhances displacement of streptavidin and trp repressor from DNA. Protein Sci. 31, 407–421. 10.1002/pro.4232. 34761452 PMC8819844

[40] Papini FS, Seifert M, and Dulin D (2019). High-yield fabrication of DNA and RNA constructs for single molecule force and torque spectroscopy experiments. Nucleic Acids Res. 47, e144. 10.1093/nar/gkz851. 31584079 PMC6902051

[41] Bitter R, Mohiuddin T, and Nawrocki M (2017). LabView: Advanced Programming Techniques, Second Edition 2nd ed. (CRC Press). 10.1201/9780849333255.

[42] Cnossen JP, Dulin D, and Dekker NH (2014). An optimized software framework for real-time, high-throughput tracking of spherical beads. Rev. Sci. Instrum 85, 103712. 10.1063/1.4898178. 25362408

[43] Gohara DW, Ha CS, Kumar S, Ghosh B, Arnold JJ, Wisniewski TJ, and Cameron CE (1999). Production of “Authentic” Poliovirus RNA-Dependent RNA Polymerase (3Dpol) by Ubiquitin–Protease-Mediated Cleavage in *Escherichia coli*. Protein Expr. Purif 17, 128–138. 10.1006/prep.1999.1100. 10497078

[44] Arnold JJ, Bernal A, Uche U, Sterner DE, Butt TR, Cameron CE, and Mattern MR (2006). Small ubiquitin-like modifying protein isopeptidase assay based on poliovirus RNA polymerase activity. Anal. Biochem 350, 214–221. 10.1016/j.ab.2005.11.001. 16356462 PMC2094218

[45] Konishi S, and Kitagawa G (2008). Information Criteria and Statistical Modeling (Springer). 10.1007/978-0-387-71887-3.

[46] Phillips R, Kondev J, Theriot J, Garcia HG, and Orme N (2012). Physical Biology of the Cell, 2nd ed. (Garland Science). 10.1201/9781134111589.

[47] Palani S (2022). Signals and Systems (Springer International Publishing). 10.1007/978-3-030-75742-7.

[48] Grossman-Haham I, Rosenblum G, Namani T, and Hofmann H (2018). Slow domain reconfiguration causes power-law kinetics in a two-state enzyme. Proc. Natl. Acad. Sci 115, 513–518. 10.1073/pnas.1714401115. 29298911 PMC5776979

