# Supplementary material for: RNA virus polymerase-helicase coupling enables rapid elongation through duplex RNA
Source: Cell Rep. Author manuscript; Available in PMC 2026 May 21. (PMC13190607; doi:10.1016/j.celrep.2026.117273)
Supplement: 1 [file NIHMS2170806-supplement-1.pdf]

Cell Reports, Volume *45*

Supplemental information

RNA virus polymerase-helicase coupling enables rapid elongation through duplex RNA

Pim P.B. America, Subhas C. Bera, Arnab Das, Thomas K. Anderson, John C. Marecki, Flávia S. Papini, Jamie J. Arnold, Robert N. Kirchdoerfer, Craig E. Cameron, Kevin D. Raney, Martin Depken, and David Dulin

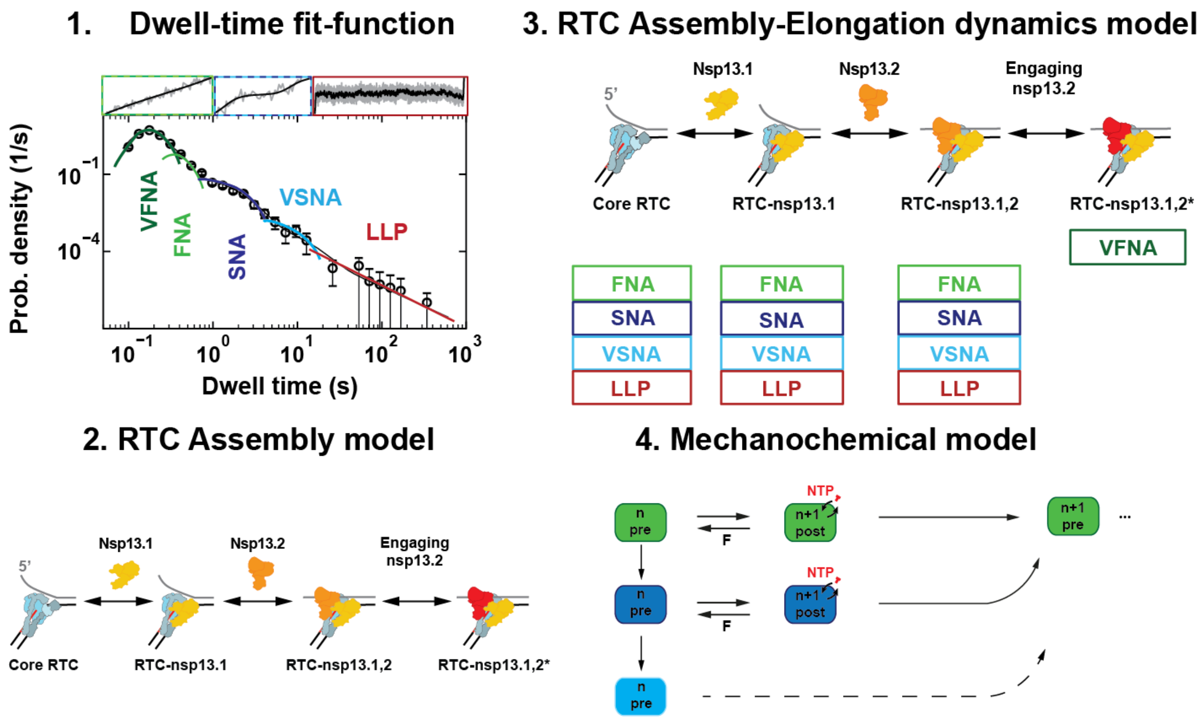

Figure S1. Schematic for the build up of the complete model describing elongation dynamics by the core RTC and RTC-nsp13 complexes on dsRNA. (1.) To extract information from the dwell-time distributions for building our microscopic models, we constructed a dwell-time fit-function consisting of two peaks, representing very fast and fast nucleotide addition (VFNA and FNA), two shoulders, representing slow and very slow nucleotide addition (SNA and VSNA) and a tail of long dwell-times representing long-lived pause recovery (LLP). **(2.)** For RTC-nsp13 complex formation we derived an RTC Assembly model from comparison of elongation dynamics by the SARS-CoV-2 RTC with or without nsp13-helicase on single- or double-stranded RNA and structural studies. **(3.)** By connecting our observations on the elongation dynamics from the empirical dwell-time distributions versus nsp13-helicase concentration to the RTC Assembly model we derived the RTC Assembly-Elongation dynamics model. **(4.)** From the trends observed in the RNA tension dependency of the elongation dynamics for the core RTC and at saturating nsp13-helicase concentration we determine the underlying mechanochemical model. Related to **Figure 3** and **Figure 4**.

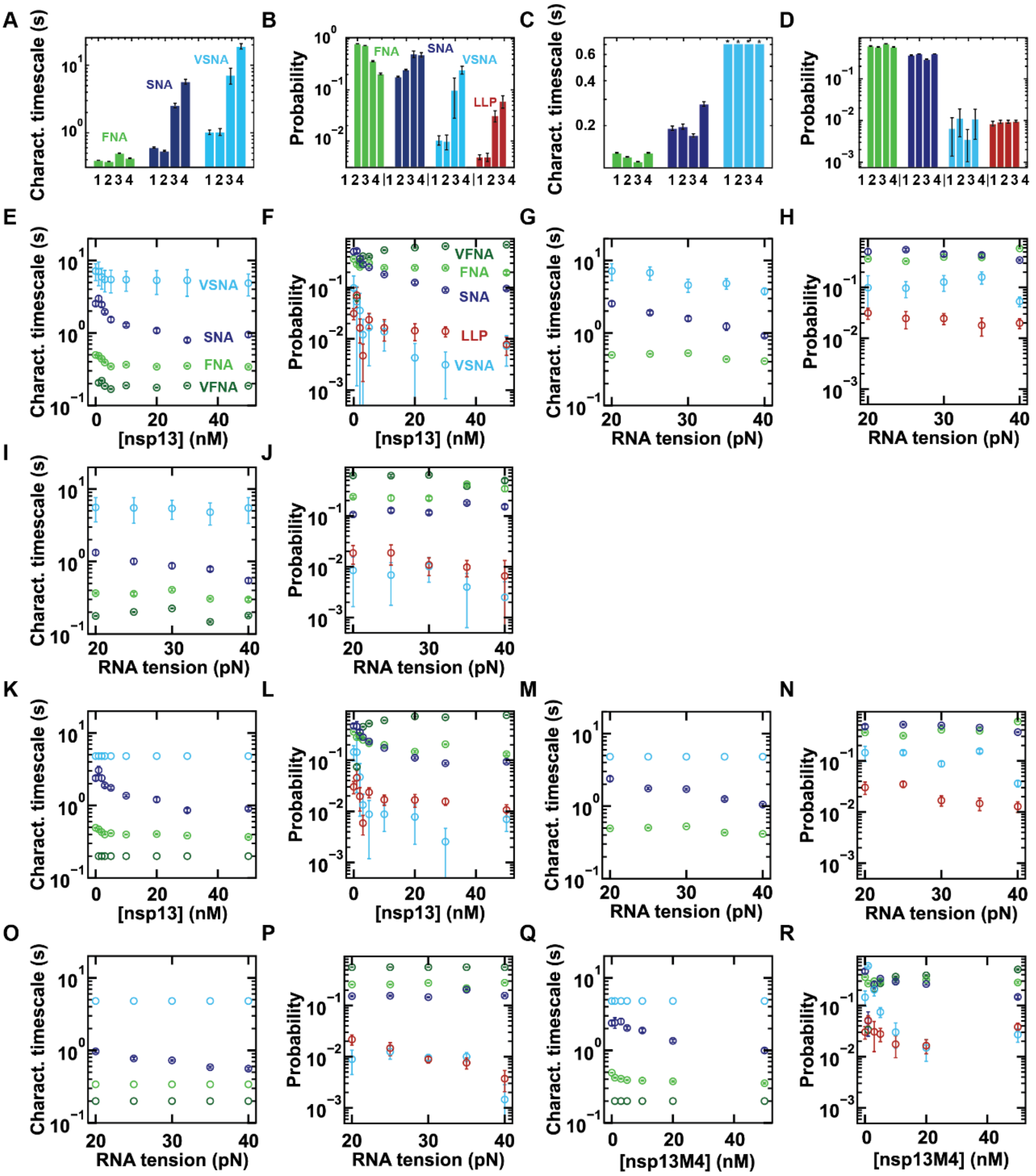

Figure S2. Parameters from the dwell-time fits with all parameters free or the VFNA and VSNA characteristic timescales fixed for RTC elongation dynamics. (A-R) Characteristic timescales (s) and probabilities for RTC elongation dynamics versus nsp13-helicase concentration (nM) from the dwell-time fits. Parameters for the VFNA, FNA, SNA, VSNA or LLP are shown in dark green, light green, dark blue, light blue or red, respectively. (A, B) Parameter bar plots for Poliovirus RdRp (1, 2) or SARS-CoV-2 core RTC (3, 4) elongating on a dsRNA template with wild-type nsp13-helicase (nsp13) (2) or ATPase dead nsp13-helicase mutant (nsp13D) (4), respectively. (C, D) Parameter bar plots for elongation dynamics by core RTC with increasing nsp13-helicase concentration (0, 2 and 20 nM in 1, 2, and 3) or saturating nsp13D concentration (20 nM in 4) on an ssRNA template. The bars and error bars are the mean ± SEM for each condition. (*) VSNA characteristic timescale is fixed in the fits. (E-P) Parameters for elongation dynamics by the core RTC with nsp13-helicase on dsRNA from dwell-time fits with all parameters free (E-J) or the VSNA and VFNA characteristic timescales fixed to 0.2 s and 4.8 s, respectively (K, M and Q, no error bars). (E, F, K, L) Parameters for increasing nsp13-helicase concentration ([nsp13] (nM)), (G, H, M, N) for the core RTC with RNA tension (pN), (I, J, O, P) for saturating nsp13-helicase concentration (20 nM) with increasing RNA tension (pN) or (Q, R) for increasing nsp13M4 concentration (nM) or (Q, R) for increasing nsp13M4 concentration. The circles with error bars represent the mean ± SEM from bootstrap fits. Related to **Figure 3** and **Figure 4**.

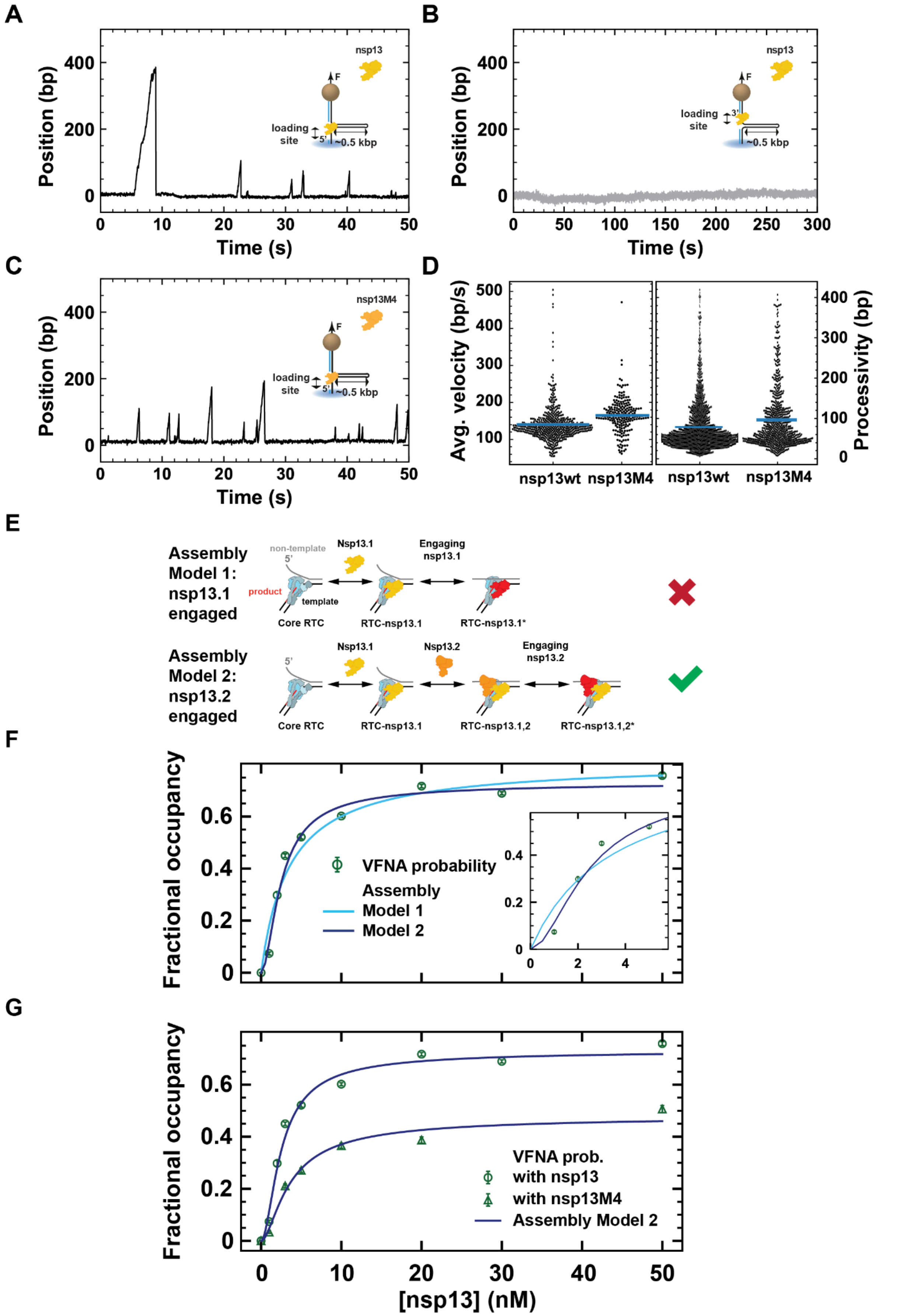

Figure S3. Nsp13-helicase unwinds dsRNA in the 5' to 3' direction and nsp13.2 assists RTC elongation through engagement with the non-template RNA. (A) Example trace of nsp13-helicase

(20 nM) unwinding dsRNA from a 5’-end ssRNA loading site at 18 pN assisting force. **(B)** Test for dsRNA unwinding activity by nsp13-helicase (20 nM) from 3’-end ssRNA loading site at 18 pN assisting force. **(C)** Example trace of nsp13M4 (20 nM) unwinding dsRNA with 5’-end ssRNA loading site. Schematics of the hairpin assays are given in the insets of A, B and C. The measurements for A, B and C were performed with 18 pN tension on the RNA. **(D)** Average velocity (bp/s) (left) and processivity (bp) (right) for each unwinding event with 20 nM nsp13-helicase or nsp13M4 compared on the RNA hairpin with 5’-end loading site (A, C). The average velocity was calculated for unwinding events longer than 100 bp. **(E)** Schematic representation of the assembly models tested. In Assembly model 1, nsp13.1 binds first to the RTC (RTC-nsp13.1) and engages with the non-template RNA (RTC-nsp13.1*) making the very fast nucleotide addition pathway. In Assembly model 2, nsp13.1 binds first, followed by nsp13.2 which engages with the non-template RNA (RTC-nsp13.1,2*). **(F)** Fits with the nsp13-engaged state fractional occupancy to the VFNA probability versus nsp13-helicase concentration ([nsp13] in nM) for the two assembly models. Fractional occupancy of RTC-nsp13.1* (sky blue line) or RTC-nsp13.1,2* (dark blue line) versus [nsp13] for Assembly model 1 or 2, respectively. (green circles with error bars) The mean ± SEM of the VFNA probability. **(G)** Fits with the fractional occupancy of the nsp13-engaged state (dark blue lines) to the VFNA probability versus either nsp13-helicase or nsp13M4 concentration for Assembly model 2 (green circles or triangles with error bars). The mean ± SEM of the VFNA probability obtained from 100 dwell-time bootstrap fits versus nsp13-helicase concentration with fixed VFNA and VSNA characteristic timescale. Related to **Figure 2** and **Figure 3E-G**.

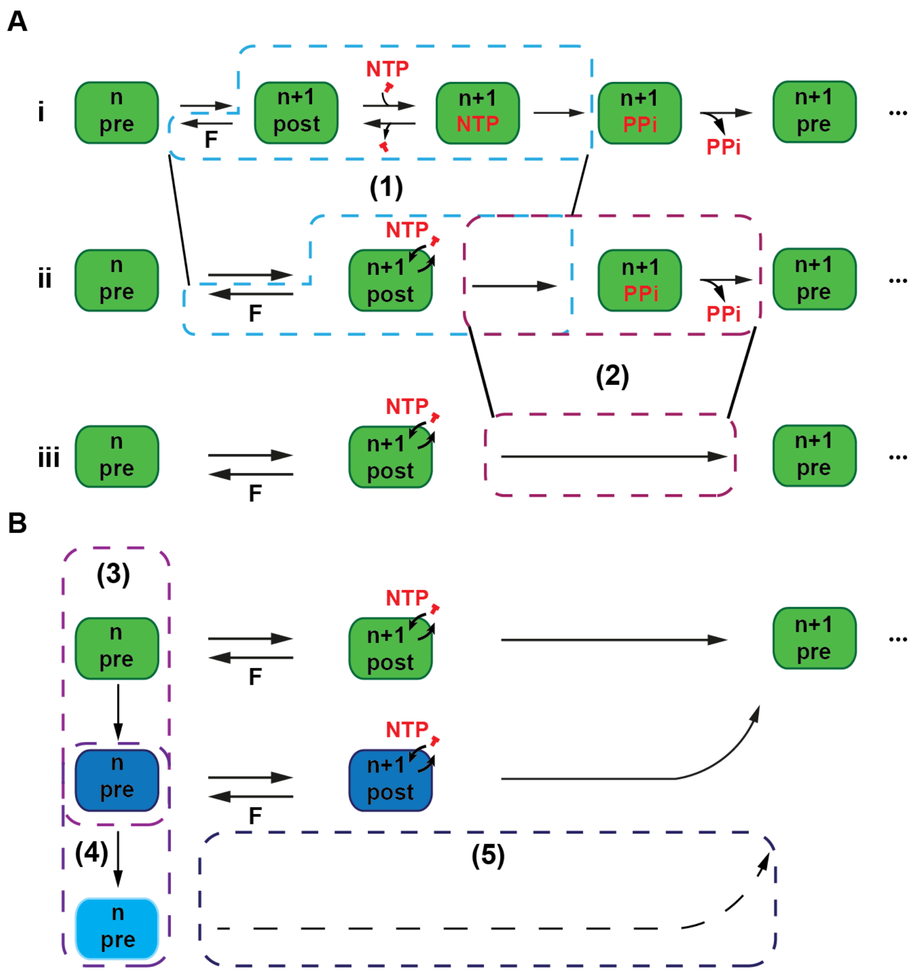

Figure S4. Simplification and connections made in the process of building the mechanochemical model for the SARS-CoV-2 RTC elongation dynamics on a dsRNA template. (A) We started from a general mechanochemical model for the NA cycle (i) including translocation, nucleotide (NTP) binding, catalysis, and pyrophosphate (PPi) release (first line). Based on Bera et al. 2021, the tension (F) dependency is on the backward translocation rate. We determined that the translocation cycle is rate-limiting, therefore (1) (un)binding of NTPs is assumed in rapid equilibrium (ii) and (2) NTP catalysis and PPi release are considered as a single irreversible step (iii), which is fast compared to the characteristic timescale of the NA cycle. **(B)** From comparison of the NA pathway probabilities versus RNA tension for the core RTC we determined that (3) The SNA pathway (dark blue) is entered from the FNA pre-translocated state. (4) The VSNA pathway (sky blue) is entered from the SNA pre-translocated state. (5) Since we could not determine the mechanochemistry behind the VSNA characteristic timescale, we modelled the pathway with a probability to perform the irreversible step. Related to **Figure 4G**.

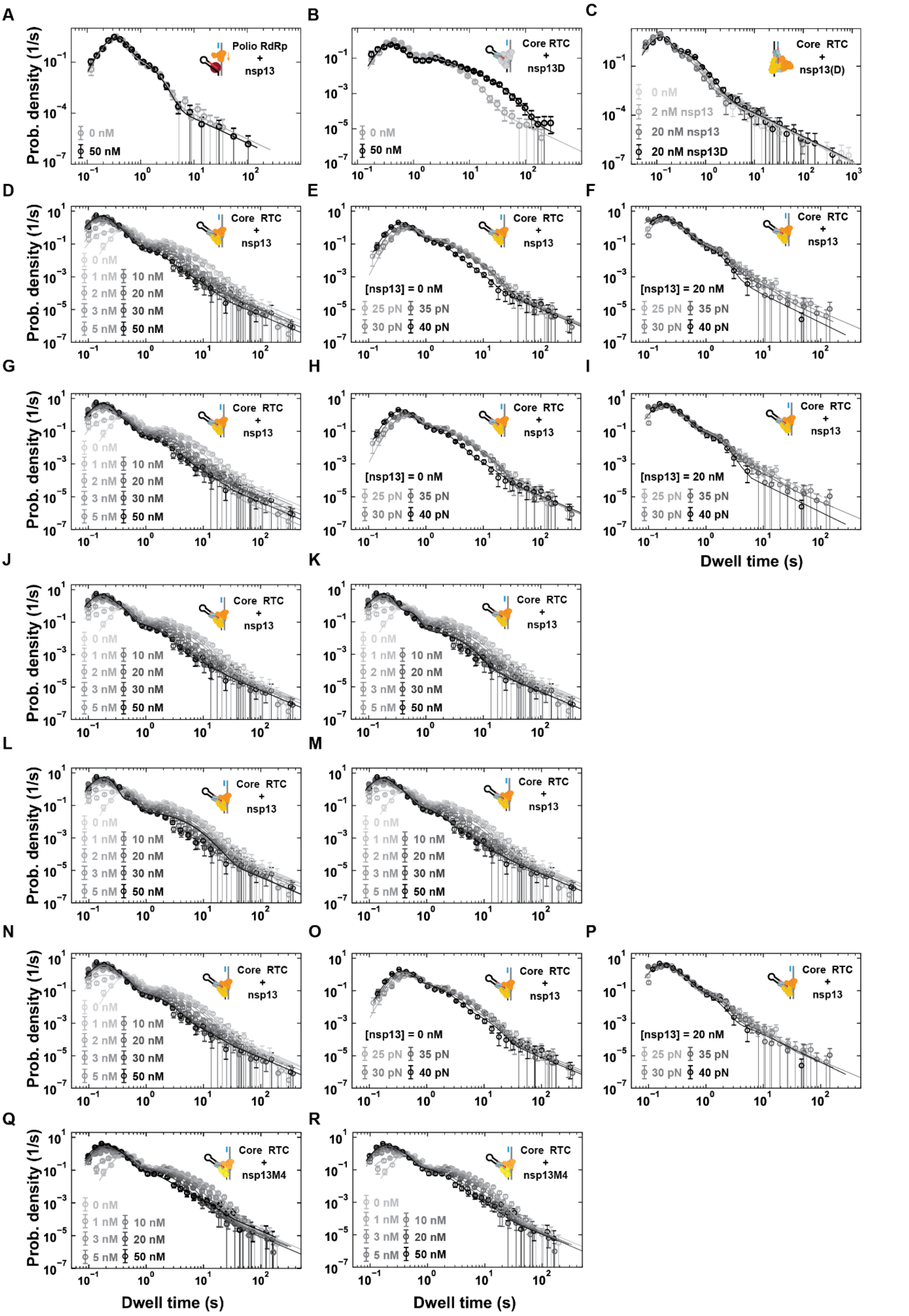

Figure S5. Fits for RTC elongation dynamics with or without wild-type or mutant nsp13-helicase on the dwell-time distributions. (A-R) Dwell-time distributions (circles with error bars) with fit curves (lines) for the RTC elongation dynamics at different conditions with or without nsp13-helicase or mutants

on a ss- or dsRNA template. (A, B) Dwell-time fits for RTC elongation dynamics are shown with (black) or without (grey) saturating concentrations of nsp13-helicase (50 nM) for either (A) Poliovirus RdRp or (B) SARS-CoV-2 core RTC elongating on a dsRNA template with wild-type nsp13-helicase (nsp13) (A) or ATPase dead nsp13 (nsp13D) (B) respectively. (C) Dwell-time distributions for elongation dynamics on ssRNA for the core RTC with increasing nsp13-helicase concentration (0 nM, 2 nM, 20 nM) or saturating nsp13D concentration (20 nM) at constant RNA tension (25 pN). (D, E, F) Dwell-time fits with all parameters free for RTC elongation dynamics on a dsRNA template with nsp13-helicase. (G, H, I) Dwell-time fits with the VFNA and VSNA characteristic timescale fixed to 0.2 s and 4.8 s respectively for RTC elongation dynamics on a dsRNA template with nsp13-helicase. (J, K, L) Fit of RTC assembly - Elongation dynamics model on the dwell-times versus nsp13-helicase concentration with all parameters free for elongation by the RTC-nsp13.1,2 complex (J), with only the probabilities of the pathways free for the RTC-nsp13.1,2 (K) and with all elongation parameters fixed for the values of the core RTC (L). (M) Fit curves without timescale averaging of the RTC assembly - Elongation dynamics model with the parameters from the fit shown in (J). (N, O, P) Global fit of the combination of the RTC assembly, elongation dynamics and the mechanochemical model for RTC elongation on a dsRNA template with or without nsp13-helicase. (Q) Dwell-time fits with the VSNA and VFNA characteristic timescale fixed for increasing nsp13M4 concentration. (R) Fit of RTC assembly - Elongation dynamics model on the dwell-times versus nsp13M4 concentration with all parameters free for elongation by the RTC-nsp13.1,2 complex. Fit curves on top of the dwell-time distributions versus nsp13-helicase concentration at 20 pN RNA tension (D, G, J-N, Q-R). Fit curves on the dwell-time distributions versus RNA tension without nsp13-helicase (E, H, O) or at 20 nM nsp13-helicase (F, I, P). The dwell-time distributions are divided into 30 bins, zero value bins are removed and the remaining bins are centered again. The circles and error bars represent the mean ± SEM for every bin obtained from 1000 bootstraps of the dwell-times. Related to **Figure 3** and **Figure 4**.

Table S1. RNA constructs used to study the RTC and nsp13-helicase unwinding activity. (Top) Double-stranded (ds) RNA with a small hairpin with a 3'-end single-stranded (ss) RNA loading site for the RTC to bind and start RNA synthesis. **(Middle)** RNA hairpin with a 3'-end ssRNA loading site. **(Bottom)** RNA hairpin with a 5'-end ssRNA loading site.

Construct	Oligo's
dsRNA template<br>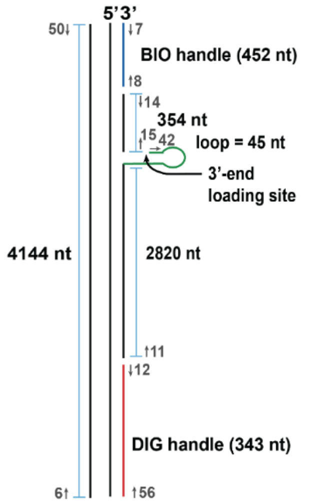 	Primer # 6 - GGTTAACCTCAACTTCCATTTCC
	Primer # 7 - AAGATTAGCGGATCCTACCTGAC
	Primer # 8 - TAATACGACTCACTATAGGAACGAGCTTGATATCCACTTTACG
	Primer # 11 - TAATACGACTCACTATAGGCCGGACGTTTCGGATCTTCCGACATGCGC
	Primer # 12 - AGCGTAAAATTCAGTTCTTCGTGGCG
	Primer # 14 - TGCCATTCAGGGACTGCCGATGTCGGTGCAGCCG
	Primer # 15 - TAATACGACTCACTATAGGAGCGCCGCTTCCATGTCCTGGAACGCT
	Primer # 42 - ACGCTTTCGCGTACACCAACAGTTAAAACCGTATGACGCTGGAAG
	Primer # 50 - TAATACGACTCACTATAGGAAGATTAGCGGATCCTACCTGAC
	Primer # 56 - TAATACGACTCACTATAGGGGTTAACCTCAACTTCCATTTCC
	pBAD/Myc-HisB plasmid (Invitrogen) with mukB-His10
RNA hairpin with 3'-end loading site<br>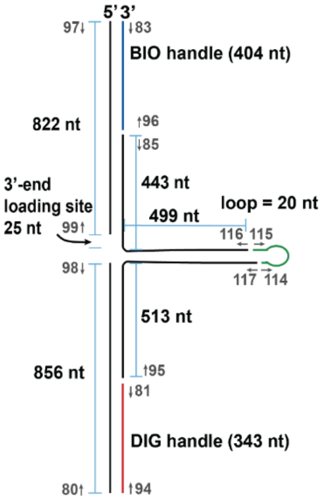 	Primer # 80 - CCTCACTTCTGCTATTTCGC
	Primer # 81 - GCGACTTAGCTGAGGCC
	Primer # 83 - GGAACCAAAGGATATTCAGACG
	Primer # 85 - GGTGCCCACAGAACGTC
	Primer # 94 - TAATACGACTCACTATAGGCCTCACTTCTGCTATTTCGC
	Primer # 95 - TAATACGACTCACTATAGGTCTTCGCCAGACGGCATTTA
	Primer # 96 - TAATACGACTCACTATAGGGCAGGCAAGTCCGATTTTTTG
	Primer # 97 - TAATACGACTCACTATAGGGGAACCAAAGGATATTCAGACG
	Primer # 98 - TAATACGACTCACTATAGGTGCTGAGGAACCGGAGTG
	Primer # 99 - AACAAGAAACTTCCTTGGCTG
	Primer # 114 - TAATACGACTCACTATAGGGTAGTGATTAACATTCGACAGCATGCGCAC
	Primer # 115 - CAGGATCACGTTACCGCC
	Primer # 116 - TAATACGACTCACTATAGGAACGGCGCCTATGACG
	Primer # 117 - TGGATCCGTGGGCGC
	pMK-T (ColE1 ori, KanR) plasmid (ThermoFisher) with a palindromic sequence
RNA hairpin with 5'-end loading site<br>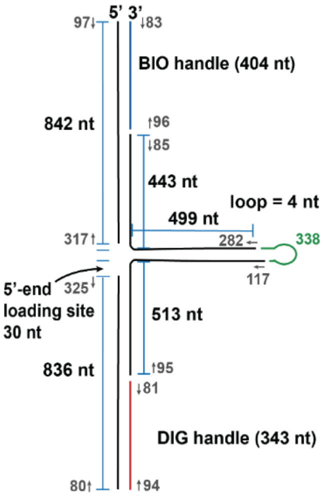 	Primer # 80 - CCTCACTTCTGCTATTTCGC
	Primer # 81 - GCGACTTAGCTGAGGCC
	Primer # 83 - GGAACCAAAGGATATTCAGACG
	Primer # 85 - GGTGCCCACAGAACGTC
	Primer # 94 - TAATACGACTCACTATAGGCCTCACTTCTGCTATTTCGC
	Primer # 95 - TAATACGACTCACTATAGGTCTTCGCCAGACGGCATTTA
	Primer # 96 - TAATACGACTCACTATAGGGCAGGCAAGTCCGATTTTTTG
	Primer # 97 - TAATACGACTCACTATAGGGGAACCAAAGGATATTCAGACG
	Primer # 117 - TGGATCCGTGGGCGC
	Primer # 282 - TAATACGACTCACTATAGTCATGCTCCCATCTTATGG
	Primer # 317 - GCCAACTCGGTCGCGTC
	Primer # 325 - TAATACGACTCACTATAGGCTGACGTTCTATGCAG
	RNA Oligo # 338 - GUAGUGAUUAAAAAUAAUCACUACUGGAUCCGUGGGCGCAGCGGAGAAGAA
	pMK-T (ColE1 ori, KanR) plasmid (ThermoFisher) with a palindromic sequence

Table S2. Parameters for RTC assembly model fits to the VFNA probabilities versus nsp13-helicase concentration. The fractional occupancy of the nsp13-engaged RTC complex from both models was fitted (RTC-nsp13.1* on second row or RTC-nsp13.1,2* on third row) to the VFNA probability versus nsp13-helicase concentration ([nsp13]). (Fourth row) The parameter values for RTC assembly model 2 fitted to the VFNA probability versus nsp13M4 concentration ([nsp13M4]). The Chi-squared (χ^2) value is the sum of the error weighted squares and the cost function. Related to **Table 1**.

Model	Dataset	[nsp13] (nM)	F (pN)	t_{cut} (1/s)	Value type	χ^2	$\Delta G_1(k_BT)$	$\Delta G_{1,2}(k_BT)$	$\Delta G_{1,2^*}(k_BT)$
Assembly model 1; RTC-nsp13.1*	[nsp13] dependency	0-50	20	0.08	**Best fit values**	58932	2.9	-	-1.5
Assembly model 2; RTC-nsp13.1,2*					**Best fit values**	21659	1.5	1.4	-1.0
Assembly model 2; RTC-nsp13.1,2*	[nsp13M4] dependency				**Best fit values**	4195	1.1	1.5	0.1
					**Bounds**		[-3, 3]	[-3, 3]	[-3, 3]

Table S3. Parameter values for the RTC Assembly - Elongation dynamics model. Parameters for the best fit (row 1) and the mean and standard deviation of 100 bootstrap fits (row 2 and 3) to the dwell time distributions for increasing nsp13-helicase concentration with the single nucleotide probabilities and timescales free for the RTC-nsp13.1,2 complex, with only the VSNA characteristic timescale fixed. (row 4) Parameters of the best fit with the single nucleotide probabilities for the RTC-nsp13.1,2 complex free and the single nucleotide timescales fixed to the values for the core RTC. (row 5) Parameters of the best fit keeping all the single nucleotide probabilities and timescales for the RTC-nsp13.1,2 complex fixed to the values for the core RTC. (row 6-8) Parameters for the fits to the dwell time distributions with increasing nsp13M4 concentration with the probabilities and timescales free for the RTC-nsp13.1,2 complex. (row 9) The boundaries on the fitted parameters. *Parameters that are fixed during the fit. Related to **Table 1**.

Model	Conditions	[nsp13](nM)	F(pN)	t_{cut}(s)	Fit conditions	Value type	LL	BIC	ΔG_1 (k_BT)	$\Delta G_{1,2}$ (k_BT)	$\Delta G_{1,2^*}$ (k_BT)
RTC Assembly - Elongation dynamics	[nsp13] dependency	0-50	20	0.08	RTC-nsp13.1,2 single nt probabilities and timescales free	Best fit values	-18372	36885	1.70	0.81	-0.61
						Mean values			1.74	0.87	-0.69
						Std values			0.16	0.09	0.16
					RTC-nsp13.1,2 single nt probabilities free	Best fit values	-19223	38534	2.10	0.99	-1.22
					All single nt parameters fixed	Best fit values	-20291	40614	1.2	-1.57	2.01
	[nsp13M4] dependency				RTC-nsp13.1,2 single nt probabilities and timescales free	Best fit values	-37157	74449	0.76	1.68	-0.07
						Mean values			0.86	1.57	-0.02
						Std values			0.05	0.06	0.05
						Bounds			[-3, 3]	[-3, 3]	[-3, 3]

Value type	$p_{FNA.c}$ (F_0)	$p_{FNA.1,2}$ (F_0)	$p_{SNA.c}$ (F_0)	$p_{SNA.1.2}$ (F_0)	$p_{VSNA.c}$ (F_0)	$p_{VSNA.1,2}$ (F_0)	p_{LLP} (F_0)	τ_{VFNA} $(F_0)(s)$	$\tau_{FNA.c}$ $(F_0)(s)$	$\tau_{FNA.1,2}$ $(F_0)(s)$	$\tau_{SNA.c}(F_0)(s)$	$\tau_{SNA.1,2}(F_0)(s)$	$\tau_{VSNA.c}(F_0)(s)$	$\tau_{VSNA.1,2}(F_0)(s)$
Best fit values	0.89	0.96	0.085	0.036	0.019	0.0006	0.0030	0.02	0.048	0.033	1.60	0.59	4.40	4.79
Mean values	0.89	0.96	0.085	0.036	0.019	0.0008	0.0033	0.02	0.048	0.034	1.56	0.56	4.41	4.78
Std values	0.00	0.00	0.003	0.002	0.002	0.0007	0.0004	0	0.001	0.001	0.04	0.04	0.04	0.01
Best fit values	0.90	0.96	0.081	0.033	0.017	0	0.0033	0.02*	0.05*	0.05*	1.64*	1.64*	4.37*	4.37*
Best fit values	0.9*	0.9*	0.077*	0.077*	0.019*	0.019*	0.0031*	0.02*	0.05*	0.05*	1.64*	1.64*	4.37*	4.37*
Best fit values	0.89	0.94	0.077	0.053	0.028	0.0025	0.0035	0.02	0.048	0.036	2.10	0.61	4.23	4.75
Mean values	0.89	0.94	0.081	0.052	0.026	0.0033	0.0042	0.02	0.048	0.036	2.08	0.59	4.27	4.73
Std values	0.00	0.00	0.005	0.003	0.004	0.0020	0.0005	0.00	0.004	0.001	0.06	0.07	0.07	0.04
Bounds	[0, 1]	[0, 1]	[0, 0.5]	[0, 0.1]	[0, 0.1]	[0, 0.1]	[0, 0.1]	[0.008, 0.1]	[0.02, 0.1]	[0.02, 0.1]	[0.05, 5]	[0.05, 5]		
